# Large-scale genome re-annotation uncovers pathogen-specific lncRNA regulators of fungal growth and virulence

**DOI:** 10.1371/journal.ppat.1014681

**Published:** 2026-10-09

**Authors:** Renwei Gao, Zhenxiu Liu, Han Tang, Ning Wang, Xinying Xue, Ling Lu, Yuanwei Zhang

**Affiliations:** 1 Jiangsu Key Laboratory for Pathogens and Ecosystems, Jiangsu Engineering and Technology Research Centre for Microbiology, College of Life Sciences, Nanjing Normal University, Nanjing, China; 2 Department of Respiratory and Critical Care, Xuanwu Hospital of Capital Medical University, National Clinical Research Center for Geriatric Diseases, Beijing, China; 3 Department of Respiratory and Critical Care, Shandong Second Medical University, Weifang, China; UC Irvine, UNITED STATES OF AMERICA

## Abstract

Invasive fungal infections pose a serious global health threat. Although core cellular processes are broadly conserved across fungi, only a small fraction of known species cause severe human disease, suggesting that pathogenicity may depend in part on pathogen-specific regulators. However, such elements remain poorly understood because they often lack recognizable homologs, limiting accurate annotation and functional characterization. Here, using the invasive mold *Aspergillus fumigatus* as a model, we combined transcriptome-guided re-annotation with functional screening to identify previously unrecognized long non-coding RNAs (lncRNAs) required for fungal growth and virulence. Among 423 loci previously annotated as *Fumigati* section-specific protein-coding genes, 176 were reclassified as non-coding, revealing widespread annotation errors that had obscured potential regulators. We identified two lncRNAs, *SSP7* and *SSP8*, that are required for hyphal growth and virulence in a murine infection model. Mechanistically, *SSP7* binds the coding region of *pprA* mRNA, promotes its association with polysomes and translation, and thereby sustains mitochondrial function, whereas *SSP8* contributes to amino acid homeostasis. Together, these findings demonstrate for the first time that pathogen-specific lncRNAs can fine-tune conserved cellular processes to generate distinct fungal growth and virulence traits, and reveal a novel translational regulatory mechanism in fungal pathogenicity.

## Author summary

Fungal infections are a major threat to human health, yet only a small fraction of fungal species can cause severe disease. We asked whether genetic elements found only in particular groups of pathogenic fungi might contribute to these differences. Using the major human fungal pathogen *Aspergillus fumigatus* as a model, we re-annotated a set of genes previously annotated as protein-coding and found that many had been misannotated. Instead, a large proportion produce long non-coding RNAs (lncRNAs), which do not encode proteins but can regulate cellular functions. We identified two such lncRNAs, *SSP7* and *SSP8*, that are important for fungal growth and virulence. *SSP7* helps maintain translation of a conserved protein required for mitochondrial function, whereas *SSP8* contributes to maintaining balanced amino acid levels. Together, these results suggest that fungal-specific non-coding RNAs can influence fundamental cellular processes and pathogenicity. They also highlight the importance of accurate genome annotation for uncovering previously overlooked regulators in fungal pathogens.

## Introduction

Severe infections caused by fungal pathogens result in more than one million deaths worldwide each year and have become a major threat to public health [1]. However, among the millions of fungal species identified to date, only a small fraction are capable of successfully adapting to the human host and causing severe disease [2]. Taking the genus *Aspergillus* as an example, although it comprises more than 300 species, only a few are known to cause aspergillosis [3,4]. Among clinically isolated *Aspergillus* species, members of the section *Fumigati*, including *A. fumigatus* and its cryptic sibling species, are the predominant cause of both invasive and chronic aspergillosis, accounting for approximately 90% of human aspergillosis cases [4,5]. Previous studies have shown that the conidia of *A. fumigatus* possess remarkable resistance to heat, desiccation, and UV radiation, together with efficient airborne dispersal and deep pulmonary deposition-traits that are thought to contribute to its pathogenic potential [3,6]. However, several non-pathogenic fungal species share similar physiological characteristics yet rarely cause severe human infections [7,8]. The pronounced differences among fungi in growth characteristics, virulence, and host adaptability therefore suggest that additional pathogen-specific genetic regulatory factors beyond highly conserved core cellular processes may constitute the molecular basis underlying the uniquely high pathogenic potential of fungal pathogens.

Advances in comparative genomics and pan-genome analysis have revealed that fungal genomes can generally be divided into core genes and dispensable genes [9]. Core genes are highly conserved across strains or species and are essential for fundamental cellular processes; consequently, research has historically focused on these genes and their conserved regulatory networks [10,11]. In comparison, dispensable genes, including accessory and unique genes, display greater variability and may evolve rapidly with unknown factors. These genes are thought to facilitate fungal adaptation to their unique environmental niches [12,13]. Thus, accessory and unique genes may serve as key genetic determinants that regulate virulence during fungal pathogen infections [14,15]. Among dispensable genes, species- or section-specific genes represent a particularly understudied subset owing to their restricted phylogenetic distribution. A major obstacle in studying these genes lies in their accurate annotation [16]. Current fungal genome annotations rely heavily on automated pipelines and homology-based inferences derived from well-established eukaryotic yeasts or mammals. While such methods perform well for conserved core genes, they are often inadequate for dispensable genes, especially species-specific genes, which frequently lack recognizable homologs and exhibit atypical sequence features [17,18]. As a result, dispensable genes are prone to misannotation or may remain entirely unannotated [19], impeding efforts to elucidate their biological roles. These inaccuracies affect not only protein-coding genes but also non-coding transcripts [20]. Among the latter, long non-coding RNAs (lncRNAs) represent a widespread and important class of regulatory molecules. Like mRNAs, lncRNAs are transcribed by RNA polymerase II, but they lack significant open reading frames and do not encode proteins [21,22]. Although initially regarded as transcriptional noise, lncRNAs are now recognized as key regulators involved in diverse biological processes [23]. However, the landscape and functional roles of lncRNAs in fungi remain largely unknown. Therefore, a major unresolved challenge in fungal pathogenicity research is the accurate identification and functional validation of specific genomic elements, including both protein-coding genes and non-coding transcripts.

In this study, we used the major invasive human fungal pathogen *A. fumigatus* as a model and systematically screened putative section-specific proteins (Ssps) within the *Fumigati* section of *Aspergillus* using comparative genomics and high-quality RNA-seq-based transcript reconstruction. Our analysis revealed widespread transcript annotation errors among *ssp* genes. Coding potential analyses indicated that a substantial fraction of the detected transcripts were likely non-coding RNAs. Consistent with this, we identified similar annotation errors in another clinically important fungal pathogen, *Cryptococcus neoformans*, suggesting that such misannotations are not unique to *A. fumigatus*. Among these candidates, two *Fumigati* section-specific genes, *ssp7* and *ssp8*, whose transcripts displayed classic lncRNA features, were found to be required for hyphal growth and virulence in *A. fumigatus*. These findings highlight the importance of section-specific genes in regulating conserved fungal biological functions. Mechanistically, lncRNA *SSP8* regulates intracellular amino acid homeostasis through interactions with multiple amino acid metabolism-related proteins, whereas *SSP7* directly binds the mRNA of a highly conserved mitochondrial translation factor (PprA) via a specific sequence region, promotes its association with polysomes, enhances translational efficiency, and thereby maintains mitochondrial homeostasis. Together, these results demonstrate that section-specific lncRNAs function as key regulators of fungal physiology and virulence, and that they confer distinct pathogenic potential and environmental adaptability by precisely modulating conserved core cellular processes. Moreover, this study establishes a general framework for systematically correcting genome annotation errors and identifying precise regulatory targets in other fungal pathogens.

## Results

### Transcriptome-guided reannotation reveals hidden lncRNAs among *Fumigati*-specific genes

Given that section-specific factors may underlie pathogenic diversity, we established an evolutionary framework to identify proteins unique to different *Aspergillus* sections by screening all publicly available *Aspergillus* genomes and selecting one representative strain from each species to construct a genus-wide phylogenetic tree. The analyzed *Aspergillus* species were classified into nine distinct sections: *Fumigati*, *Aspergillus*, *Cremei*, *Flavi*, *Nigri*, *Circumdati*, *Candidi*, *Terrei*, and *Nidulantes* (Fig 1A). Through a BLASTP-based comparative genomic analysis of representative strains across the nine sections, we initially screened 914 proteins specific to the *Fumigati* section (Fig 1B). To minimize false positives arising from incomplete sampling of other species, we conducted a second round of BLASTP searches against the NCBI non-redundant (nr) protein database. This two-step filtering confirmed that 423 proteins were specific to the *Fumigati* section, which we designated as *Fumigati* section-specific proteins (Ssps) (Fig 1B).

**Fig 1:**
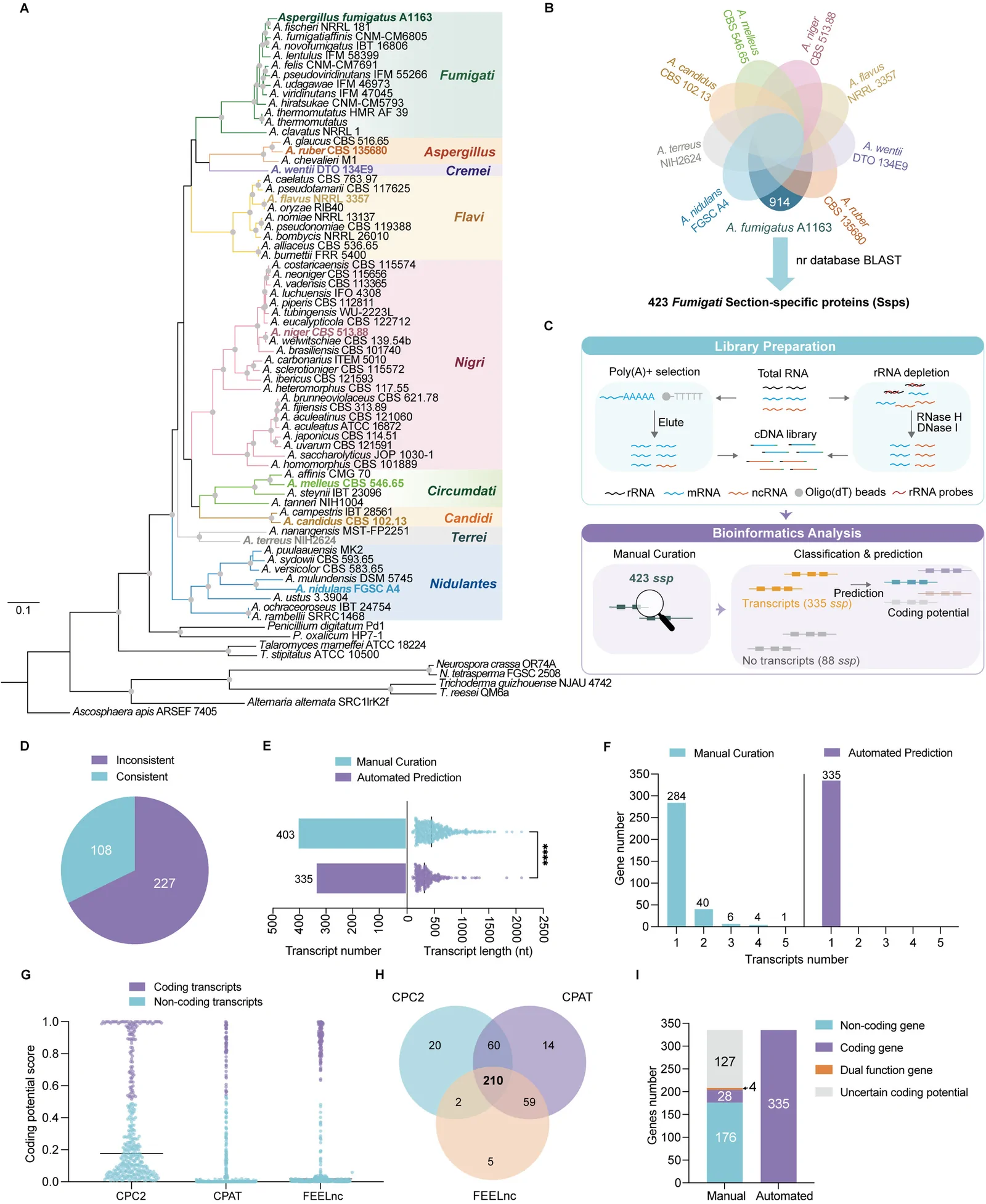
Systematic identification, transcriptome-guided reannotation and coding-potential assessment of *Fumigati* section-specific genes (*ssp*). **(A)** Maximum-likelihood phylogeny of 63 representative *Aspergillus* species. Branch colours correspond to different taxonomic sections. Representative species from each section used for subsequent *Fumigati* section-specific proteins (Ssps) screening are highlighted. **(B)** Pipeline for identifying Ssps. **(C)** Schematic overview of the transcriptome-guided reannotation workflow. Total RNA was subjected to either poly(A)+ selection or rRNA depletion, followed by cDNA library preparation for both methods. After sequencing, transcript structures of 423 initially screened *ssp* genes were manually curated and subsequently used for bioinformatic assessment of coding potential. **(D)** Number of 335 *ssp* genes with detectable full-length transcripts showing consistent or inconsistent transcript structures between automated prediction and manual curation. **(E)** Comparison of transcript numbers and lengths between manual curation and automated prediction for the 335 *ssp* genes with detectable full-length transcripts. The right panel shows the length distribution of individual transcripts, with black lines indicating mean values. Statistical significance was determined using unpaired *t* test. ^****^P < 0.0001. **(F)** Distribution of transcript isoform numbers per gene based on manual curation or automated prediction for the 335 *ssp* genes with detectable full-length transcripts. **(G)** Coding-potential scores of 403 corrected transcripts assessed using CPC2, CPAT and FEELnc. Transcripts classified as coding or non-coding by each tool are shown, and black lines indicate median values. The cutoff values for coding probability were set as follows: 0.5 for CPC2, 0.544 for CPAT, and 0.61 for FEELnc. **(H)** Venn diagram showing the overlap of transcripts identified as non-coding by CPC2, CPAT and FEELnc. **(I)** Summary of gene classifications obtained through manual curation compared with automated prediction for the 335 *ssp* genes with detectable full-length transcripts.

Although these candidates are annotated as protein-coding genes in existing genome annotations, species- or section-specific genes typically lack recognizable homologs and often exhibit complex exon-intron structures [18,24]. As a result, automated annotation pipelines, which heavily rely on homology-based inference and parameters trained on model organisms, frequently generate inaccurate ORF predictions and may erroneously assign non-coding transcripts as protein-coding genes [17,25]. Therefore, to determine whether these Ssps truly encode proteins, we performed a systematic transcript reconstruction and manual re-annotation for all candidate genes. To obtain an accurate transcript map for this validation, we constructed *A. fumigatus* RNA-seq libraries using both poly(A)+ selection and rRNA depletion methods (workflow shown in Fig 1C). The poly(A)+ selection approach is widely used for RNA-seq library construction to analyze mRNA expression but fails to capture non-polyadenylated transcripts, including many fungal ncRNAs [26]. To overcome this limitation, we also prepared rRNA-depleted libraries that retain both polyadenylated and non-polyadenylated transcripts despite higher background noise. Integrating both datasets provided a more comprehensive and unbiased transcriptome for accurate identification of all section-specific genes (*ssp*) transcripts.

Substantial curation of all 423 *ssp* genes revealed that only 335 genes had full-length transcripts under the tested culture conditions. Surprisingly, approximately 68% of these genes exhibited transcript structures that were inconsistent with the automated predictions (Fig 1D). Further analysis identified that 403 corrected transcripts were produced by the 335 genes (Fig 1E). These curated transcripts were significantly longer than the automated predictions, and several genes generated multiple transcript isoforms (Fig 1E and 1F). To systematically evaluate the coding potential of these corrected transcripts, we performed a comprehensive analysis using three widely used coding-potential prediction tools: CPC2, CPAT, and FEELnc. Surprisingly, all three tools revealed that approximately half of the corrected transcripts were consistently classified as non-coding (Fig 1G and 1H). At the gene level, only 28 of the 335 *ssp* genes were classified as protein-coding, whereas 176 genes were categorized as non-coding, and 4 genes produced both coding and non-coding transcripts, whereas the remaining genes showed inconsistent coding-potential predictions (Fig 1I). These results suggest that *ssp* genes in *A. fumigatus* are widely misannotated by automated pipelines, and a substantial proportion of them may function as non-coding RNAs (ncRNAs) rather than protein-coding genes.

### Functional screening identifies two *Fumigati*-specific loci required for fungal growth and virulence

To generate a focused set of candidates for initial functional screening, we examined the curated annotations of the 25 *ssp* genes that automated pipelines had originally predicted to encode secreted proteins. We designated these genes as *ssp1* to *ssp25* (S1 Table). Manual re-curation revealed that only 16 of the 25 candidates produced full-length transcripts under the tested conditions; coding-potential analysis classified four as protein-coding, eight as non-coding, and four as ambiguous (S1 Table). To assess whether these candidates are induced during infection, we analyzed their expression under infection-related conditions by culturing *A. fumigatus* in mouse lung-homogenate medium (Mice Lung). Most of the 25 *ssp* genes were significantly upregulated at 12 or 24 h compared with cultures grown in minimal medium (MM), whereas *ssp4* and *ssp23* showed no significant induction (Fig 2A). These expression patterns suggest that the upregulated *ssp* genes may be involved in adaptation to host-associated conditions. We therefore proceeded to investigate their biological functions experimentally.

**Fig 2:**
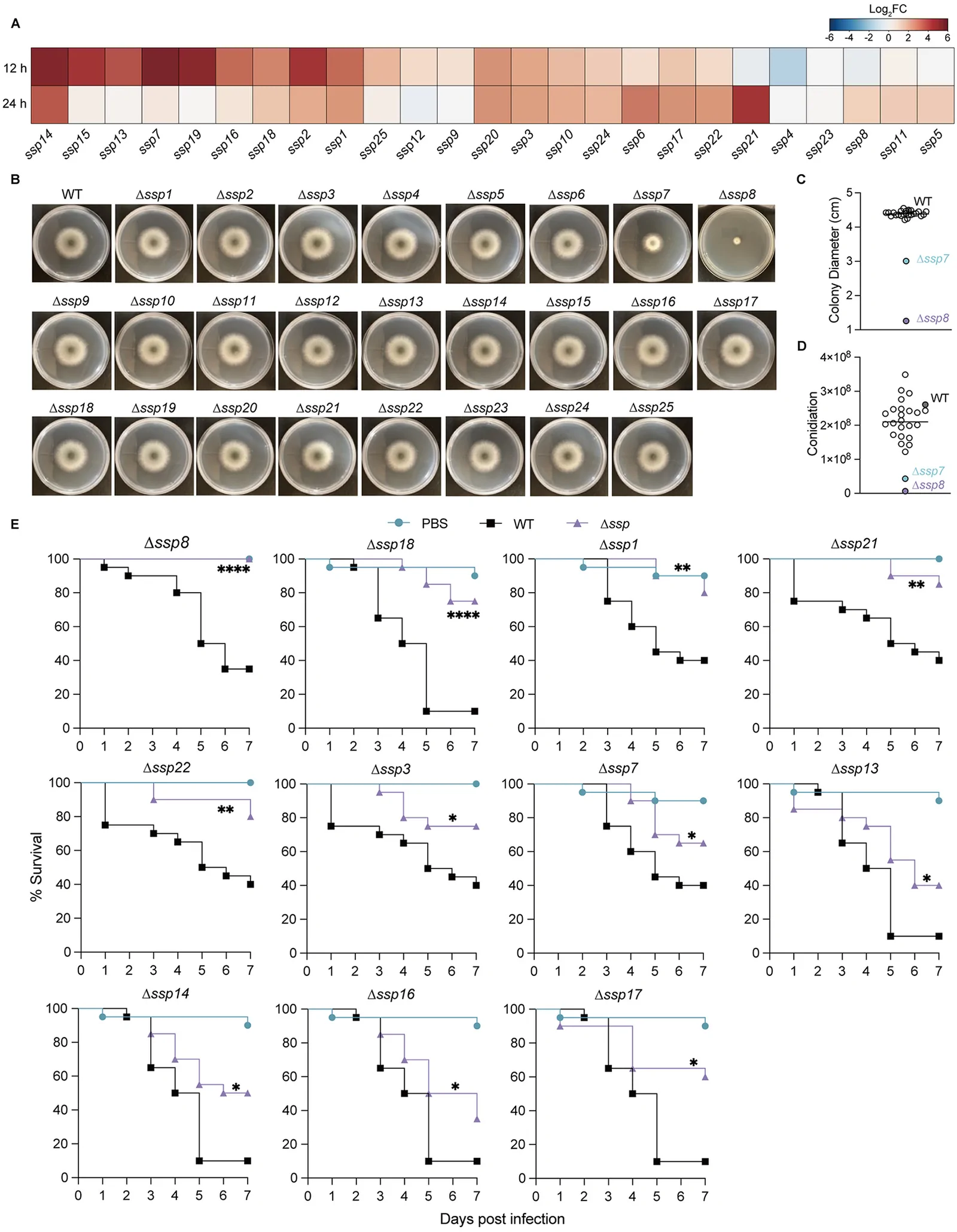
Expression profiling and phenotypic characterization of 25 curated *ssp* gene-deletion mutants in *A. fumigatus.* **(A)** Heatmap showing fold-change (FC) in expression of 25 curated *ssp* genes at 12 h and 24 h in mouse lung-homogenate medium (Mice Lung) relative to minimal medium (MM). Colors indicate log_2_FC values. **(B)** Colony morphology of the wild-type and 25 Δ*ssp* mutants grown on solid minimal medium (MM) at 37°C for 66 h. **(C)** Quantification analysis of colony diameters of the indicated strains grown on MM plates. Points represent mean values (n = 6). **(D)** Quantification analysis of conidiation of the indicated strains grown on MM plates. Points represent mean values (n = 3). **(E)** Virulence assessment of related Δ*ssp* mutants in *G. mellonella* infection models. Survival curves of *G. mellonella* larvae infected with conidia of wild-type (WT), each Δ*ssp* mutant, or PBS control. Each panel represents an individual *ssp* gene deletion strain. Survival was monitored for 7 days post-infection (n = 20). Mutants exhibiting significantly attenuated virulence compared with WT are indicated. Statistical significance was determined using the log-rank (Mantel-Cox) test. ^*^P < 0.0332, ^**^P < 0.0021, ^***^P < 0.0002, ^****^P < 0.0001.

Accordingly, we constructed mutants for all 25 *ssp* genes, each confirmed by diagnostic PCR (S1A and S1B Fig). For *ssp8*, a complete deletion could not be obtained; therefore, an *ssp8* truncation mutant was used for subsequent functional analyses and is hereafter referred to as Δ*ssp8*. Among the mutants, only Δ*ssp7* and Δ*ssp8* exhibited pronounced growth defects on minimal medium (Fig 2B-2D). Virulence assays using a *Galleria mellonella* infection model further revealed that 11 mutants (Δ*ssp1*, Δ*ssp3*, Δ*ssp7*, Δ*ssp8*, Δ*ssp13*, Δ*ssp14*, Δ*ssp16*, Δ*ssp17*, Δ*ssp18*, Δ*ssp21*, and Δ*ssp22*) displayed significantly attenuated pathogenicity (Fig 2E and S2 Fig). Together, these results indicate that *ssp7* and *ssp8* play critical roles in both fungal growth and virulence, establishing a direct link between these section-specific genes and pathogenic potential.

### *SSP7* and *SSP8* are *Fumigati*-specific lncRNAs required for growth, stress adaptation, and virulence

Disruption of *ssp7* and *ssp8* caused pronounced defects in both fungal growth and virulence. Because these loci were initially identified among putative *Fumigati*-specific protein-coding genes, their broad phenotypic effects were unexpected and prompted us to select them for further characterization. Given their reclassification as putative ncRNAs through transcriptome-guided reannotation, we next sought to experimentally clarify their molecular nature. PCR amplification followed by Sanger sequencing confirmed that the actual cDNA sequences of both gene*s* were fully consistent with the manually curated annotations but differed substantially from the automated predictions (Fig 3A and 3B), supporting the high accuracy of the manual re-annotations. Both genes were significantly upregulated under host-mimicking conditions, yet neither protein Ssp7 nor Ssp8 was detected in proteomic datasets under any tested conditions (Fig 3C and 3D). Subsequently, we constructed C-terminal FLAG-tagged versions of both genes and performed protein expression analysis. No detectable bands of FLAG-tagged Ssp7 and Ssp8 were found at their predicted sizes, and a previously reported Rtt109-FLAG strain served as a positive control (Fig 3E) [27]. These results suggest that neither *ssp7* nor *ssp8* encodes a functional protein.

**Fig 3:**
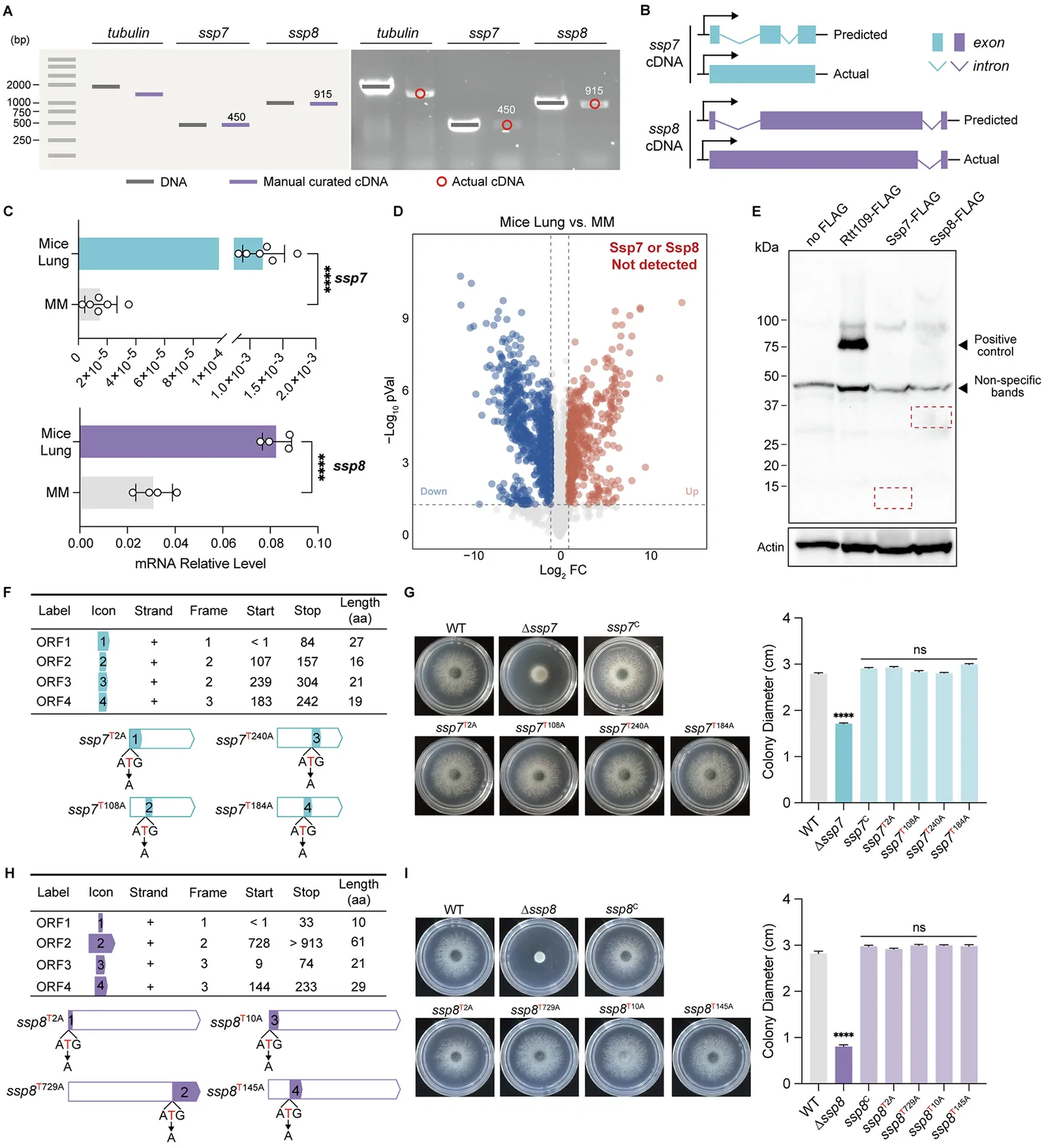
Experimental validation of the non-coding nature of *ssp7* and *ssp8* and functional assessment of ORF-disrupting mutants. **(A)** PCR amplification of genomic DNA and cDNA for *ssp7* and *ssp8* using primers spanning their full-length transcripts. Manually curated transcript sizes are indicated, and experimentally obtained cDNA bands are marked with red circles. *tubulin* served as a control. **(B)** Schematic comparison of predicted and actual exon-intron structures for *ssp7* and *ssp8*. **(C)** Relative mRNA expression of *ssp7* and *ssp8* in the wild-type strain cultured in minimal medium (MM) and mouse lung-homogenate medium (Mice Lung), quantified by RT-qPCR. Statistical significance was determined using unpaired *t* test (n ≥ 4). ^****^P < 0.0001. **(D)** Volcano plot of proteomic comparison of the wild-type strain cultured in MM (control) and Mice Lung (treatment). **(E)** Western blot analysis of FLAG-tagged Ssp7- and Ssp8-expressing strains. Actin served as a loading control. **(F, H)** Predicted ORFs within the *ssp7*/*ssp8* genomic locus. Four predicted ORFs and their reading frames are shown with start and stop positions. Point mutations (ATG → AAG) introduced into each predicted ORF are indicated. **(G, I)** Colony morphology and diameters of related strains grown on solid MM medium at 37°C for 48 h. Representative images are shown alongside quantification of colony diameters (n = 6). Statistical analysis was performed using one-way ANOVA with multiple comparisons tests. ^****^P < 0.0001; ns, not significant.

To exclude the possibility that their function depends on short peptides encoded by the transcripts, we analyzed four putative short open reading frames (ORFs) in each gene and mutated their start codons from ATG to AAG (Fig 3F and 3H), a strategy previously shown to block translation initiation in fungi [28]. The mutated *ssp7* and *ssp8* fragments were then transformed into the corresponding deletion mutants (Δ*ssp7* and Δ*ssp8*) through ectopic integration, confirmed by diagnostic PCR and sequencing. As expected, all tested transformants, similar to the complemented strain, fully restored the growth defects of the deletion mutants (Fig 3G and 3I), indicating that site-directed mutations for putative translation initiation of *ssp7* and *ssp8* did not affect their ability to rescue the mutant phenotypes and suggesting that neither gene functions via translation of short peptides. These results further demonstrated that both *ssp7* and *ssp8* function as ncRNAs rather than proteins. Given their transcript lengths exceeding 200 nucleotides, we classified them as lncRNAs and designated them *SSP7* and *SSP8* following the naming conventions used for lncRNAs in *Candida auris* and *Cryptococcus neoformans* [28,29].

To further characterize the subcellular distribution of these lncRNAs, we performed nuclear-cytoplasmic fractionation followed by semi-quantitative RT-PCR. *SSP8* was detected in both the nuclear and cytoplasmic fractions. Because *SSP7* was expressed at very low levels under standard culture conditions and could not be reliably detected in the wild-type strain after fractionation, its distribution was examined using an *ssp7*-overexpression strain. *SSP7* was likewise detected in both nuclear and cytoplasmic fractions (S3A and S3B Fig).

Since *ssp7* and *ssp8* do not encode proteins, we next examined whether these genes remain specific to the *Fumigati* section. BLASTN searches against the 63 *Aspergillus* genomes (Fig 1A) supported that both genes, despite their non-coding features, remain restricted to species within the *Fumigati* section (S4A Fig), indicating that *ssp7* and *ssp8* are truly section-specific, and subsequent genome-wide screening and phylogenetic analysis revealed that both genes are present and highly conserved across all selected *A. fumigatus* strains. Notably, *ssp7* sequences segregated into two evolutionary clades distinguished by a single SNP (S4B Fig). Deletion of *ssp7* in the non-Δ*ku80* Af293 background caused a pronounced growth defect similar to that observed in the A1163Δ*ku80* background (S4C Fig), further supporting a conserved role of *ssp7* in fungal growth. In comparison, *ssp8* exhibits highly conserved sequences across all examined strains and forms a single evolutionary clade (S4D Fig).

To assess their functional roles, we examined growth and stress responses of Δ*ssp7* and Δ*ssp8* mutants. Compared with the WT and complemented strains, both mutants exhibited pronounced growth defects under tested culture conditions, with particularly strong sensitivity to most stress treatments (Fig 4A and 4B). In addition, disruption of either gene resulted in markedly reduced colony growth rates, decreased biomass, and delayed germination (Fig 4C-4E). We next examined the contribution of *ssp7* and *ssp8* to virulence in a murine infection model. Consistent with the attenuated pathogenicity observed in the *G. mellonella* infection model, deletion of either *ssp7* or *ssp8* markedly attenuated fungal virulence, whereas genetic complementation restored virulence to WT-like levels, demonstrating that these two section-specific lncRNAs are required for pathogenicity of *A. fumigatus* (Fig 4F). Together, reannotation and functional assays confirmed that *ssp7* and *ssp8* encode lncRNAs rather than proteins. These lncRNAs are *Fumigati* section-specific, evolutionarily conserved in *A. fumigatus*, and required for fungal growth, stress adaptation and virulence.

**Fig 4:**
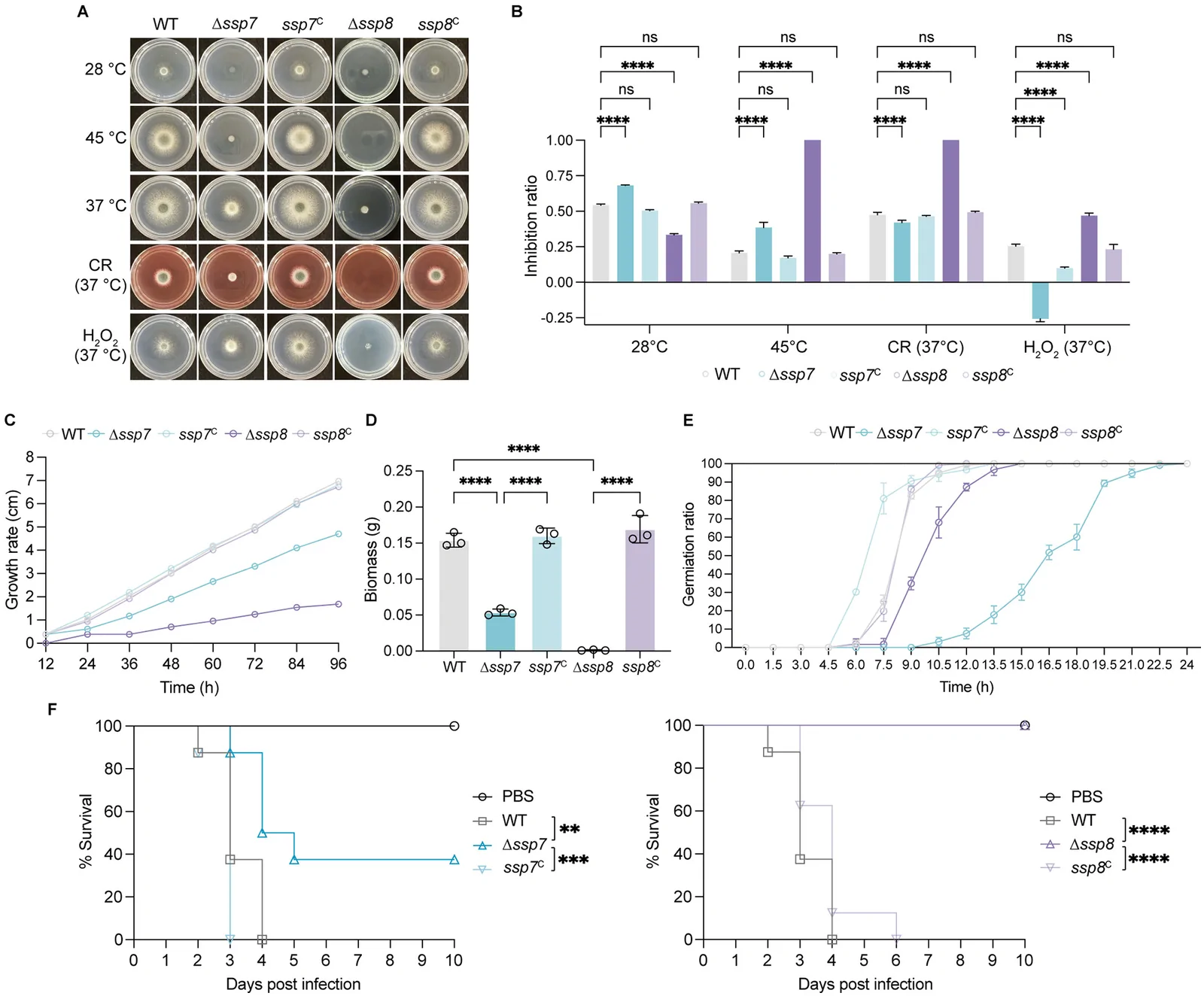
*SSP7* and *SSP8* contribute to growth, stress adaptation, germination, and virulence in *A. fumigatus.* **(A)** Colony morphology of related strains grown on minimal medium (MM) under the indicated conditions for 48 h, including 28°C, 45°C, 37°C, Congo red (CR) stress (15 μg/mL) at 37°C, and H_2_O_2_ stress (2 mM) at 37°C. **(B)** Growth inhibition ratios of related strains cultured under the indicated conditions. n = 6. **(C)** Radial growth of related strains on MM at 37°C, measured at the indicated time points. n = 8. **(D)** Mycelial dry weight of related strains grown in liquid MM at 37°C with shaking at 220 rpm for 48 h. n = 3. **(E)** Conidial germination of related strains at 37°C, monitored at the indicated time points. n = 3, with at least 180 conidia examined in total for germination analysis. **(F)** Virulence assessment of related strains in a murine infection model. PBS-injected mice were used as negative controls (n = 8, with equal group sizes maintained within each experimental batch). Statistical significance was determined using the log-rank (Mantel-Cox) test. ^**^P < 0.0021; ^****^P < 0.0001; ns, not significant. **(B, D)** Statistical analysis was performed using one-way ANOVA with multiple comparisons tests. ^****^P < 0.0001; ns, not significant.

### *SSP8* regulates amino acid metabolic homeostasis

Given that *SSP7* and *SSP8* are lncRNAs restricted to the *Fumigati* section, it was surprising that their deletion severely affected the growth and virulence of *A. fumigatus*. Such fundamental functions are usually governed by highly conserved core genes in fungi. This unexpected observation prompted us to further investigate their regulatory targets and underlying mechanisms. We first compared the proteomic profiles of the Δ*ssp8* mutant and the wild-type strain (Fig 5A). Gene Ontology (GO) enrichment analysis revealed that the differentially expressed proteins were significantly enriched in amino acid metabolism (Fig 5B), suggesting that *SSP8* may participate in the regulation of related pathways. We further performed an RNA pull-down assay to identify proteins interacting with *SSP8*. Considering that lncRNA functions are often strand-specific [22], we synthesized the sense and antisense *SSP8* labeled with desthiobiotin. The labeled RNAs were then used to enrich RNA-protein complexes, thereby enabling identification of proteins that associate directly or indirectly with each strand (workflow shown in Fig 5C).

**Fig 5:**
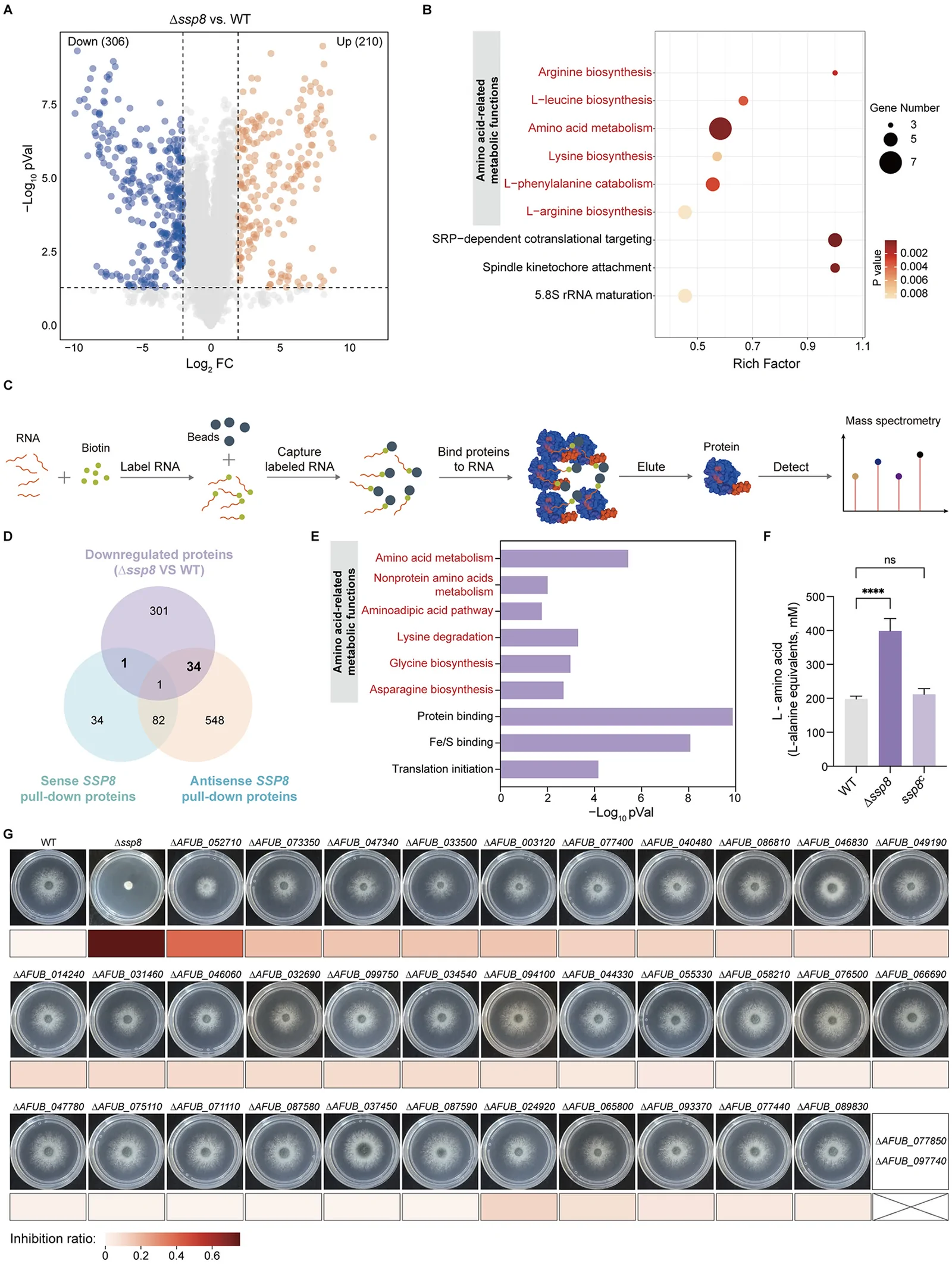
Identification of *SSP8*-interacting proteins and functional characterization of potential downstream genes. **(A)** Volcano plot showing differentially expressed proteins (DEPs) between the Δ*ssp8* mutant and wild-type (WT) strain. DEPs were defined using thresholds of p-value < 0.05 and |log_2_FC| ≥ 2. **(B)** Gene Ontology (GO) enrichment analysis of DEPs identified in the Δ*ssp8* mutant compared to WT. **(C)** Schematic overview of the RNA pull-down workflow. **(D)** Venn diagram comparing downregulated proteins in Δ*ssp8* with proteins identified from *SSP8* sense and antisense strand RNA pull-down assays. DEPs were filtered using p-value < 0.1 and log_2_FC ≤ -2, and pull-down-enriched proteins were selected using a Sum PEP Score ≥ 0.5. **(E)** Functional Catalogue (FunCat) enrichment analysis of proteins specifically associated with *SSP8* sense or antisense strand. **(F)** Quantification of L-amino acid levels (measured as L-alanine equivalents) in related strains. n = 6. Statistical analysis was performed using one-way ANOVA with multiple comparisons tests. ^****^P < 0.0001; ns, not significant. **(G)** Growth phenotypes of single-gene deletion mutants corresponding to putative *SSP8*-interacting proteins. The heatmap indicates the inhibition ratio of each deletion mutant relative to WT, calculated based on colony growth.

Among the proteins pulled down by either the *SSP8* sense or antisense strand, 35 proteins (one interacting with the sense strand and 34 interacting with the antisense strand) were also downregulated in the Δ*ssp8* mutant compared with the wild type in our proteomic analysis, suggesting that they may represent downstream targets of *SSP8* (Fig 5D). To assess the RNA-binding potential of the candidate proteins, we performed Pfam and InterPro domain analyses. None of the 35 candidate *SSP8*-interacting proteins contained canonical RNA-binding domains. However, AFUB_037450 and AFUB_094100 showed putative noncanonical RNA-binding features, suggesting that these proteins may represent higher-priority candidates for further validation of *SSP8*-associated RNA-protein interactions (S4 Table). Further integration of the transcriptomic and proteomic data for these 35 candidate proteins revealed that several proteins were markedly reduced at the protein level without corresponding decreases in their transcript abundance (S5B Fig). These findings indicate that the effects of SSP*8* on protein abundance cannot be fully explained by transcriptional regulation alone and suggest the involvement of additional post-transcriptional regulatory mechanisms.

Individual gene deletions revealed that 24 mutants exhibited only modest growth defects and did not recapitulate the severe growth defect of Δ*ssp8* (Fig 5G and S6 Fig). Notably, two genes, *AFUB_077850* and *AFUB_097740*, could not be disrupted despite multiple attempts, suggesting that they are potentially essential candidate genes. Together, mass spectrometry and Functional Catalogue (FunCat) enrichment analysis revealed that proteins specifically bound to the sense and antisense strands of *SSP8* were significantly enriched in amino acid metabolic processes (Fig 5E). Consistent with the proteomic and RNA pull-down results, further analysis indicated that there was a markedly high accumulation of intracellular free amino acids (L-amino acids) in the Δ*ssp8* mutant compared with the wild-type strain, supporting a role for *SSP8* in regulating amino acid metabolism (Fig 5F). Among the 24 genes whose deletion caused detectable growth impairment, *AFUB_024920*, *AFUB_065800*, *AFUB_093370*, *AFUB_077440*, and *AFUB_089830* participate directly or indirectly in amino acid metabolism (Fig 5G). In addition, the potentially essential candidate *AFUB_077850* encodes a TauD/TfdA-like domain protein involved in unconventional amino acid metabolism, and may be a key factor underlying the severe growth and pathogenic defects of the Δ*ssp8* mutant. Thus, these results suggest that *SSP8* affects growth and virulence of *A. fumigatus* at least in part by modulating amino acid metabolism.

### *SSP7* targets PprA to regulate protein translation for mitochondrial function

Using a similar approach, we next investigated the regulatory mechanism of *SSP7*. Comparative proteomic analysis between Δ*ssp7* and WT revealed that the differentially expressed proteins were primarily associated with mitochondria-related biological processes (Fig 6A and 6B). RNA pull-down assays showed that proteins pulled down by the *SSP7* sense and antisense strands were significantly enriched in translation-related biological processes (Fig 6C). These findings suggest that *SSP7* may modulate mitochondrial function by interacting with translation-associated proteins. To identify key downstream targets, we focused on proteins that were both bound by *SSP7* and downregulated in Δ*ssp7*, and identified a single sense strand-interacting protein encoded by *AFUB_026570* (Fig 6D). Domain prediction indicated that AFUB_026570 contains pentatricopeptide repeat (PPR) domains, which are canonical RNA-binding domains (S4 Table). Although its function in filamentous fungi has not yet been reported, we named the gene *pprA* and the encoded protein PprA following the *Aspergillus* nomenclature convention. To validate the interaction between *SSP7* and PprA, we labeled PprA with a FLAG tag at the C-terminus under its native promoter and detected binding between the *SSP7* sense strand and PprA in RNA pull-down assays (Fig 6E).

**Fig 6:**
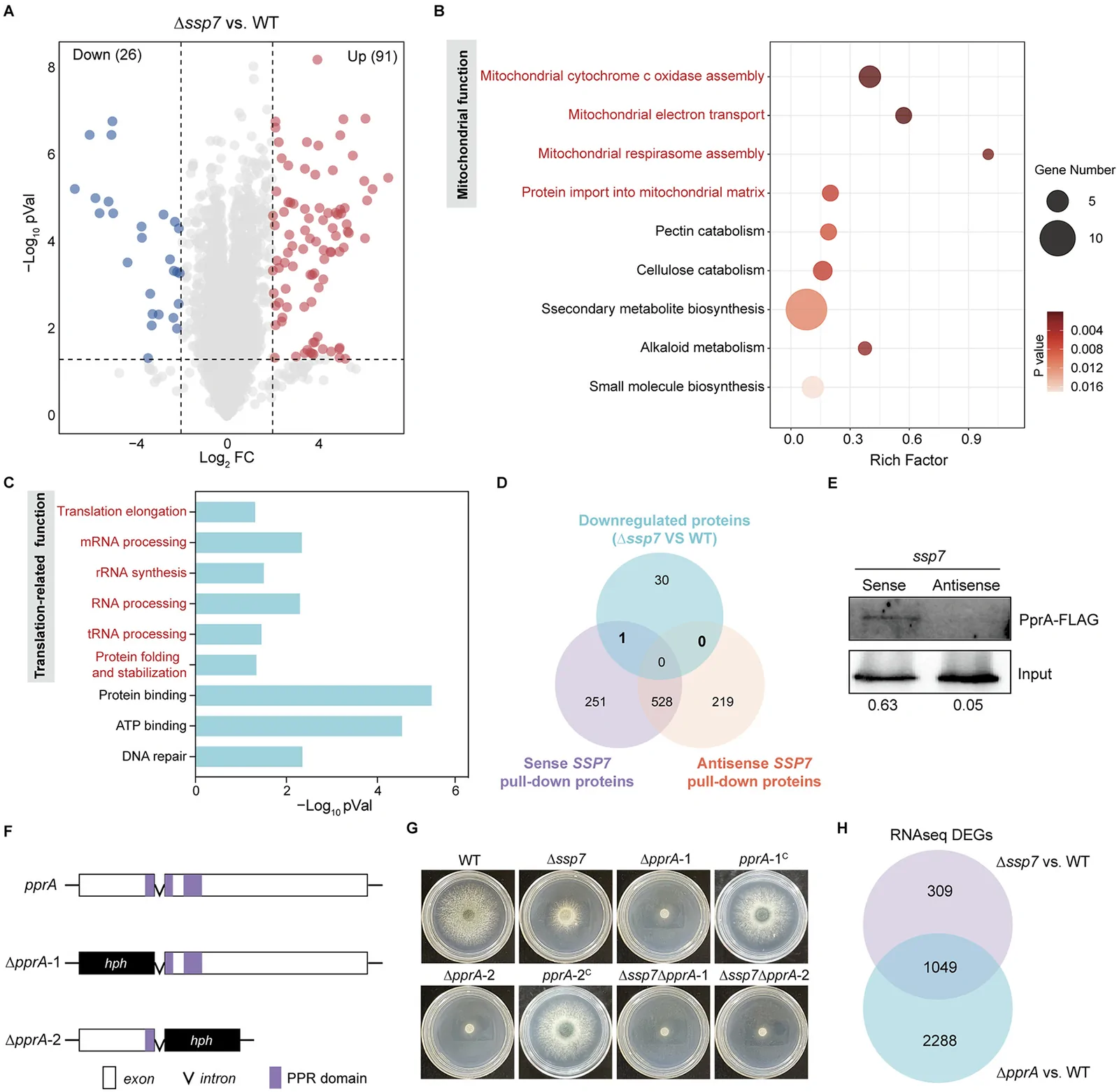
Identification of *SSP7*-interacting proteins and potential downstream regulatory targets. **(A)** Volcano plot showing differentially expressed proteins (DEPs) between the Δ*ssp7* mutant and wild-type (WT) strain. DEPs were defined using thresholds of p-value < 0.05 and |log_2_FC| ≥ 2. **(B)** Gene Ontology (GO) enrichment analysis of DEPs identified in the Δ*ssp7* mutant compared to WT. **(C)** Functional Catalogue (FunCat) enrichment analysis of proteins specifically associated with *SSP7* sense or antisense strand. **(D)** Venn diagram comparing downregulated proteins in Δ*ssp7* with proteins identified from *SSP7* sense and antisense strand RNA pull-down assays. DEPs were filtered using p-value < 0.1 and log_2_FC ≤ -2, and pull-down-enriched proteins were selected using a Sum PEP Score ≥ 0.5. **(E)** RNA pull-down assay validating the interaction between *SSP7* sense strand and FLAG-tagged PprA protein. Input represents the total amount of PprA protein used for each pull-down reaction. Numbers below the blot indicate the relative PprA-FLAG signal intensity in the pull-down fraction normalized to the corresponding input. **(F)** Schematic representation of the *pprA* gene structure and the construction strategy for Δ*pprA*-1 and Δ*pprA*-2 truncation mutants. **(G)** Colony morphology of related strains grown on solid MM at 37°C for 48 h. **(H)** Venn diagram showing the overlap of differentially expressed genes (DEGs) identified in Δ*ssp7* and Δ*pprA* strains compared with WT. DEGs were defined using thresholds of q-value < 0.05 and |log_2_FC| ≥ 2.

BLASTP searches across 1345 fungal genomes revealed that PprA homologs are present in 1122 species (~83%) (S7A Fig), indicating that PprA is highly conserved among fungi. The *A. fumigatus* PprA is homologous to the general mitochondrial translation factor PPR2 (CAA18408.1) from *Schizosaccharomyces pombe*, and the PprA-GFP fusion in *A. fumigatus* confirmed its mitochondrial localization (S7B Fig). Attempts to generate a full *pprA* deletion mutant were unsuccessful, so we performed domain truncation to explore its functional regions (Fig 6F). As shown in Fig 6G, deletion of the PPR domains (Δ*pprA*-1 and Δ*pprA*-2) caused severe colony growth defects, whereas the corresponding complemented strains (*pprA*-1^C^ and *pprA*-2^C^) restored colony growth to levels comparable to that of the wild-type strain. In addition, introducing the truncated *pprA* fragments into the Δ*ssp7* background produced severe colony growth defects similar to the *pprA* truncation mutants (Fig 6G), suggesting that the PPR domains are major determinants of the double-mutant phenotype.

Interestingly, RNA-seq analysis demonstrated that deletion of *ssp7* led to 1358 differentially expressed genes (DEGs), 77% (1049 genes) of which overlapped with the DEGs caused by *pprA* truncation (3337 DEGs in the Δ*pprA* mutant) (Fig 6H), supporting a functional connection between *SSP7* and PprA. Therefore, these results suggest that PprA is a key downstream component of the *SSP7*-associated regulatory pathway and may cooperate with *SSP7* in modulating mitochondrial function.

### *SSP7* directly binds *pprA* mRNA and promotes its translation efficiency

With PprA identified as a downstream target of *SSP7*, we next investigated whether *SSP7* regulates PprA expression at the mRNA or protein levels. Notably, western blot analysis of PprA-FLAG fusion strains revealed that deletion of *ssp7* resulted in a pronounced reduction in PprA protein abundance, while complementation restored protein levels to near wild-type levels (Fig 7A). RT-qPCR analysis showed that *pprA* mRNA levels were even significantly increased in Δ*ssp7* compared with the wild-type strain, consistent with the transcriptome-proteome integrated analysis showing discordant changes between *pprA* transcript and PprA protein abundance (Fig 7B and S5A Fig). This discrepancy between protein and mRNA levels indicates that *SSP7* primarily regulates PprA production at the translational level. To assess whether altered protein degradation also contributes to the reduced PprA abundance, we next examined proteasome-mediated degradation. Treatment with MG132, a proteasome inhibitor that blocks ubiquitin-mediated protein degradation, led to a slight restoration of PprA levels in Δ*ssp7* (Fig 7C), indicating a modest contribution of proteasome-mediated degradation. These results prompted us to ask whether loss of *SSP7* more generally impacts translational activity.

**Fig 7:**
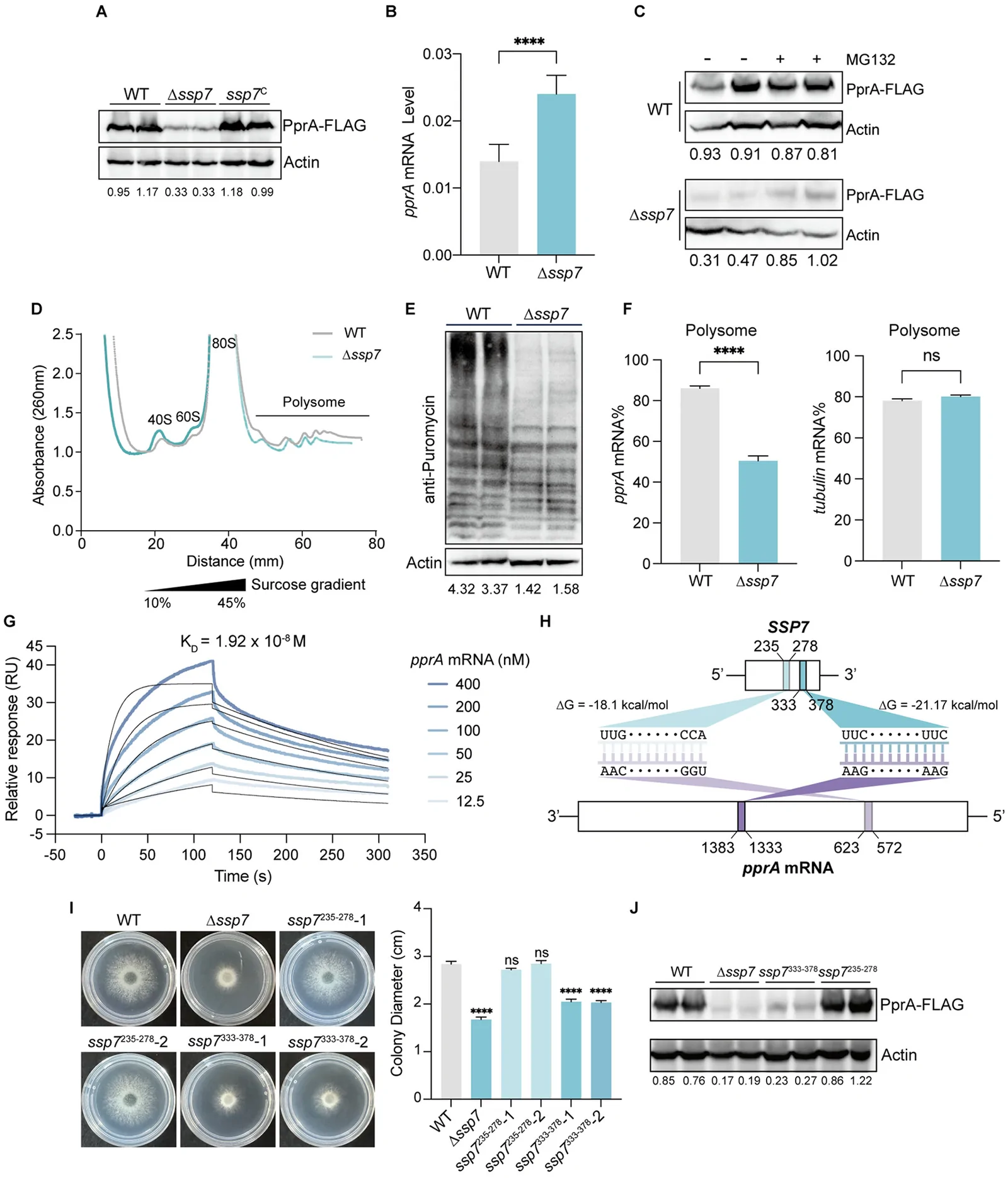
*SSP7* regulates PprA protein abundance through translational regulation. **(A)** Western blot analysis of PprA-FLAG fusion proteins in the wild-type (WT), Δ*ssp7* and *ssp7*^C^ strains. Numbers below the blots indicate the relative PprA-FLAG signal intensity normalized to Actin. **(B)** Relative expression levels of *pprA* mRNA in WT and Δ*ssp7* strains. Expression was quantified by RT-qPCR (n = 8). **(C)** Western blot analysis of PprA-FLAG protein abundance in WT and Δ*ssp7* strains treated with or without the proteasome inhibitor MG132. Numbers below the blots indicate the relative PprA-FLAG signal intensity normalized to Actin. **(D)** Polysome profiling of WT and Δ*ssp7* strains separated on a 10-45% sucrose gradient. Absorbance at 260 nm was monitored across the gradient to distinguish 40S, 60S, 80S, and polysome fractions (n = 2). **(E)** Puromycin incorporation assay of WT and Δ*ssp7* strains to assess global translation activity. Numbers below the blot indicate the total puromycin-incorporated nascent peptide signal intensity normalized to Actin. **(F)** Distribution of *pprA* mRNA and *tubulin* mRNA in polysome fractions from WT and Δ*ssp7* strains, determined by RT-qPCR. n = 4. **(G)** Surface plasmon resonance (SPR) sensorgrams showing the binding interactions between *pprA* mRNA and immobilized *SSP7* across a concentration gradient. Color gradients represent incremental concentrations of the analyte *pprA* mRNA, and the black traces represent the global fitting curves used to calculate the kinetic parameters. The dissociation constant (K_D_) is shown above the plot. **(H)** Predicted RNA-RNA interaction between *SSP7* and *pprA* mRNA as identified by IntaRNA. Interaction regions and interaction energies (ΔG) are shown. **(I)** Colony morphology and diameters of related strains grown on solid MM medium at 37°C for 48 h. Representative images are shown alongside quantification of colony diameters (n ≥ 6). **(J)** Western blot analysis of PprA-FLAG protein abundance in the indicated *SSP7* scramble strains. Numbers below the blots indicate the relative PprA-FLAG signal intensity normalized to Actin. **(A, C, E, J)** Actin served as a loading control, and the signal intensities of PprA-FLAG and puromycin-labeled proteins normalized to Actin are shown below each panel. Statistical significance was determined using unpaired *t* test **(B, F)** and one-way ANOVA with multiple comparisons tests **(I)**. ^****^P < 0.0001; ns, not significant.

Polysome profiling analysis revealed that the total amount of mRNA bound to polysomes was reduced in the Δ*ssp7* mutant, indicating broadly decreased translational activity (Fig 7D). Consistently, a puromycin labeling assay, which incorporates puromycin into nascent polypeptide chains to directly reflect global protein synthesis, also confirmed this global translational impairment (Fig 7E). Building on this, RT-qPCR of polysome fractions further demonstrated that *pprA* mRNA showed markedly reduced polysome association in the Δ*ssp7* mutant, whereas no significant change was observed for the control gene *tubulin* (Fig 7F). These results suggested that *SSP7* may significantly regulate the *pprA* mRNA translation by affecting its association with polysomes.

We next tested whether *SSP7* directly binds the *pprA* mRNA coding region using a surface plasmon resonance (SPR) assay. *SSP7* exhibited a concentration-dependent binding to the *pprA* mRNA coding region (Fig 7G), and kinetic fitting yielded a dissociation constant (K_D_) of 1.92 × 10^-8^ M, indicating a high-affinity direct interaction. Consistent with this, IntaRNA analysis predicted multiple potential RNA-RNA interaction regions between *SSP7* and the *pprA* coding sequence, among which two regions exhibited the lowest interaction energies (ΔG), with lower ΔG values indicating a stronger propensity for stable RNA-RNA interactions (Fig 7H). To assess the functional relevance of these candidate binding regions, we generated *SSP7* mutants carrying scrambled mutations in the corresponding regions. Scrambling the *SSP7* 235–278 region did not affect colony morphology, whereas scrambling the 333–378 region significantly impaired colony growth (Fig 7I). We further examined PprA protein abundance in these *SSP7*-scrambled strains. Consistent with the growth phenotypes, scrambling the 333–378 region markedly reduced PprA protein levels, whereas scrambling the 235–278 region had no detectable effect compared with the wild-type strain (Fig 7J). These results indicate that the 333–378 region of *SSP7* is critical for *SSP7*-mediated regulation of PprA expression and the associated biological effects. Together, these findings demonstrate that lncRNA *SSP7* directly binds the coding region of *pprA* mRNA to promote its polysome association and translation efficiency, while also partially affecting PprA protein stability.

## Discussion

A long-standing question in microbial pathogenesis is why only a small subset of fungal species exhibit high pathogenicity despite sharing highly conserved core cellular processes with non-pathogenic fungi. To explore this, we focused on pathogen-specific genes as a potential key regulatory layer. However, demonstrating that a gene is truly species-specific remains challenging, not only due to highly divergent genes from a common ancestor but also to inaccurate genome annotation [30,31]. This difficulty is particularly pronounced for non-coding transcripts, which lack recognizable homologs and therefore cannot be functionally annotated by homology [32,33]. Our study systematically reveals that many transcripts previously misannotated as protein-coding genes are in fact lncRNAs with potential functions. We identified section-specific lncRNAs *SSP7* and *SSP8* in a major human fungal pathogen *A. fumigatus* and demonstrated that they play crucial roles in fungal growth and virulence similar to conserved core genes, thereby underscoring the fundamental importance of ncRNAs in regulating fungal physiology and pathogenicity (Fig 8).

**Fig 8:**
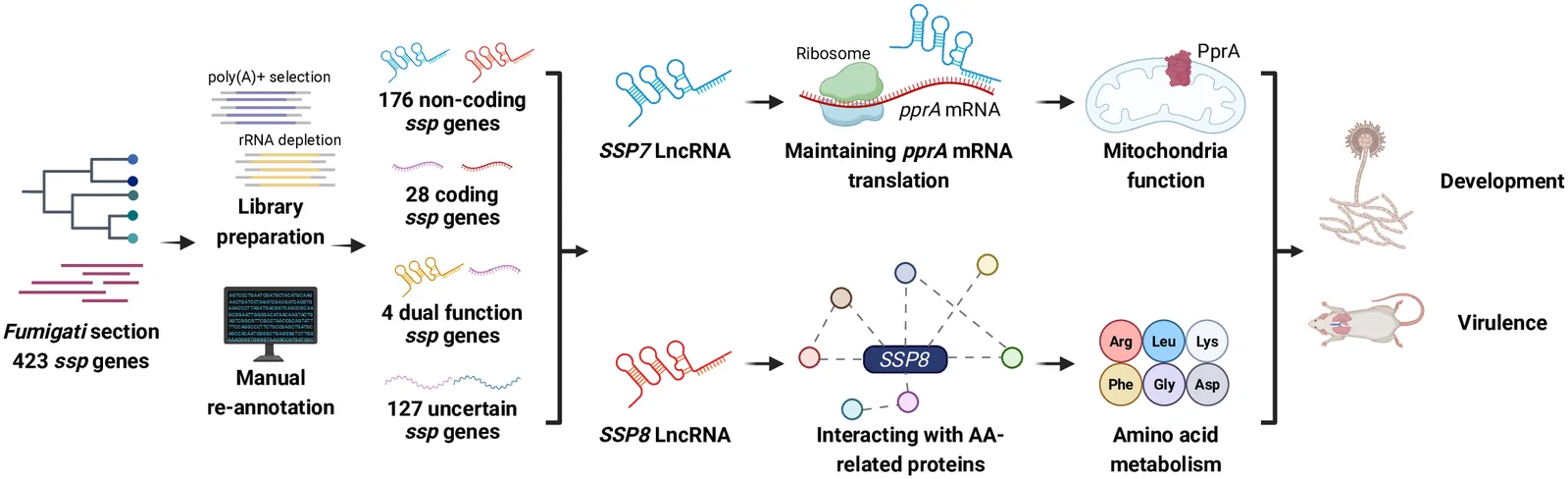
A proposed working model for the identification and functions of *Fumigati* section-specific lncRNAs. Combining poly(A)+-selected and rRNA-depleted RNA-seq with manual re-annotation reclassified 423 *Fumigati* section-specific genes (*ssp*), revealing widespread non-coding *ssp* genes. Functional analyses identified two lncRNAs: *SSP7* supports mitochondrial function by maintaining translation of *pprA* mRNA, whereas *SSP8* interacts with amino acid-related proteins to regulate amino acid metabolism. Together, these lncRNA-mediated pathways promote fungal development and virulence. Created in BioRender. Zhang, Y. (2026) https://BioRender.com/47wpmko.

To date, RNA-seq libraries are commonly generated using poly(A)+ tail selection and enable accurate quantification of protein-coding genes, but this approach fails to capture non-coding RNAs (ncRNAs) that lack poly(A) tails [26,34]. In comparison, the rRNA depletion libraries retain both polyadenylated and non-polyadenylated transcripts, which theoretically provide broader transcriptome coverage but often introduce higher background noise [35,36]. In this study, we employed both library construction strategies in parallel to leverage their complementary advantages, thereby achieving a more complete and unbiased transcriptomic landscape that enabled the identification of transcripts that would otherwise remain undetected in common fungal RNA-seq workflows (Fig 1C). Analysis based on this combined approach revealed widespread transcript annotation errors among section-specific genes of *A. fumigatus*, with more than half of the transcripts misclassified as protein-coding instead of ncRNAs (Fig 1H). Consistent with our findings in *A. fumigatus*, we integrated previously published transcriptomic datasets and manually curated species-specific transcripts in another clinically important fungal pathogen, *Cryptococcus neoformans*. This analysis uncovered similar annotation errors: among 142 species-specific genes, only 81 exhibited full-length transcripts under the tested culture conditions, and approximately 64% of these genes displayed transcript structures that were inconsistent with automated predictions. Notably, 39 loci previously annotated as protein-coding were instead found to encode non-coding RNAs (S8A-S8C Fig). Although these analyses were limited to RNA-seq data generated using only poly(A)+-selected libraries and therefore do not fully capture the transcriptomic landscape, the observation of similar misannotations supports the notion that such annotation errors are widespread in other pathogenic fungal species. Importantly, even in well-studied model fungi *Saccharomyces cerevisiae* and *Aspergillus nidulans*, automated genome annotations still contain numerous systematic errors and incomplete annotations [37,38]. Collectively, these findings emphasize the necessity of integrating high-quality transcriptomic data and substantial experimental curation to achieve accurate identification and annotation of functional genes, especially for section- or species-specific genes.

In filamentous fungi, fundamental biological processes are typically regulated by conserved regulatory pathways such as well-known pathways of the Ca^2+^-calmodulin-calcineurin cascade that governs polarized growth [39], the Cdc/Cdk regulatory network that drives cell cycle progression [40], and the *brlA*-*abaA*-*wetA* central regulatory pathway that controls conidiation [41]. Surprisingly, deletion of section-specific lncRNAs *SSP7* or *SSP8* caused severe defects in *A. fumigatus* growth, with phenotypic impacts comparable to those caused by disrupting conserved core regulatory factors (Figs 2B-2E and 4A-4F). This striking functional importance far exceeds the traditional definition of “dispensable genes” and indicates that section-specific lncRNAs may constitute a previously overlooked but functionally crucial regulatory layer, playing irreplaceable roles in growth. This unexpected combination of section specificity and broad phenotypic effects, together with their effects on virulence, prompted us to further investigate the functions and mechanisms of *SSP7* and *SSP8*. Nevertheless, *in vitro* growth defects should not be considered a direct predictor of virulence, as some *A. fumigatus* mutants with pronounced growth defects can retain virulence comparable to wild type [42]. Thus, other *ssp* loci that affect virulence without obvious growth defects may represent distinct regulators of pathogenicity and warrant further investigation.

Our study reveals that the lncRNA *SSP8* is closely associated with multiple amino acid metabolic pathways, including the biosynthesis of arginine, leucine, and lysine, as well as the catabolism of phenylalanine (Fig 5B). Given the well-established importance of amino acid metabolism for fungal pathogenicity, such as the loss of virulence in *Cryptococcus neoformans* amino acid permeases Aap4/Aap5 deletion mutants and in *A. fumigatus* arginine biosynthetic enzymes ArgJ/ArgB deletion mutants [43–45], together with our observation that disruption of *ssp8* leads to a marked accumulation of intracellular free amino acids (Fig 5F), we hypothesize that loss of *SSP8* impairs amino acid transport or utilization, thereby causing metabolic imbalance. This is consistent with the downregulation of multiple amino acid metabolism-related proteins in the Δ*ssp8* mutant. Such metabolic imbalance may contribute to severe growth defects and complete loss of virulence. Functional analysis of the downstream targets identified *AFUB_077850* as a potentially essential candidate encoding a TauD/TfdA-like domain protein. This protein typically participates in the metabolism of uncommon amino acids such as taurine, thereby linking amino acid homeostasis to the TCA cycle and redox processes [46–48]. Further studies using conditional expression approaches, such as the Tet-On/Tet-Off system, will be required to determine whether *AFUB_077850* is truly essential and to define its role in *SSP8*-mediated regulation. These observations suggest that *SSP8* may serve as a key metabolic node linking amino acid metabolism with the TCA-redox network. Notably, analogous mechanisms of lncRNA-mediated metabolic regulation have been reported in other eukaryotes, including mammalian cancer cells and budding yeasts, where lncRNAs reprogram amino acid metabolism or regulate amino acid transporter activities [49,50].

In comparison, deletion of *ssp7* in *A. fumigatus* caused significant alterations in the expression of numerous mitochondria-associated proteins, particularly those involved in cytochrome c oxidase assembly, electron transport, respirasome assembly, and protein import into the mitochondrial matrix (Fig 6B). Disruption of these core processes likely damages mitochondrial energy metabolism and homeostasis [51–54], thereby contributing to the growth defects and reduced virulence of the Δ*ssp7* mutant. *SSP7* may interact with translation factors or ribosome-associated proteins to fine-tune mitochondrial function. Integrating multi-omics data with functional analyses of downstream targets, we identified PprA as the primary target of *SSP7* (Fig 6D). As a conserved mitochondrial translation factor required for the synthesis of mitochondrial-encoded proteins [55], reduced PprA abundance provides a mechanistic explanation for the significant mitochondrial dysfunction, impaired growth and reduced virulence in the Δ*ssp7* mutant. *SSP7* enhances *pprA* translation by directly binding to *pprA* mRNA and promoting its polysome association (Fig 7D-7J). This represents a previously uncharacterized lncRNA-mediated mechanism of translational regulation in filamentous fungi. Although it remains unclear whether *SSP7*, PprA, and *pprA* mRNA form a stable ternary complex, current evidence indicates that *SSP7* regulates protein expression by modulating *pprA* mRNA translation and PprA protein stability. Importantly, the discovery that section-specific lncRNAs can exert such essential regulatory functions provides a valuable foundation for future antifungal drug-target discovery and opens a new conceptual direction for pathogenicity research.

In conclusion, our study reveals that pathogen-specific lncRNAs can act as essential regulators of fungal growth and virulence by modulating highly conserved cellular processes. This finding expands the conventional view that species- or section-specific genes primarily function as accessory or dispensable elements, and instead positions non-coding transcripts as important contributors to fungal pathogenicity. In addition, our results highlight systematic genome annotation correction as an essential prerequisite for identifying hidden pathogen-specific regulators that may otherwise be overlooked. More broadly, this work provides a conceptual framework for understanding how pathogen-specific non-coding regulators shape conserved cellular functions, and offers a foundation for future exploration of selective antifungal intervention strategies.

## Materials and methods

### Ethics statement

All animal procedures were approved by the Institutional Animal Care and Use Committee of Nanjing Normal University (approval number: IACUC-2024246), and were performed in accordance with the ARRIVE guidelines.

### Screening of putative Ssps in *A. fumigatus*

A local database was constructed by integrating the whole-genome data of *Aspergillus* species and several non-*Aspergillus* fungal strains from the NCBI and FungiDB databases (as of December 2022). Three phylogenetic marker genes (*rpb2*, *benA*, and *cam*) from *A. fumigatus* A1163 were used as reference sequences [56]. Homologous genomic sequences were screened by BLASTN alignment (E-value < 10^-5^). Multiple sequence alignments were performed using the MUSCLE program in AliView 1.23, and then the aligned sequences were concatenated with SequenceMatrix. A phylogenetic tree of *Aspergillus* species was then constructed using the maximum-likelihood (ML) method in IQ-TREE 1.6.12. Based on the resulting phylogenetic topology and established taxonomic frameworks [56,57], we further refined and updated the section classification of *Aspergillus* species (sections represent taxonomic units between genus and species).

For the screening of *Fumigati* section-specific proteins, a protein sequence database was first constructed using representative strains from the other eight sections (including *A. ruber* CBS 135680, *A. wentii* DTO 134E9, *A. flavus* NRRL 3357, *A. niger* CBS 513.88, *A. melleus* CBS 546.65, *A. candidus* CBS 102.13, *A. terreus* NIH2624, and *A. nidulans* FGSC A4). Protein sequences from the *A. fumigatus* A1163 genome were used as queries to search against this database with BLASTP, and non-homologous protein sequences were identified. These non-homologous sequences were then subjected to systematic homology alignment and classification in the NCBI non-redundant protein database using the BLASTP program (E-value < 10^-5^). Proteins uniquely present in strains of the *Fumigati* section were identified and defined as section-specific proteins (Ssps). To further identify potential secreted proteins, the predicted Ssp proteins were analyzed for N-terminal signal peptides using SignalP 6.0 [58].

### Illumina library construction and bioinformatics analysis

#### Illumina library construction.

RNA-seq libraries were constructed using both rRNA depletion and poly(A)+ selection methods. (**I**) rRNA depletion RNA-seq library construction. Total RNA was extracted from the wild-type strain using the E.Z.N.A. Fungal RNA kit (R6840, Omega Bio-Tek) according to the manufacturer’s instructions, with genomic DNA removed by DNase I digestion. RNA concentration was measured using Nanodrop (Thermo Fisher Scientific), and the quality was assessed using Qsep1 (BiOptic). RNA samples with a RIN > 8 were selected for library preparation. Ribosomal RNA was depleted following the method described in references [35,59]. Libraries were then prepared using the VAHTS Universal V8 RNA-seq Library Prep Kit for Illumina (NR605, Vazyme) according to the manufacturer’s instructions. Briefly, the rRNA-depleted RNA was fragmented into short fragments of 150–200 nt using fragmentation buffer, followed by reverse transcription into first-strand cDNA with random hexamer primers. The second-strand synthesis was performed using a dUTP buffer mixture to generate strand-specific libraries. The samples were then mixed with adapters, respectively, for ligation and amplification. The PCR products were purified using 0.9 × DNA Clean Beads (N411, Vazyme), and library amplification was performed using the VAHTS RNA Multiplex Oligos Set1 for Illumina kit (N323, Vazyme). The final libraries were sequenced on an Illumina NovaSeq 6000 platform (OE Biotech, Inc., Shanghai, China) with paired-end 150 bp reads. (**II**) poly(A)+ selection RNA-seq library construction. cDNA libraries were constructed using an oligo(dT) enrichment method and subsequently sequenced on the same Illumina NovaSeq 6000 platform (OE Biotech, Inc., Shanghai, China) with paired-end 150 bp reads.

#### Bioinformatics analysis.

All rRNA depletion and poly(A)+ selection RNA-seq libraries were filtered according to standard quality control procedures [60]. Clean reads were aligned to the *A. fumigatus* reference genome from FungiDB using HISAT2 [61] with the parameter “rna-strandness RF”. Subsequently, genome-guided transcriptome assembly was performed using the Trinity [62] “genome_guided_bam” command. Transcripts from all samples were merged and deduplicated using the StringTie [63] “merge” command. A total of 423 screened *ssp* gene transcripts were manually curated one by one, and the *A. fumigatus* annotation file downloaded from FungiDB was updated locally. The coding potential of the manually curated transcripts was assessed using CPC2 [64], CPAT [65], and FEELnc [66], and the prediction results from these tools were integrated for comprehensive analysis. The cutoff values for coding probability were set as follows: 0.5 for CPC2, 0.544 for CPAT, and 0.61 for FEELnc. For *Cryptococcus neoformans*, previously published RNA-seq datasets generated using poly(A)+-selected library construction were downloaded (wild-type strains grown in liquid YPD medium) and reanalyzed using the same analytical pipeline [67]. For this species, coding probability cutoffs were set to 0.5 for CPC2, 0.446 for CPAT, and 0.61 for FEELnc.

### Strains, media and culture conditions

Unless otherwise specified, experiments were performed using the *A. fumigatus* A1163 Δ*ku80* strain as the wild-type strain [68]. Most strains used in this study were derived from the Δ*ku80* background and are listed in S2 Table. The *A. fumigatus* Af293 strain, a clinical isolate with an intact *ku80* locus, was used as the non-Δ*ku80* background for independent deletion of *ssp7*. Unless otherwise specified, *A. fumigatus* strains were cultured at 37°C in minimal medium (MM) containing 10 g/L glucose, 1 mL/L trace elements, and 50 mL/L 20 × nitrate salts (pH 6.5), as previously described [69]. For solid medium, 1.5% agar was added. Conidia of *A. fumigatus* strains were inoculated into liquid MM at a final concentration of 1 × 10^5^ conidia/mL and cultured at 37°C with shaking at 200 rpm for 24 h. The collected mycelia were used for western blot analysis, proteomic sequencing, Illumina library construction, RNA-seq, RT-qPCR, RNA pull-down assay, quantification of intracellular free L-amino acids and polysome profiling. For host-mimicking culture, mouse lung tissues were homogenized in phosphate-buffered saline (PBS) (E607008, Sangon Biotech) at a weight-to-volume ratio of 1:20 to prepare mouse lung-homogenate medium (Mice Lung). Similarly, conidia were inoculated into the mouse lung-homogenate medium at 1 × 10^5^ conidia/mL and cultured under the same conditions for RT-qPCR or proteomic sequencing.

### Construction of the *A. fumigatus* mutants

The primers used in this study are listed in S3 Table. Construction and transformation of *A. fumigatus* were performed as previously described [69], and transformants were selected on MM containing 200 μg/mL hygromycin B, 25 μg/mL bleomycin sulfate, or 1.5 μg/mL pyrithiamine, depending on the resistance cassette used. The detailed procedures are described below.

#### Single gene knockout strains.

Based on homologous recombination, the target gene knockout fragment was constructed. Approximately 1.2 kb fragments of the upstream and downstream regions of the target gene were amplified from *A. fumigatus* genomic DNA as homologous arms, while a hygromycin B resistance expression cassette (*hph*, approximately 2.6 kb) was also amplified. The three fragments were fused by overlap PCR to construct the knockout fragment, which was then transformed into *A. fumigatus* protoplasts. Since we were unable to obtain complete deletion mutants of *ssp8* and *pprA*, we constructed truncation mutants for subsequent analysis.

#### *ssp7*, *ssp8* and *pprA* complementation strains.

The full-length sequences of the *ssp7*, *ssp8* and *pprA* genes, including their native promoters and terminators, were amplified and fused with the bleomycin sulfate resistance expression cassette (*BleoR*) by overlap PCR, and the resulting fragments were transformed into the protoplasts of Δ*ssp7*, Δ*ssp8*, Δ*pprA*-1 and Δ*pprA*-2 mutants, respectively.

#### Point mutation strains.

The complementation fragments of *ssp7* and *ssp8* were cloned into the SinoMol Zero Blunt Vector (KBT101, SinoMol). Using the resulting plasmids as templates, primers containing the desired point mutations were designed and synthesized, and linearized DNA fragments containing the point mutations were generated by PCR. The template plasmids were then digested with DpnI at 37°C for 2 h. The mutated DNA products were subcloned into the pUC19 plasmid using the ClonExpress MultiS One Step Cloning kit (C113, Vazyme) and subsequently transformed into competent cells of *E. coli* DH5α (C502, Vazyme). All recombinant plasmids were verified by Sanger sequencing to confirm the presence of the expected point mutations. Finally, the *ssp7* and *ssp8* complementation fragments containing the point mutations were amplified by PCR and transformed into the protoplasts of Δ*ssp7* and Δ*ssp8* mutants, respectively.

#### FLAG or GFP tagged strains.

Based on homologous recombination, Ssp8-FLAG, PprA-FLAG, and PprA-GFP fusion expression fragments were generated. The ORF region of the target gene, the FLAG or GFP tag sequence, the *hph* sequence, and the corresponding terminator region were amplified and fused by overlap PCR, ensuring that the FLAG or GFP tag was inserted immediately upstream of the stop codon (TAA/TAG/TGA) of the target gene. PprA-FLAG-tagged strains in the *SSP7* scramble backgrounds were constructed using the same strategy, with the PprA-FLAG fusion fragment introduced into the corresponding *SSP7* scramble strains by homologous recombination. For the construction of the Ssp7-FLAG fusion expression vector, a different strategy was employed. The strong promoter *PgpdA*, the ORF region of *ssp7*, the terminator region of *ssp7*, and *hph* sequence were amplified and fused by overlap PCR, with the FLAG tag inserted immediately before the stop codon of *ssp7*. All fusion PCR products were verified by sequencing and then transformed into *A. fumigatus* protoplasts.

#### Δ*ssp7* strains in the PprA-tagged background.

In the PprA-FLAG and PprA-GFP background strains, the *ssp7*-deletion fragment was generated based on homologous recombination. Approximately 1.2 kb upstream and downstream flanking regions of *ssp7* and the *BleoR* sequence were amplified and fused by overlap PCR. The resulting fragment was transformed into the protoplasts of the PprA-FLAG or PprA-GFP tagged strains.

#### *ssp7* complementation strains in the Δ*ssp7*_PprA-FLAG background.

The full-length sequence of the *ssp7* gene, including its native promoter and terminator, was amplified and fused with the pyrithiamine resistance expression cassette (*ptrA*) by overlap PCR, and the resulting fragment was transformed into the protoplasts of Δ*ssp7*_PprA-FLAG mutant.

#### *pprA* truncation strains in the Δ*ssp7* background.

In the Δ*ssp7* mutant background, the *pprA*-truncation fragment was constructed based on homologous recombination. Approximately 1.2 kb upstream and downstream flanking regions of *pprA* and the *BleoR* sequence were amplified and fused by overlap PCR. The resulting fragment was transformed into the protoplasts of the Δ*ssp7* mutant.

#### Δ*ssp7* strains complemented with scrambled *SSP7* sequences.

Scrambled *SSP7* mutants were generated using a procedure similar to that employed for point mutation strains. Briefly, a plasmid containing the *ssp7* complementation fragment was used as a template, and scramble mutations were introduced by PCR using mutagenic primers, followed by DpnI digestion of the parental plasmids at 37°C for 2 h. The resulting mutated fragments were subcloned into pUC19, verified by Sanger sequencing, and transformed into the Δ*ssp7* protoplasts.

### Virulence assay and ethics statement

#### *G. mellonella* infection model.

The *G. mellonella* virulence assay was performed using a method described previously [70]. Fresh conidia of the indicated *A. fumigatus* strains were collected and resuspended in sterile PBS at a final concentration of 1 × 10^8^ conidia/mL. Healthy *G. mellonella* larvae (approximately 0.3 g each, Tianjin Huiyude Biotechnology Co., Ltd.) were selected, and 10 µL of the conidial suspension was injected into the body through the last right proleg. Larvae in the blank control group were injected with an equal volume of sterile PBS. For each treatment group, 20 larvae were used. After injection, the larvae were incubated in the dark at 37°C, and mortality was recorded every 24 h. Larvae that turned black and showed no movement were considered dead, and survival curves were plotted accordingly. The statistical significance of survival curves was calculated using the log-rank test.

#### Murine infection model.

The murine virulence assay was performed using a method described previously [70]. Fresh conidia of the indicated *A. fumigatus* strains were collected and resuspended in sterile PBS at a final concentration of 1 × 10^8^ conidia/mL. Female C57BL/6J mice (8 weeks old, 20–22 g, Vital River) were used in this study. All animal experiments were performed in the specific pathogen-free (SPF) animal facility of the Laboratory Animal Center of Nanjing Normal University, where cages, bedding, food, and water were provided in accordance with SPF animal housing standards. All animal procedures were performed by research staff who had received training in laboratory animal care and handling. Personnel were trained in mouse handling, intraperitoneal injection, isoflurane anesthesia, intratracheal inoculation, clinical monitoring, humane endpoint assessment, and CO_2_ euthanasia before conducting the experiments. Animal care and procedures were carried out under the supervision of the Laboratory Animal Center of Nanjing Normal University.

Mice were immunosuppressed by intraperitoneal injection of cyclophosphamide (150 mg/kg) on days -4 and -1. On day 0, mice were anesthetized with isoflurane delivered by inhalation and inoculated intratracheally with 30 μL of the conidial suspension. The blank control group received an equal volume of sterile PBS via the same route. Each treatment group consisted of eight mice, with equal group sizes maintained within each experimental batch. Baseline body weight was defined as the body weight measured on day 0 immediately before fungal inoculation, following completion of cyclophosphamide pretreatment. After inoculation, mice were monitored daily for survival, body weight, mobility and general clinical condition. Humane endpoints were predefined to minimize animal suffering. Mice that lost more than 20% of their baseline body weight or displayed markedly reduced mobility were humanely euthanized as soon as they were identified, and no later than the same day, and recorded as dead. A small number of mice were found dead between scheduled daily observations before euthanasia could be performed. Deaths or euthanasia events in the infected groups were considered to be associated with progression of experimental invasive aspergillosis following inoculation with *A. fumigatus* conidia. All remaining mice were humanely euthanized at the end of the experiment. Euthanasia was performed by CO_2_ inhalation. Survival was recorded daily for 10 days, and survival curves were generated to assess strain virulence. The statistical significance of survival curves was calculated using the log-rank test.

### RNA extraction and RT-qPCR

The mycelia were collected, immediately frozen in liquid nitrogen, and ground into a fine powder using a mortar and pestle. Total RNA was extracted using the UNlQ-10 Column Total RNA Purification kit (B511361, Sangon Biotech) according to the manufacturer’s instructions. Following genomic DNA removal with the HiScript III RT SuperMix for qPCR (+gDNA wiper) kit (R323, Vazyme), 1 µg of total RNA was used for cDNA synthesis. RT-qPCR was carried out using ChamQ SYBR qPCR Master Mix (Low ROX Premixed) (Q331, Vazyme) on an ABI QuantStudio 3 Real-Time PCR Instrument (Applied Biosystems). Relative gene expression levels were calculated using the 2^-ΔΔCt^ method, with the housekeeping gene *tubulin* serving as an internal control for each sample to normalize gene expression levels, as described in our previous study [70].

### Proteomic analysis

Total proteins were extracted using the Tris-buffered phenol method, and protein concentrations were determined with the BCA assay. For each sample, 10 μg of protein was used for SDS-PAGE validation, and 50 μg was subjected to trypsin digestion. The digested peptides were desalted and purified, then separated on a Vanquish Neo UHPLC system (Thermo Fisher Scientific, USA). LC-MS/MS analysis was carried out on a timsTOF HT mass spectrometer (Bruker, Germany) in data-independent acquisition (DIA) mode. All DIA raw data were processed and integrated using DIA-NN software, and protein identification and quantification were performed against the *A. fumigatus* A1163 protein database. Differentially expressed proteins were screened using the criteria of p-value < 0.05 and |log_2_FC| ≥ 2. GO enrichment analysis of the differentially expressed proteins was conducted using the hypergeometric distribution algorithm to identify significantly enriched functional terms, and enrichment circle plots were generated with R to visualize the results. Proteomic analysis was performed by Shanghai OE Biotech Co., Ltd. Each experimental group included three independent biological replicates.

### Western blot analysis

The mycelia were collected, immediately frozen in liquid nitrogen, and ground into a fine powder using a mortar and pestle for subsequent protein extraction, as previously described [70]. The protein samples were separated on 4–20% SDS-PAGE gels and then transferred onto polyvinylidene difluoride (PVDF) membranes (Millipore). The membranes were incubated with mouse anti-FLAG (1:5000, F1804, Sigma-Aldrich), rabbit anti-GFP (1:2000, AE078, Abclonal) and rabbit anti-Actin (1:100000, AC026, Abclonal) antibodies at 4°C overnight. After washing three times with PBST, membranes were then incubated with secondary antibody HRP goat anti-mouse IgG (H + L) (1:5000, AS003, Abclonal) or HRP goat anti-rabbit IgG (H + L) (1:5000, AS014, Abclonal) at room temperature for 2 h. After the membranes were washed three times with PBST, the blots were detected using the Enhanced ECL luminescence detection kit (E411, Vazyme) and images were captured with a Tanon 4200 chemiluminescence imaging system. ImageJ software was used to quantify the band intensities.

### Nuclear-cytoplasmic fractionation and RNA analysis

Nuclear and cytoplasmic fractions were prepared from *A. fumigatus* mycelia using a nuclear and cytoplasmic protein extraction kit (C510001, Sangon Biotech) according to the manufacturer’s instructions. The quality of the fractionation was verified by western blot using histone H3 and Actin as nuclear and cytoplasmic markers, respectively. RNA was separately extracted from the nuclear and cytoplasmic fractions using Total RNA Extraction Reagent (R401-01, Vazyme). Following genomic DNA removal with the HiScript III RT SuperMix for qPCR (+gDNA wiper) kit (R323, Vazyme), 1 µg of total RNA was used for cDNA synthesis. The subcellular distributions of lncRNAs *SSP7* and *SSP8* were examined by semi-quantitative RT-PCR using RT-qPCR primers listed in S3 Table. Because *SSP7* was expressed at a very low level in the wild-type strain under standard culture conditions, its subcellular distribution was analyzed using an *ssp7*-overexpression strain. PCR products were separated by agarose gel electrophoresis.

### RNA-seq analysis

Raw reads generated from sequencing were subjected to quality control using FastQC, and low-quality reads were removed with fastp to obtain clean reads for subsequent analysis [60]. Clean reads were aligned to the *A. fumigatus* A1163 reference genome using HISAT2, and gene expression levels were quantified as FPKM. Gene read counts were further calculated using HTSeq-count [71]. Differentially expressed genes (DEGs) were identified with DESeq2 [72], applying the criteria of q-value < 0.05 and |log_2_FC| ≥ 2. GO enrichment analysis of DEGs was performed using a hypergeometric distribution algorithm to identify significantly enriched functional terms, and enrichment circle plots were generated with R. Transcriptome sequencing was performed on the Illumina NovaSeq 6000 platform (OE Biotech, Inc., Shanghai, China). Each experimental group included three independent biological replicates.

### RNA biotin labeling and pull-down assay

The RNA pull-down assay consists of three key steps [73], which are described in detail below.

1) *In vitro* transcription. Full-length cDNA sequences of *ssp7* and *ssp8* containing the T7 promoter sequence (taatacgactcactataggg) were obtained by PCR. To generate both sense and antisense transcripts, the T7 promoter sequence was placed on the forward primer (sense transcription) or the reverse primer (antisense transcription), respectively. *In vitro* transcription was performed using the MEGAscript T7 kit (AM1333, Invitrogen), and the RNA products were purified by phenol-chloroform extraction.
2) RNA biotin labeling**.** The *in vitro* transcribed RNAs *SSP7* and *SSP8* were biotinylated at their 3′ ends using the Pierce RNA 3′ End Desthiobiotinylation kit (20163, Thermo Scientific). Following labeling, the RNAs were purified using chloroform:isoamyl alcohol (24:1).
3) RNA pull-down assay. The assay was performed using the Pierce Magnetic RNA-Protein Pull-Down kit (20164, Thermo Scientific). Antisense RNAs of *SSP7* and *SSP8* were used as controls. The protein extracts were prepared as previously described, with minor modifications [74]. Specifically, *A. fumigatus* mycelial powder was suspended in prechilled lysis buffer containing 10 mM Tris-HCl (pH 7.5), 150 mM NaCl, 0.5 mM EDTA, 1 mM DTT, and 0.01% Triton X-100. Immediately before use, PMSF and protease inhibitor cocktail (P1020, Beyotime Biotechnology) were added to the lysis buffer at a volume ratio of 100:1:1. The samples were vortexed thoroughly and incubated on ice for 40 min, followed by centrifugation at 13000 rpm for 5 min at 4°C. The supernatant was collected as the total protein extract. Biotin-labeled RNAs were first incubated with streptavidin magnetic beads at room temperature for 30 min to facilitate binding. Total protein extracts of *A. fumigatus* prepared using a salt extraction method were then added, and the mixture was incubated at 4°C for 60 min to allow specific binding between RNAs and their potential interacting proteins. After incubation, the beads were washed multiple times with wash buffer to remove non-specifically bound proteins and impurities. Finally, the RNA-protein complexes were eluted from the beads using a high-salt or SDS-containing buffer. The eluates were collected, and proteins were separated by SDS-PAGE. Targeted proteins were further identified by mass spectrometry to determine the specific interaction partners of *SSP7* and *SSP8*.

### Quantification of intracellular free L-amino acids

Intracellular free L-amino acids were measured using the Amplex Red L-Amino Acid Assay Kit (S0271, Beyotime) following the manufacturer’s instructions [75]. Cell lysates were prepared using BeyoLysis Buffer A, and fluorescence was detected at 570 nm. Amino acid concentrations were determined based on an L-alanine standard curve.

### Polysome profiling assay

Cycloheximide (100 μg/mL) was added into liquid MM and incubated with mycelia for 5 min, after which the mycelia were immediately collected and flash-frozen in liquid nitrogen. Detection and separation of ribosomal fractions (40S, 60S, 80S, and polysome) in the polysome profiling analysis were performed as described previously [76] and were carried out by Guangzhou Geneseed Biotech Co., Ltd. Each experimental group included two independent biological replicates. RNA was extracted from the returned fractions (B511361, Sangon Biotech), and RT-qPCR was used to analyze the distribution of *pprA* mRNA in polysome fractions from both wild-type and Δ*ssp7* mutant strains to evaluate its translational efficiency.

### Surface plasmon resonance (SPR) assay

SPR assay was performed at room temperature using a Biacore 1k system (Cytiva) to quantitatively assess the binding affinity between *SSP7* and *pprA* mRNA, as previously described with modifications [77]. *SSP7* and *pprA* mRNA were synthesized by *in vitro* transcription with the MEGAscript T7 kit (AM1333, Invitrogen) and purified using phenol/chloroform extraction. The transcribed *SSP7* RNA was subsequently biotinylated at the 3′ end using the Pierce RNA 3′ End Biotinylation kit (20160, Thermo Scientific). Biotinylated *SSP7* was diluted to 100 ng/μL in HEPES buffer (20 mM HEPES, 150 mM NaCl, 5 mM MgCl_2_·6H_2_O, 0.05% (v/v) Tween-20, pH 7.4) and immobilized on a Series S Sensor Chip SA (Cytiva). Gradient dilutions of *pprA* mRNA prepared in the same buffer were injected across the chip surface, and binding affinity was measured using the multi-cycle kinetics/affinity method. Data fitting and analysis were performed with Biacore 1K Insight Evaluation software using the multi-cycle kinetics evaluation method.

### Fluorescence microscopy

To visualize the subcellular localization of the PprA-GFP fusion protein, a total of 1 × 10^5^ conidia of the PprA-GFP strain were inoculated onto glass coverslips containing 500 μL of liquid MM and cultured at 37°C for 10 h. The medium was then removed, and the samples were gently washed three times with PBS. The samples were fixed with 4% (vol/vol) paraformaldehyde (E672002, Sangon Biotech) at room temperature for 30 min, followed by three additional PBS washes. Mitochondria were stained by incubating the samples with MitoTracker Red CMXRos (C1035, Beyotime) dissolved in PBS at a final concentration of 50 nM for 20 min at room temperature, as described in our previous study [78]. After incubation, the MitoTracker solution was removed, and the coverslips were washed three times with PBS. Finally, fluorescence images were captured using a Zeiss Axio Imager A1 microscope (Carl Zeiss).

### Statistical analysis

Statistical analyses other than the bioinformatic analyses described above were performed using GraphPad Prism v10.0.3. The figure legends provide detailed statistical information, including exact sample sizes (n), measures of statistical signiﬁcance, and the specific statistical tests applied.

## Supporting information

S1 File Raw images. (PDF)

S1 Fig Construction and PCR verification of the 25 *ssp* gene deletion mutants in *A. fumigatus.* (A) The strategy for generating *ssp* gene deletion mutants by homologous recombination in *A. fumigatus*. For each *ssp* gene, the coding sequence region was replaced with a hygromycin B resistance (*hph*) cassette using 5′ and 3′ flanking arms. Primer pairs F2/R2 and F3/R3 were designed to amplify the 5′ and 3′ junction regions, respectively, while F4/R4 was used to detect the presence or absence of the *ssp* genes. (B) PCR validation of all 25 *ssp* gene deletion strains. For each Δ*ssp* mutant, three PCR reactions were performed: (i) F2-R2 confirming correct integration at the 5′ junction, (ii) F3-R3 confirming integration at the 3′ junction, and (iii) F4-R4 confirming deletion of the *ssp* gene. Wild-type (WT) and the corresponding Δ*ssp* mutant strains were analyzed in parallel. Successful deletion mutants show positive amplification for both junction PCRs (F2-R2 and F3-R3) and no product for gene detection (F4-R4). Markers (bp) are shown on the left of each gel image.
(TIF)

S2 Fig Virulence assessment of Δ*ssp* mutants in *G. mellonella* infection models. Survival curves of *G. mellonella* larvae infected with conidia of wild-type (WT), each Δ*ssp* mutant, or PBS control. Each panel represents an individual *ssp* gene deletion strain. Survival was monitored for 7 days post-infection (n = 20). Mutants exhibiting significantly attenuated virulence compared with WT are indicated.
(TIF)

S3 Fig Subcellular distribution of lncRNAs *SSP7* and *SSP8* and reciprocal RNA immunoprecipitation of PprA-GFP. (A) Nuclear-cytoplasmic fractionation followed by semi-quantitative RT-PCR analysis of *SSP8* in the indicated strains. Fractionation efficiency was confirmed by immunoblotting for Histone H3 and Actin as nuclear and cytoplasmic markers, respectively. (B) Nuclear-cytoplasmic fractionation followed by semi-quantitative RT-PCR analysis of *SSP7* in the indicated strains. Fractionation efficiency was confirmed by immunoblotting for Histone H3 and Actin as nuclear and cytoplasmic markers, respectively. (C) Reciprocal RNA immunoprecipitation using PprA-GFP. PprA-GFP was immunoprecipitated with anti-GFP magnetic beads, followed by RNA extraction and RT-PCR detection of *SSP7*. WT was used as the negative control.
(TIF)

S4 Fig Evolutionary conservation of *ssp7* and *ssp8* in *A. fumigatus.* (A) Conservation of *ssp7* and *ssp8* across nine sections within the genus *Aspergillus*. Presence/absence analysis of *ssp7* and *ssp8* across 63 representative *Aspergillus* genomes shown in Fig 1A. (B) Conservation of *ssp7* across *A. fumigatus* strains. Maximum-likelihood phylogeny of *ssp7* nucleotide sequences from 603 *A. fumigatus* isolates reveals two clades (Clade I and Clade II). Representative strains A1163 (Clade I) and Af293 (Clade II) are highlighted. Sequence logo shows the single diagnostic SNP distinguishing the two clades. (C) Deletion of *ssp7* in representative strains from both clades results in a comparable colony growth defect. Colony morphology, radial growth, and conidiation of 293 (*A. fumigatus* Af293 wild-type strain), 293Δ*ssp7*, and complemented strain (293*ssp7*^C^) (n ≥ 4). Statistical analysis was performed using one-way ANOVA with multiple comparisons tests. ^****^P < 0.0001. (D) Conservation of *ssp8* across *A. fumigatus* strains. A maximum-likelihood phylogeny generated from 603 *A. fumigatus* isolates demonstrates that *ssp8* is highly conserved, with no detectable sequence divergence between A1163 and Af293.
(TIF)

S5 Fig Integrated transcriptomic and proteomic analysis of Δ*ssp7* and Δ*ssp8* mutants. (A) Comparison of mRNA and protein abundance changes in the Δs*sp7* mutant. Each point represents a gene quantified at both the transcript and protein levels, with log_2_ fold changes in mRNA abundance shown on the x-axis and corresponding protein abundance changes shown on the y-axis. Genes were classified according to a threshold of |log_2_FC| ≥ 1 as concordant mRNA-protein changes, opposite mRNA-protein changes, mRNA-specific changes, protein-specific changes, or no significant change. AFUB_026570 (*pprA*/PprA) is highlighted. (B) Comparison of mRNA and protein abundance changes in the Δ*ssp8* mutant using the same classification scheme. The boxed region is enlarged below to show candidate *SSP8*-associated proteins previously identified by integrated RNA pull-down and proteomic analyses. Labeled genes indicate these candidate proteins.
(TIF)

S6 Fig Colony diameters of putative *SSP8*-interacting candidate gene-deletion strains grown on MM at 37°C for 48 h. Statistical analysis was performed using one-way ANOVA with multiple comparisons tests (n = 6). ^****^P < 0.0001; ^***^P < 0.001; ^**^P < 0.01; ^*^P < 0.05; ns, not significant.
(TIF)

S7 Fig Phylogenetic analysis and subcellular localization of the PprA protein. (A) Maximum-likelihood phylogenetic tree of PprA proteins constructed from 1122 fungal species. (B) Subcellular localization of PprA-GFP in *A. fumigatus* hyphae. Fluorescence images show PprA-GFP (green) and MitoTracker-stained mitochondria (red), accompanied by differential interference contrast (DIC) and merged channels. Scale bar, 10 μm.
(TIF)

S8 Fig Coding-potential assessment of species-specific genes in *Cryptococcus neoformans.* (A) Number of 81 species-specific genes with detectable full-length transcripts showing consistent or inconsistent transcript structures between automated prediction and manual curation. (B) Coding-potential scores of corrected transcripts assessed using CPC2, CPAT and FEELnc. Transcripts classified as coding or non-coding by each tool are shown, and black lines indicate median values. The cutoff values for coding probability were set as follows: 0.5 for CPC2, 0.446 for CPAT, and 0.61 for FEELnc. (C) Summary of gene classifications obtained through manual curation compared with automated prediction for the 81 species-specific genes with detectable full-length transcripts.
(TIF)

S1 Table Summary of automated cDNA predictions and actual cDNA lengths for the 25 *ssp* genes. (DOCX)

S2 Table All strains used in this study. (DOCX)

S3 Table All primers used in this study. (DOCX)

S4 Table Pfam and InterPro domain annotation and RNA-binding potential of candidate *SSP7*- and *SSP8*-interacting proteins. (XLSX)
