# Supplementary material for: Large-scale genome re-annotation uncovers pathogen-specific lncRNA regulators of fungal growth and virulence
Source: PLoS Pathog. 2026 Oct 9;22(10):e1014681. doi: 10.1371/journal.ppat.1014681 (PMC13652153; doi:10.1371/journal.ppat.1014681)

Fig 3A

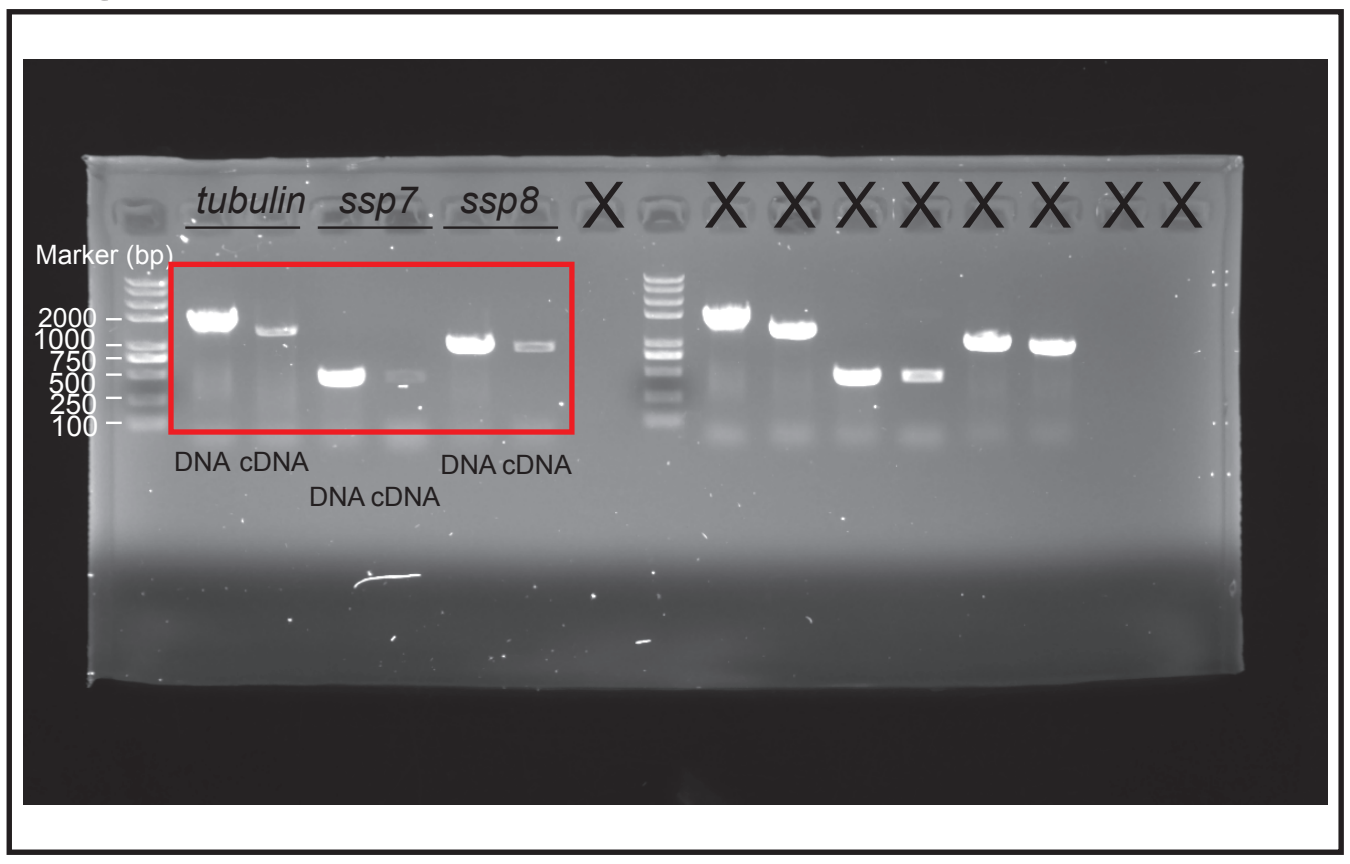

Fig 3E

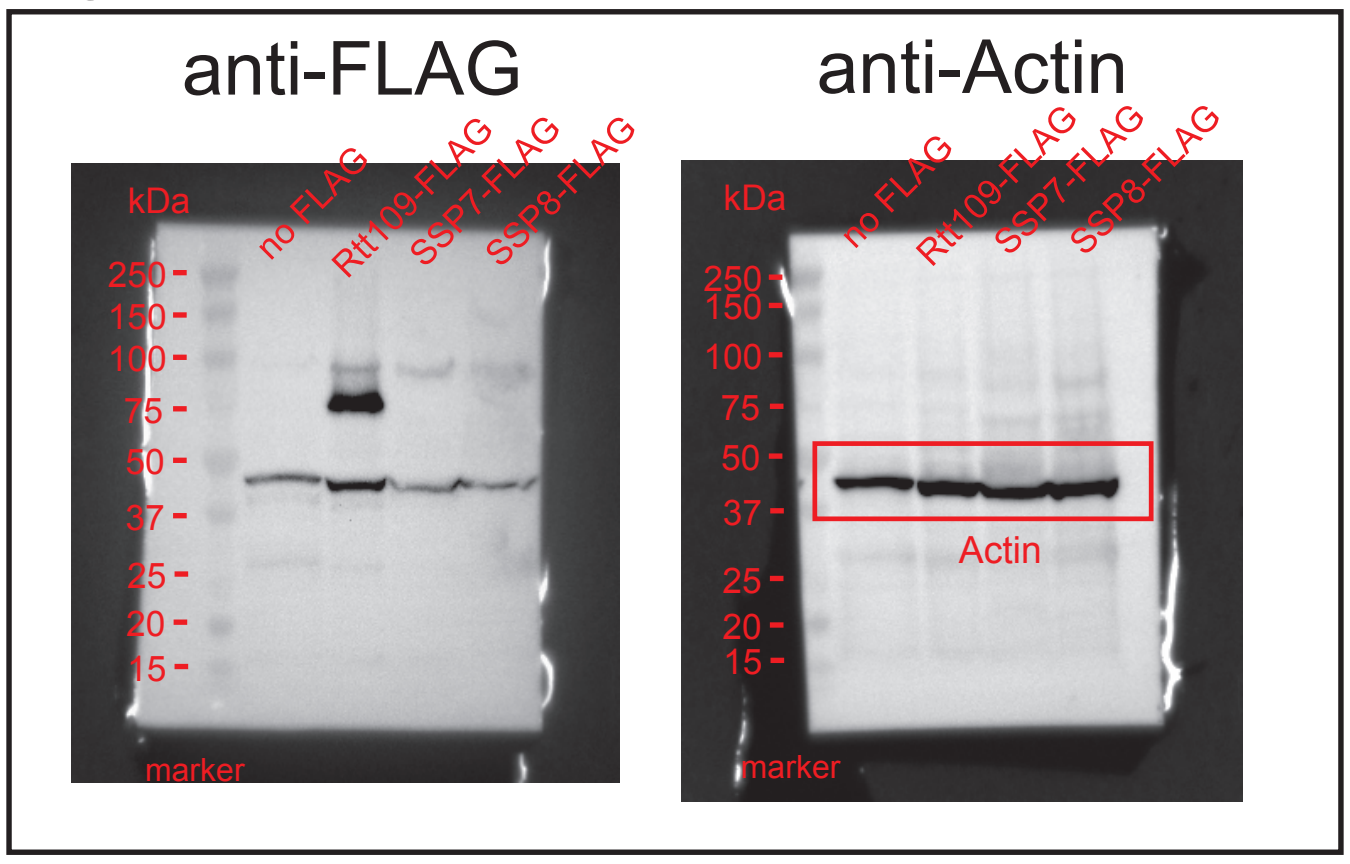

Fig 6E

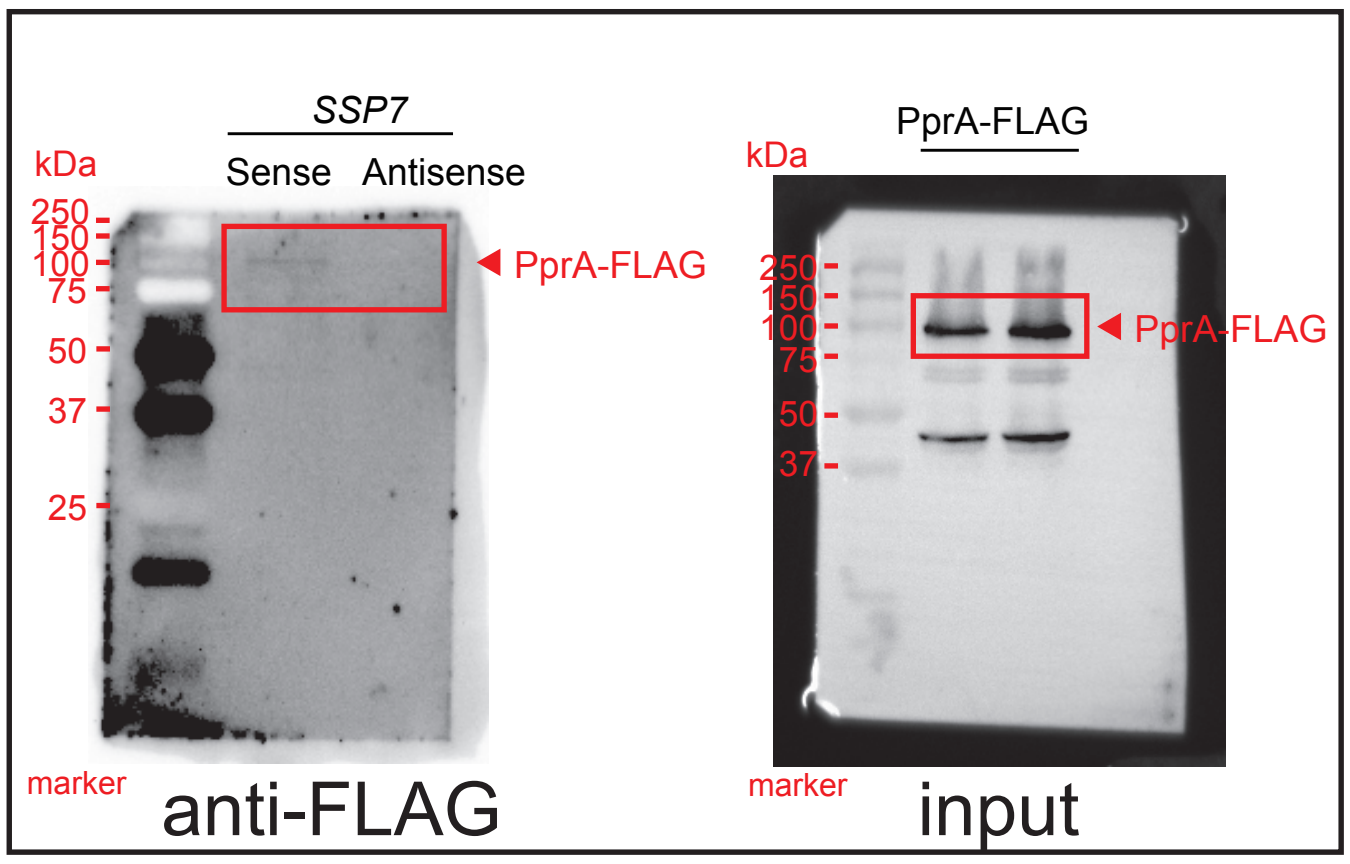

Fig 7A

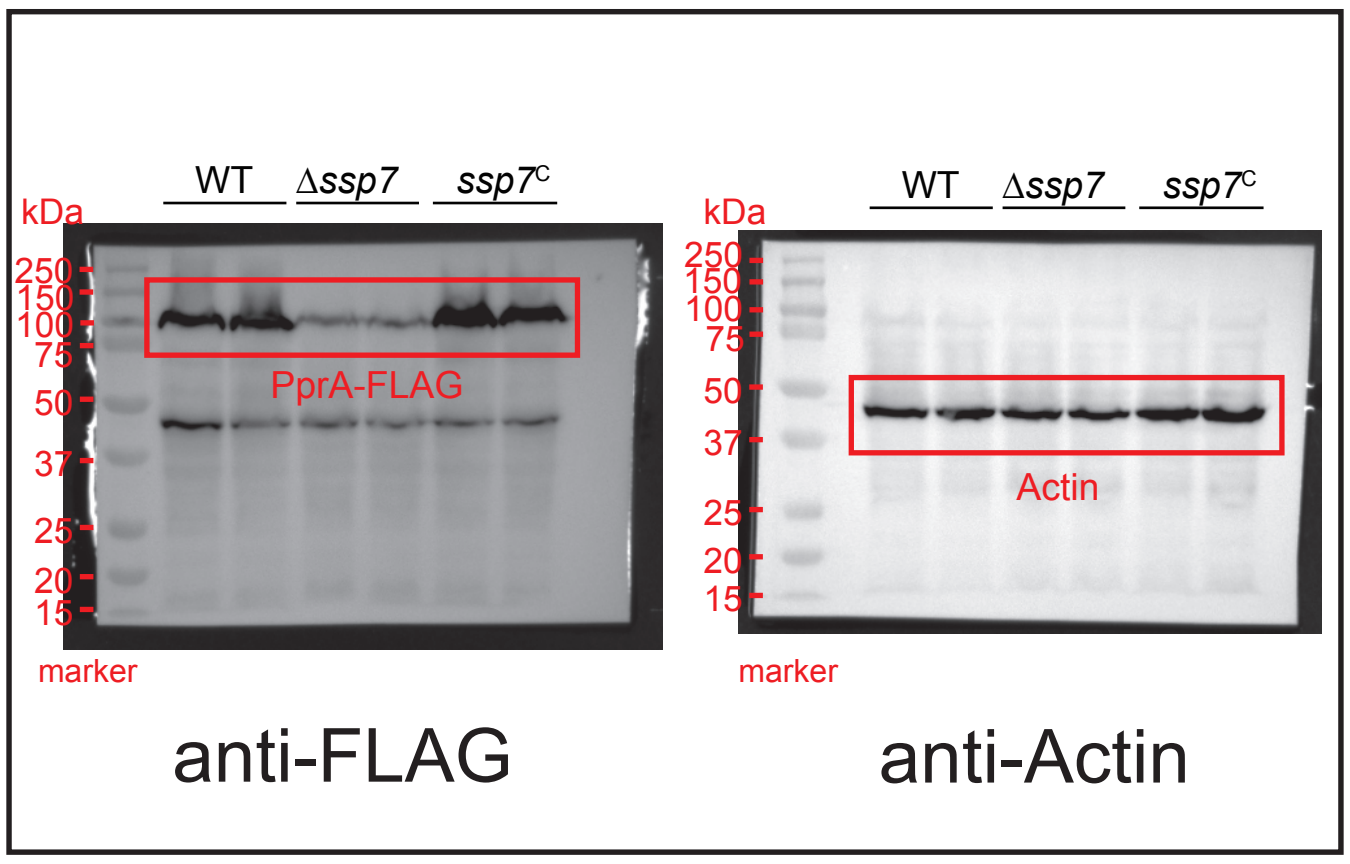

Fig 7C

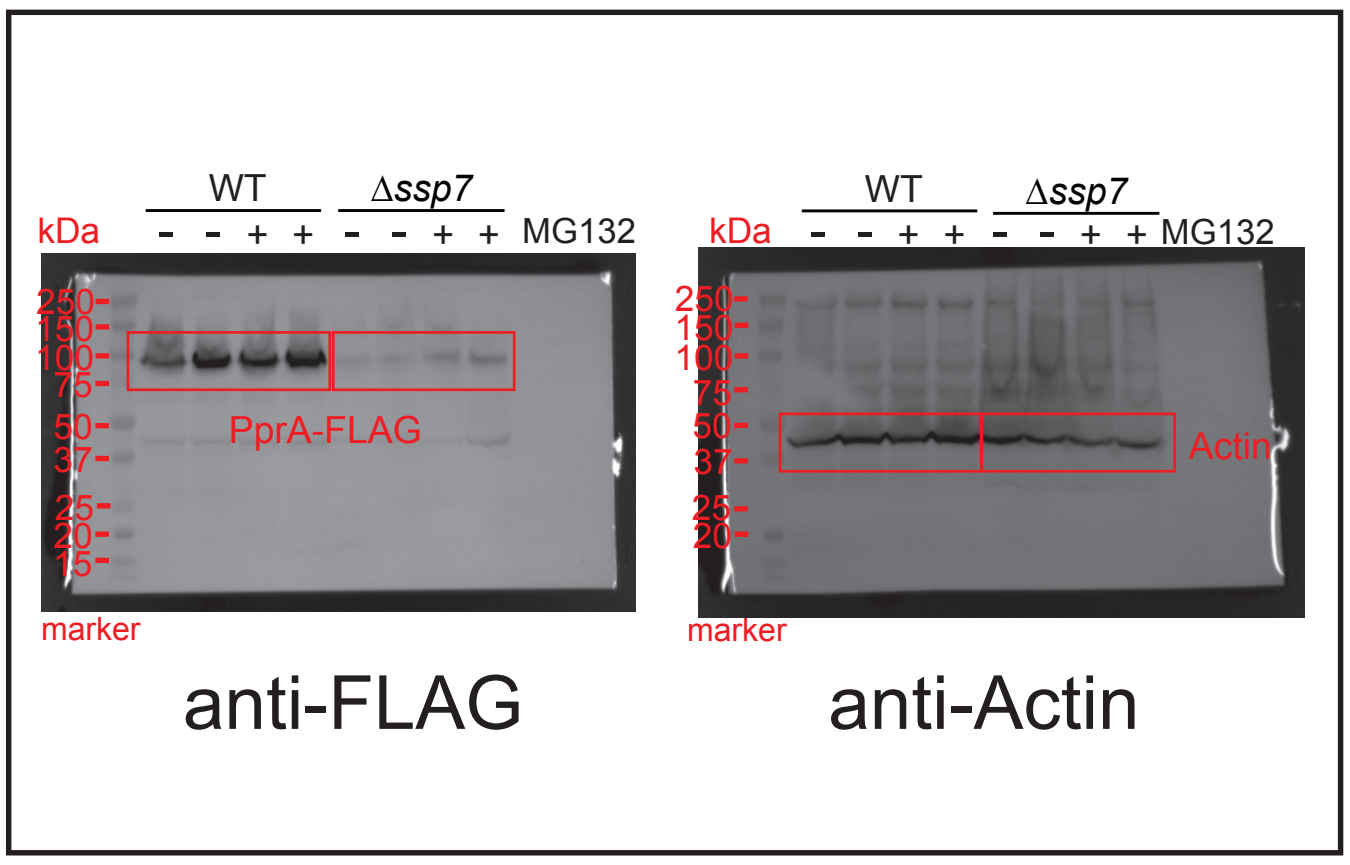

Fig 7E

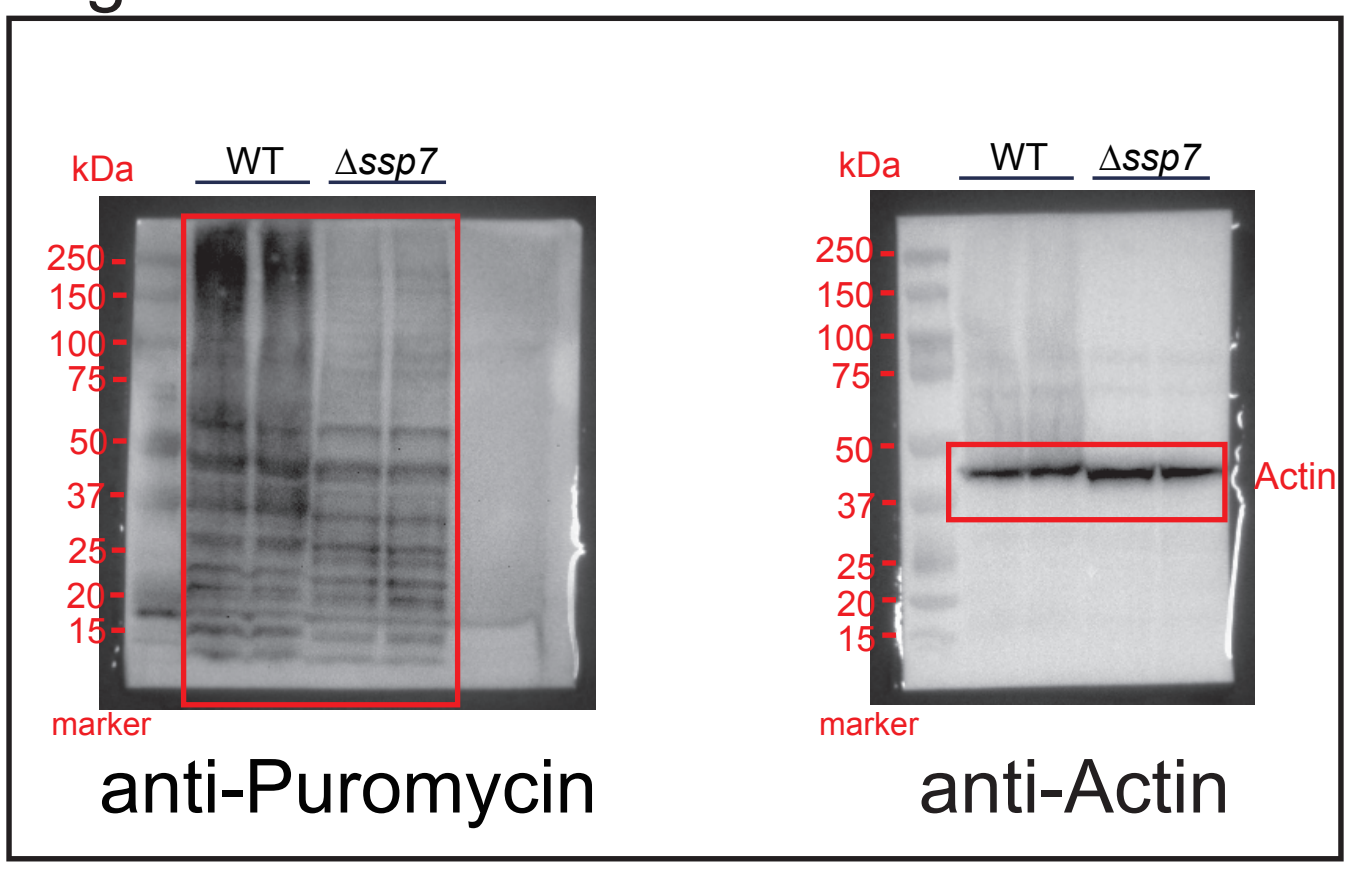

Fig 7J

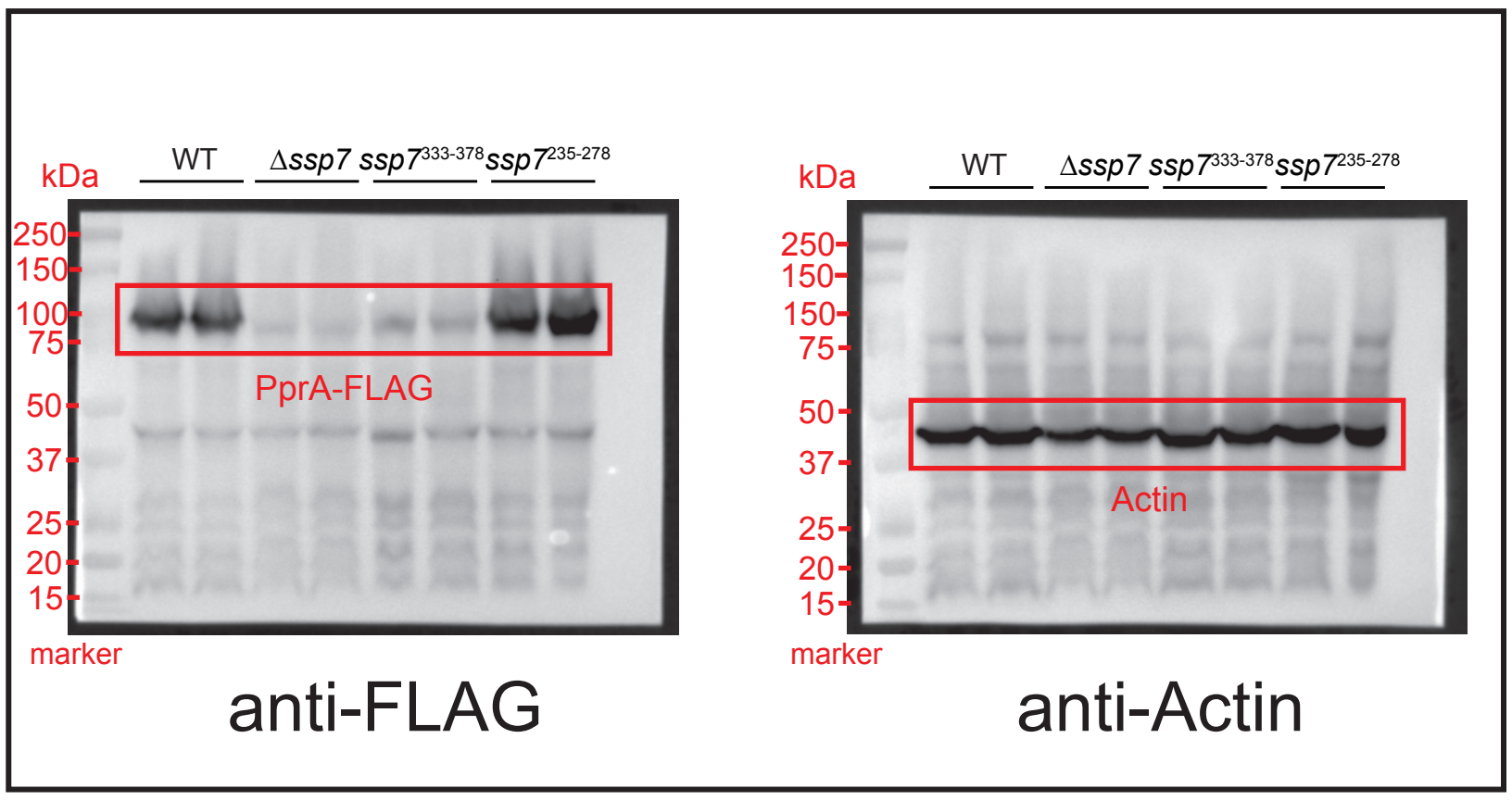

S1B Fig

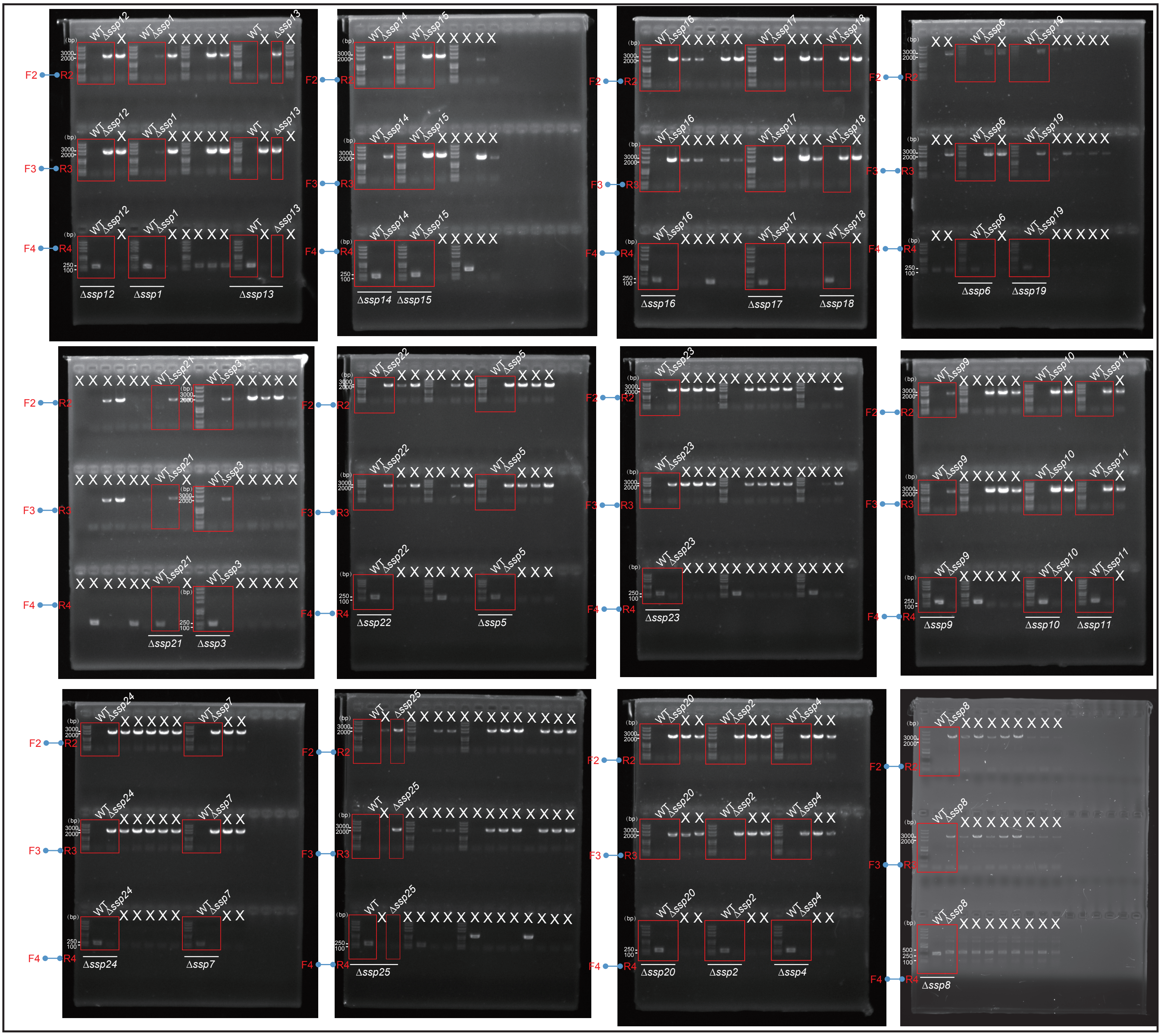

F2
R2
F3
R3
F4
R4
WT
Δssp12
Δssp1
Δssp13
Δssp14
Δssp15
Δssp16
Δssp17
Δssp18
Δssp6
Δssp19
Δssp21
Δssp3
Δssp22
Δssp5
Δssp23
Δssp9
Δssp10
Δssp11
Δssp24
Δssp7
Δssp25
Δssp20
Δssp2
Δssp4
Δssp8
(bp)
3000
2000
250
100
500

S3 Fig

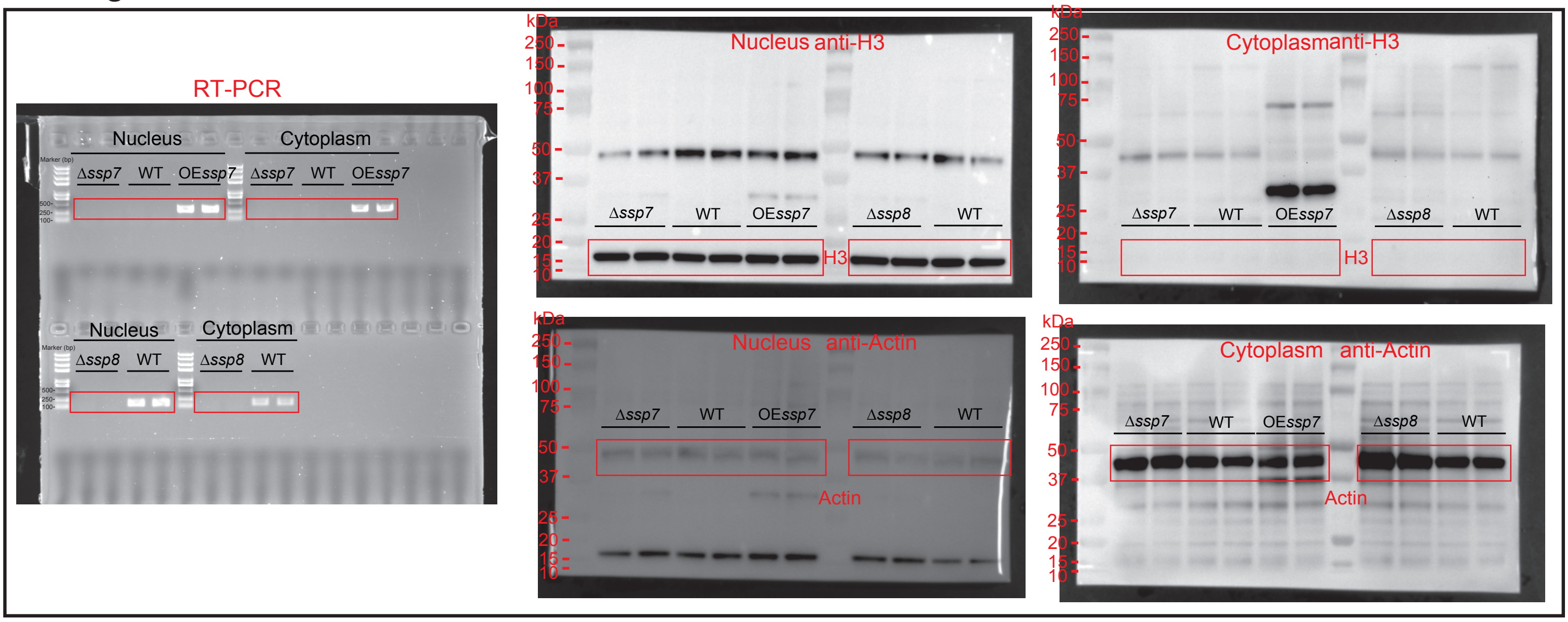

S7B Fig

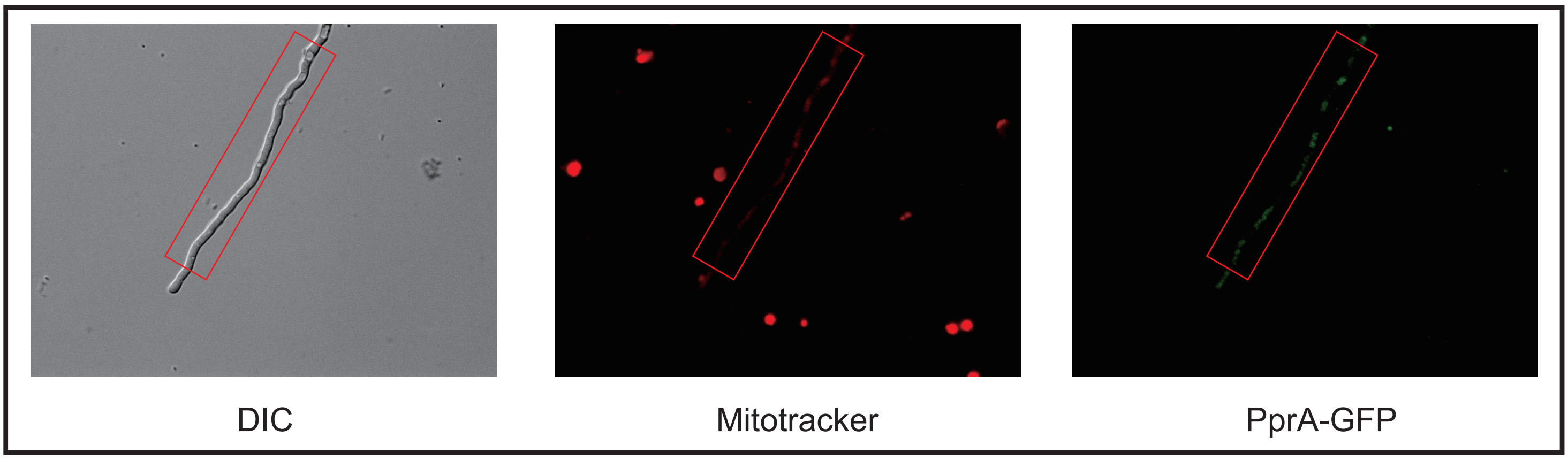

Supplement: Supplementary file 1 [file ppat.1014681.s001.pdf]
