# Supplementary material for: Large-scale genome re-annotation uncovers pathogen-specific lncRNA regulators of fungal growth and virulence
Source: PLoS Pathog. 2026 Oct 9;22(10):e1014681. doi: 10.1371/journal.ppat.1014681 (PMC13652153; doi:10.1371/journal.ppat.1014681)
Supplement: Supplementary file 2 [file ppat.1014681.s010.docx]

**S1 Table.** Summary of automated cDNA predictions and actual cDNA lengths for the 25 *ssp* genes

| **Gene Name** | **Gene ID** | **Automated prediction of cDNA (nt)** | **Actual cDNA (nt)** | **Coding potential** |
| --- | --- | --- | --- | --- |
| *ssp10* | *AFUB_020640* | 2103 | 2103 | Coding gene |
| *ssp11* | *AFUB_022660* | 306 | 306 | Coding gene |
| *ssp12* | *AFUB_022960* | 342 | 342 | Coding gene |
| *ssp16* | *AFUB_030420* | 603 | 603 | Coding gene |
| *ssp1* | *AFUB_025860* | 444 | 547 | Non-coding gene |
| *ssp4* | *AFUB_084809* | 231 | 260 | Non-coding gene |
| *ssp7* | *AFUB_026560* | 204 | 450 | Non-coding gene |
| *ssp8* | *AFUB_059960* | 741 | 915 | Non-coding gene |
| *ssp9* | *AFUB_000670* | 723 | 912/826/862/776 | Non-coding gene |
| *ssp14* | *AFUB_028430* | 579 | 898/835/836/773 | Non-coding gene |
| *ssp17* | *AFUB_037960* | 612 | 742/664 | Non-coding gene |
| *ssp18* | *AFUB_038990* | 315 | 302/362 | Non-coding gene |
| *ssp13* | *AFUB_027390* | 309 | 258 | Uncertain coding potential gene |
| *ssp19* | *AFUB_063670* | 300 | 300 | Uncertain coding potential gene |
| *ssp20* | *AFUB_065260* | 819 | 936 | Uncertain coding potential gene |
| *ssp21* | *AFUB_078190* | 1047 | 1047 | Uncertain coding potential gene |
| *ssp2* | *AFUB_080990* | 822 | NA | NA |
| *ssp3* | *AFUB_081010* | 333 | NA | NA |
| *ssp5* | *AFUB_085000* | 762 | NA | NA |
| *ssp6* | *AFUB_091580* | 513 | NA | NA |
| *ssp15* | *AFUB_030170* | 498 | NA | NA |
| *ssp22* | *AFUB_084250* | 267 | NA | NA |
| *ssp23* | *AFUB_086630* | 582 | NA | NA |
| *ssp24* | *AFUB_087700* | 216 | NA | NA |
| *ssp25* | *AFUB_090750* | 216 | NA | NA |
