# Supplementary material for: Large-scale genome re-annotation uncovers pathogen-specific lncRNA regulators of fungal growth and virulence
Source: PLoS Pathog. 2026 Oct 9;22(10):e1014681. doi: 10.1371/journal.ppat.1014681 (PMC13652153; doi:10.1371/journal.ppat.1014681)
Supplement: Supplementary file 3 [file ppat.1014681.s011.docx]

**S2 Table.** All strains used in this study

| **Strain** | **Genotype** | **Source** |
| --- | --- | --- |
| Δ*ku80* | Δ*ku80* | *A. fumigatus* A1163 Δ*ku80*, wild type |
| Δ*ssp1* | Δ*ku80*, Δ*ssp1*::*hph* | This study |
| Δ*ssp2* | Δ*ku80*, Δ*ssp2*::*hph* | This study |
| Δ*ssp3* | Δ*ku80*, Δ*ssp3*::*hph* | This study |
| Δ*ssp4* | Δ*ku80*, Δ*ssp4*::*hph* | This study |
| Δ*ssp5* | Δ*ku80*, Δ*ssp5*::*hph* | This study |
| Δ*ssp6* | Δ*ku80*, Δ*ssp6*::*hph* | This study |
| Δ*ssp7* | Δ*ku80*, Δ*ssp7*::*hph* | This study |
| Δ*ssp8* | Δ*ku80*, Δ*ssp8*::*hph* | This study |
| Δ*ssp9* | Δ*ku80*, Δ*ssp9*::*hph* | This study |
| Δ*ssp10* | Δ*ku80*, Δ*ssp10*::*hph* | This study |
| Δ*ssp11* | Δ*ku80*, Δ*ssp11*::*hph* | This study |
| Δ*ssp12* | Δ*ku80*, Δ*ssp12*::*hph* | This study |
| Δ*ssp13* | Δ*ku80*, Δ*ssp13*::*hph* | This study |
| Δ*ssp14* | Δ*ku80*, Δ*ssp14*::*hph* | This study |
| Δ*ssp15* | Δ*ku80*, Δ*ssp15*::*hph* | This study |
| Δ*ssp16* | Δ*ku80*, Δ*ssp16*::*hph* | This study |
| Δ*ssp17* | Δ*ku80*, Δ*ssp17*::*hph* | This study |
| Δ*ssp18* | Δ*ku80*, Δ*ssp18*::*hph* | This study |
| Δ*ssp19* | Δ*ku80*, Δ*ssp19*::*hph* | This study |
| Δ*ssp20* | Δ*ku80*, Δ*ssp20*::*hph* | This study |
| Δ*ssp21* | Δ*ku80*, Δ*ssp21*::*hph* | This study |
| Δ*ssp22* | Δ*ku80*, Δ*ssp22*::*hph* | This study |
| Δ*ssp23* | Δ*ku80*, Δ*ssp23*::*hph* | This study |
| Δ*ssp24* | Δ*ku80*, Δ*ssp24*::*hph* | This study |
| Δ*ssp25* | Δ*ku80*, Δ*ssp25*::*hph* | This study |
| Δ*AFUB_055330* | Δ*ku80*, Δ*AFUB_055330*::*hph* | This study |
| Δ*AFUB_003120* | Δ*ku80*, Δ*AFUB_003120*::*hph* | This study |
| Δ*AFUB_014240* | Δ*ku80*, Δ*AFUB_014240*::*hph* | This study |
| Δ*AFUB_024920* | Δ*ku80*, Δ*AFUB_024920*::*hph* | This study |
| Δ*AFUB_031460* | Δ*ku80*, Δ*AFUB_031460*::*hph* | This study |
| Δ*AFUB_032690* | Δ*ku80*, Δ*AFUB_032690*::*hph* | This study |
| Δ*AFUB_033500* | Δ*ku80*, Δ*AFUB_033500*::*hph* | This study |
| Δ*AFUB_034540* | Δ*ku80*, Δ*AFUB_034540*::*hph* | This study |
| Δ*AFUB_037450* | Δ*ku80*, Δ*AFUB_037450*::*hph* | This study |
| Δ*AFUB_040480* | Δ*ku80*, Δ*AFUB_040480*::*hph* | This study |
| Δ*AFUB_044330* | Δ*ku80*, Δ*AFUB_044330*::*hph* | This study |
| Δ*AFUB_046060* | Δ*ku80*, Δ*AFUB_046060*::*hph* | This study |
| Δ*AFUB_046830* | Δ*ku80*, Δ*AFUB_046830*::*hph* | This study |
| Δ*AFUB_047340* | Δ*ku80*, Δ*AFUB_047340*::*hph* | This study |
| Δ*AFUB_047780* | Δ*ku80*, Δ*AFUB_047780*::*hph* | This study |
| Δ*AFUB_049190* | Δ*ku80*, Δ*AFUB_049190*::*hph* | This study |
| Δ*AFUB_052710* | Δ*ku80*, Δ*AFUB_052710*::*hph* | This study |
| Δ*AFUB_058210* | Δ*ku80*, Δ*AFUB_058210*::*hph* | This study |
| Δ*AFUB_065800* | Δ*ku80*, Δ*AFUB_065800*::*hph* | This study |
| Δ*AFUB_066690* | Δ*ku80*, Δ*AFUB_066690*::*hph* | This study |
| Δ*AFUB_071110* | Δ*ku80*, Δ*AFUB_071110*::*hph* | This study |
| Δ*AFUB_073350* | Δ*ku80*, Δ*AFUB_073350*::*hph* | This study |
| Δ*AFUB_075110* | Δ*ku80*, Δ*AFUB_075110*::*hph* | This study |
| Δ*AFUB_076500* | Δ*ku80*, Δ*AFUB_076500*::*hph* | This study |
| Δ*AFUB_077400* | Δ*ku80*, Δ*AFUB_077400*::*hph* | This study |
| Δ*AFUB_077440* | Δ*ku80*, Δ*AFUB_077440*::*hph* | This study |
| Δ*AFUB_086810* | Δ*ku80*, Δ*AFUB_086810*::*hph* | This study |
| Δ*AFUB_087580* | Δ*ku80*, Δ*AFUB_087580*::*hph* | This study |
| Δ*AFUB_087590* | Δ*ku80*, Δ*AFUB_087590*::*hph* | This study |
| Δ*AFUB_089830* | Δ*ku80*, Δ*AFUB_089830*::*hph* | This study |
| Δ*AFUB_093370* | Δ*ku80*, Δ*AFUB_093370*::*hph* | This study |
| Δ*AFUB_094100* | Δ*ku80*, Δ*AFUB_094100*::*hph* | This study |
| Δ*AFUB_099750* | Δ*ku80*, Δ*AFUB_099750*::*hph* | This study |
| SSP7-FLAG | Δ*ku80*, *gpdA(p)*::*ssp7*::FLAG::*hph* | This study |
| SSP8-FLAG | Δ*ku80*, *ssp8*::FLAG::*hph* | This study |
| *ssp7*^C^ | Δ*ku80*, Δ*ssp7*::*hph*, *ssp7*::*BleoR* | This study |
| *ssp7*^T2A^ | Δ*ku80*, Δ*ssp7*::*hph*, *ssp7*^T2A^:: *BleoR* | This study |
| *ssp7*^T108A^ | Δ*ku80*, Δ*ssp7*::*hph*, *ssp7*^T108A^:: *BleoR* | This study |
| *ssp7*^T240A^ | Δ*ku80*, Δ*ssp7*::*hph*, *ssp7*^T240A^:: *BleoR* | This study |
| *ssp7*^T184A^ | Δ*ku80*, Δ*ssp7*::*hph*, *ssp7*^T184A^:: *BleoR* | This study |
| *ssp8*^C^ | Δ*ku80*, Δ*ssp8*::*hph*, *ssp8*::*BleoR* | This study |
| *ssp8*^T2A^ | Δ*ku80*, Δ*ssp8*::*hph*, *ssp8*^T2A^::*BleoR* | This study |
| *ssp8*^T729A^ | Δ*ku80*, Δ*ssp8*::*hph*, *ssp8*^T729A^::*BleoR* | This study |
| *ssp8*^T10A^ | Δ*ku80*, Δ*ssp8*::*hph*, *ssp8*^T10A^::*BleoR* | This study |
| *ssp8*^T145A^ | Δ*ku80*, Δ*ssp8*::*hph*, *ssp8*^T145A^::*BleoR* | This study |
| 293 | - | *A. fumigatus* Af293 |
| 293Δ*ssp7* | *A. fumigatus* Af293, Δ*Afu2g10780*::*hph* | This study |
| 293Δ*ssp7*^C^ | *A. fumigatus* Af293, Δ*Afu2g10780*::*hph*, *Afu2g10780*::*BleoR* | This study |
| Δ*pprA*-1 | Δ*ku80*, *pprA* truncation-1::*hph* | This study |
| Δ*pprA*-2 | Δ*ku80*, *pprA* truncation-2::*hph* | This study |
| *pprA*-1^C^ | Δ*ku80*, *pprA* truncation-1::*hph*, *pprA*::*BleoR* | This study |
| *pprA*-2^C^ | Δ*ku80*, *pprA* truncation-2::*hph*, *pprA*::*BleoR* | This study |
| Δ*ssp7*Δ*pprA*-1 | Δ*ku80*, Δ*ssp7*::*hph*, *pprA* truncation-1::*BleoR* | This study |
| Δ*ssp7*Δ*pprA*-2 | Δ*ku80*, Δ*ssp7*::*hph*, *pprA* truncation -2::*BleoR* | This study |
| PprA-FLAG | Δ*ku80*, *pprA*::FLAG::*hph* | This study |
| PprA-GFP | Δ*ku80*, *pprA*::GFP::*hph* | This study |
| Δ*ssp7_*PprA-FLAG | Δ*ku80*, *pprA*::FLAG::*hph*, Δ*ssp7*::*BleoR* | This study |
| Δ*ssp7_*PprA-GFP | Δ*ku80*, *pprA*::GFP::*hph*, Δ*ssp7*::*BleoR* | This study |
| *ssp7*^C^(PprA-FLAG) | Δ*ku80*, *pprA*::FLAG::*hph*, Δ*ssp7*::*BleoR*, *ssp7*::*ptrA* | This study |
| *ssp7*^235-278^ | Δ*ku80*, Δ*ssp7*::*hph*, *ssp7*^235-278^ scramble:: *BleoR* | This study |
| *ssp7*^333-378^ | Δ*ku80*, Δ*ssp7*::*hph*, *ssp7*^333-378^ scramble:: *BleoR* | This study |
| *ssp7*^235-278^(PprA-FLAG) | Δ*ku80*, Δ*ssp7*::*hph*, *ssp7*^235-278^ scramble:: *BleoR, pprA*::FLAG:: *ptrA* | This study |
| *ssp7*^333-378^(PprA-FLAG) | Δ*ku80*, Δ*ssp7*::*hph*, *ssp7*^333-378^ scramble:: *BleoR, pprA*::FLAG:: *ptrA* | This study |
