# Supplementary material for: Large-scale genome re-annotation uncovers pathogen-specific lncRNA regulators of fungal growth and virulence
Source: PLoS Pathog. 2026 Oct 9;22(10):e1014681. doi: 10.1371/journal.ppat.1014681 (PMC13652153; doi:10.1371/journal.ppat.1014681)
Supplement: Supplementary file 4 [file ppat.1014681.s012.docx]

**S3 Table.** All primers used in this study

| Primer | Sequence 5’-3’ | Comment |
| --- | --- | --- |
| q025860-F | GGAGGCAGCAAGTTGAAGTAATAT | RT-qPCR of *ssp1* in *A. fumigatus* Δ*ku80* |
| q025860-R | GCTTCCAGGCGTTCAGAGTC |  |
| q080990-F | ACACGACCATCAGGGCAACT | RT-qPCR of *ssp2* in *A. fumigatus* Δ*ku80* |
| q080990-R | CATTCACCACAGCGTCCCAC |  |
| q081010-F | GGCAGTTAAACGGACTCCTGG | RT-qPCR of *ssp3* in *A. fumigatus* Δ*ku80* |
| q081010-R | TACTCAACGGTGGCTTGTCGT |  |
| q084809-F | GGCGATGACGATGTGGCA | RT-qPCR of *ssp4* in *A. fumigatus* Δ*ku80* |
| q084809-R | GCAAGGCTCAAGCAAGTAGAAG |  |
| q085000-F | AGAGCCGTCCCAGACCCTA | RT-qPCR of *ssp5* in *A. fumigatus* Δ*ku80* |
| q085000-R | CAGTCACCGCAATCTTTACCC |  |
| q091580-F | ACCTCCTTTGAGCATGGATCTG | RT-qPCR of *ssp6* in *A. fumigatus* Δ*ku80* |
| q091580-R | CACCAGCCACAACGCCTTC |  |
| q026560-F | GGTGCCGCTCAAGGTGTTA | RT-qPCR of *ssp7* in *A. fumigatus* Δ*ku80* |
| q026560-R | TTGAAGCTGGACAGACAGATAGAAA |  |
| q059960-F | CGCCTTTCTTCTTCGATTTCC | RT-qPCR of *ssp8* in *A. fumigatus* Δ*ku80* |
| q059960-R | GCCGACGACGACTGCTTCT |  |
| q000670-F | ATGGCGACTCAACGGAAGG | RT-qPCR of *ssp9* in *A. fumigatus* Δ*ku80* |
| q000670-R | AGAGCTGGCTGCGGTGTCT |  |
| q020640-F | CGCAGTCCACTCCCGCTAT | RT-qPCR of *ssp10* in *A. fumigatus* Δ*ku80* |
| q020640-R | AAGTCCGACGCCGGAACA |  |
| q022660-F | GTCCCGAACCCAGTCCTCA | RT-qPCR of *ssp11* in *A. fumigatus* Δ*ku80* |
| q022660-R | TGTCCCGTCCTCGTATGCTC |  |
| q022960-F | GAGACGACAACAGCACCAACC | RT-qPCR of *ssp12* in *A. fumigatus* Δ*ku80* |
| q022960-R | GCGTGACGAGGGCCTTATT |  |
| q027390-F | AGCGGCTCGGTCCTTACTG | RT-qPCR of *ssp13* in *A. fumigatus* Δ*ku80* |
| q027390-R | GGAGATTGCTGAGCGTGGG |  |
| q028430-F | GCAGGTCACTGCCCTACGAG | RT-qPCR of *ssp14* in *A. fumigatus* Δ*ku80* |
| q028430-R | AAGTCCTCTGCGATTGTTTGGT |  |
| q030170-F | TCGCTGCAACAACATCAACCT | RT-qPCR of *ssp15* in *A. fumigatus* Δ*ku80* |
| q030170-R | GACTCAAGCCACTGGAGGGA |  |
| q030420-F | GAAAGCTCTAAACCCACCACGT | RT-qPCR of *ssp16* in *A. fumigatus* Δ*ku80* |
| q030420-R | CAAACGAGAATCATCAAGCCAAT |  |
| q037960-F | TGGCGTAGGATTGATAGAAGGA | RT-qPCR of *ssp17* in *A. fumigatus* Δ*ku80* |
| q037960-R | AGCAGTGGGCATTGTTAGGG |  |
| q038990-F | CCATTCAGCCTTCTGCGACA | RT-qPCR of *ssp18* in *A. fumigatus* Δ*ku80* |
| q038990-R | TGGAAGAGCGCCAAGGAAA |  |
| q063670-F | GCCTACCTATCACTCCTCCTCCT | RT-qPCR of *ssp19* in *A. fumigatus* Δ*ku80* |
| q063670-R | GGCTTTCTGCTGCTGCGTC |  |
| q065260-F | GAGACTCCCGTTTCTGGTATCCT | RT-qPCR of *ssp20* in *A. fumigatus* Δ*ku80* |
| q065260-R | TCGTTGTCATCTGCTGCTATGC |  |
| q078190-F | GCCCTCACGGCAACTTCA | RT-qPCR of *ssp21* in *A. fumigatus* Δ*ku80* |
| q078190-R | CCTTGGCAGGGTGGGAAAT |  |
| q084250-F | CGAAACTTGTCGAACCACCTT | RT-qPCR of *ssp22* in *A. fumigatus* Δ*ku80* |
| q084250-R | AGCGTACTTGGCGTTATAGCAG |  |
| q086630-F | TCTGAACCTGCCTGGATGAAA | RT-qPCR of *ssp23* in *A. fumigatus* Δ*ku80* |
| q086630-R | CCGACGGAATCGGCTATGT |  |
| q087700-F | CCGAATCTAATTCCAAACCCA | RT-qPCR of *ssp24* in *A. fumigatus* Δ*ku80* |
| q087700-R | CGCCGTTGAATCCATCTTG |  |
| q090750-F | CAACGCTATCCTCCTCTTCTCC | RT-qPCR of *ssp25* in *A. fumigatus* Δ*ku80* |
| q090750-R | TGTCAAGGTCGGTGTTGTCG |  |
| 025860-P2 | ctagtcaaaaggtcgcggtg | Deletion of *ssp1* in *A. fumigatus* Δ*ku80* |
| 025860-P3 | CGGCGGATTTTAGGCTCAAGgcacctttctgacaccatgg |  |
| 025860-P4 | GTTGCCTAGTGAATGCTCCGtatcgccgattttgcctgtg |  |
| 025860-P5 | ttcctggcgagttctgatgt |  |
| 025860hph-F | CTTGAGCCTAAAATCCGCCG |  |
| 025860hph-R | CGGAGCATTCACTAGGCAAC |  |
| 025860-F2 | gggcctcgttgtggatgt | Verification of Δ*ssp1* in *A. fumigatus* Δ*ku80* |
| 025860-R2 | TCACATTCTCCTTCGCTTACTGC |  |
| 025860-F3 | CGAGATCAAGCAGATCAACGG |  |
| 025860-R3 | agtggactcctcgatctcct |  |
| 025860-F4 | GGAGGCAGCAAGTTGAAGTAATAT |  |
| 025860-R4 | GCTTCCAGGCGTTCAGAGTC |  |
| 080990-P2 | gcggggaaagactaggctat | Deletion of *ssp2* in *A. fumigatus* Δ*ku80* |
| 080990-P3 | CGGCGGATTTTAGGCTCAAGagatgaggatggctcgatgg |  |
| 080990-P4 | GTTGCCTAGTGAATGCTCCGcctgcagactgtggaaatgg |  |
| 080990-P5 | cgtgacgagggatgcattag |  |
| 080990hph-F | CTTGAGCCTAAAATCCGCCG |  |
| 080990hph-R | CGGAGCATTCACTAGGCAAC |  |
| 080990-F2 | tcaacccagctgacctacg | Verification of Δ*ssp2* in *A. fumigatus* Δ*ku80* |
| 080990-R2 | TCACATTCTCCTTCGCTTACTGC |  |
| 080990-F3 | CGAGATCAAGCAGATCAACGG |  |
| 080990-R3 | gaaccagaccaaagtagccg |  |
| 080990-F4 | ACACGACCATCAGGGCAACT |  |
| 080990-R4 | CATTCACCACAGCGTCCCAC |  |
| 081010-P2 | cagactgtggaaatggctga | Deletion of *ssp3* in *A. fumigatus* Δ*ku80* |
| 081010-P3 | CGGCGGATTTTAGGCTCAAGtagtttccctggagctggtg |  |
| 081010-P4 | GTTGCCTAGTGAATGCTCCGcagtgcgaaggagatcaagc |  |
| 081010-P5 | ttggcatcggttgaaagacg |  |
| 081010hph-F | CTTGAGCCTAAAATCCGCCG |  |
| 081010hph-R | CGGAGCATTCACTAGGCAAC |  |
| 081010-F2 | tacatcgactactaccgcgg | Verification of Δ*ssp3* in *A. fumigatus* Δ*ku80* |
| 081010-R2 | TCACATTCTCCTTCGCTTACTGC |  |
| 081010-F3 | CGAGATCAAGCAGATCAACGG |  |
| 081010-R3 | tccgatcccatgagaactcg |  |
| 081010-F4 | GGCAGTTAAACGGACTCCTGG |  |
| 081010-R4 | TACTCAACGGTGGCTTGTCGT |  |
| 084809-P2 | gaattgggtcggcagatgtc | Deletion of *ssp4* in *A. fumigatus* Δ*ku80* |
| 084809-P3 | CGGCGGATTTTAGGCTCAAGaaatcccagagctgctccat |  |
| 084809-P4 | GTTGCCTAGTGAATGCTCCGcctcattatccccacctttcg |  |
| 084809-P5 | ggtgcactgctcgatttctc |  |
| 084809hph-F | CTTGAGCCTAAAATCCGCCG |  |
| 084809hph-R | CGGAGCATTCACTAGGCAAC |  |
| 084809-F2 | agtcgctgaattccaccgta | Verification of Δ*ssp4* in *A. fumigatus* Δ*ku80* |
| 084809-R2 | TCACATTCTCCTTCGCTTACTGC |  |
| 084809-F3 | CGAGATCAAGCAGATCAACGG |  |
| 084809-R3 | gggcgtcttactctctctcg |  |
| 084809-F4 | GGCGATGACGATGTGGCA |  |
| 084809-R4 | GCAAGGCTCAAGCAAGTAGAAG |  |
| 085000-P2 | ttgaccctaacccaacccg | Deletion of *ssp5* in *A. fumigatus* Δ*ku80* |
| 085000-P3 | CGGCGGATTTTAGGCTCAAGtgtgtgaggaaccaaggtgt |  |
| 085000-P4 | GTTGCCTAGTGAATGCTCCGtggtatctgcccaggaaagg |  |
| 085000-P5 | aggctggagtgctgaattga |  |
| 085000hph-F | CTTGAGCCTAAAATCCGCCG |  |
| 085000hph-R | CGGAGCATTCACTAGGCAAC |  |
| 085000-F2 | agcaatactcggggccataa | Verification of Δ*ssp5* in *A. fumigatus* Δ*ku80* |
| 085000-R2 | TCACATTCTCCTTCGCTTACTGC |  |
| 085000-F3 | CGAGATCAAGCAGATCAACGG |  |
| 085000-R3 | tcgataccctcaatgacggc |  |
| 085000-F4 | AGAGCCGTCCCAGACCCTA |  |
| 085000-R4 | CAGTCACCGCAATCTTTACCC |  |
| 091580-P2 | GCTTGTGCTTCAGAAAAGAAAAAT | Deletion of *ssp6* in *A. fumigatus* Δ*ku80* |
| 091580-P3 | CGGCGGATTTTAGGCTCAAGTAGACCCATACCACCATGTGAAAC |  |
| 091580-P4 | GTTGCCTAGTGAATGCTCCGGAGATGAGAAATAACTCGTGAAGGA |  |
| 091580hph-F | CTTGAGCCTAAAATCCGCCG |  |
| 091580hph-R | CGGAGCATTCACTAGGCAAC |  |
| 091580-F2 | GGATGTCGTAATCAGAAGAGAAACC | Verification of Δ*ssp6* in *A. fumigatus* Δ*ku80* |
| 091580-R2 | TCACATTCTCCTTCGCTTACTGC |  |
| 091580-F3 | CGAGATCAAGCAGATCAACGG |  |
| 091580-R3 | GGAACCGTCCTTACGTAGTAGGG |  |
| 091580-F4 | ACCTCCTTTGAGCATGGATCTG |  |
| 091580-R4 | CACCAGCCACAACGCCTTC |  |
| 026560-P2 | atctcttgtcaacatgccgc | Deletion of *ssp7* in *A. fumigatus* Δ*ku80* |
| 026560-P3 | CGGCGGATTTTAGGCTCAAGagtcccgtttcttagtcccg |  |
| 026560-P4 | GTTGCCTAGTGAATGCTCCGatgcttcgctgtaccaatgg |  |
| 026560-P5 | ttcgtttgccttccttgacg |  |
| 026560hph-F | CTTGAGCCTAAAATCCGCCG |  |
| 026560hph-R | CGGAGCATTCACTAGGCAAC |  |
| 026560-F2 | aagcgccattggtttcgatt | Verification of Δ*ssp7* in *A. fumigatus* Δ*ku80* |
| 026560-R2 | TCACATTCTCCTTCGCTTACTGC |  |
| 026560-F3 | CGAGATCAAGCAGATCAACGG |  |
| 026560-R3 | aagatacttgtcaagcgccc |  |
| 026560-F4 | GTCTCACAAGGCTGAACCAACA |  |
| 026560-R4 | AAATTACCGAGGATGAGAAGATAGG |  |
| 059960-P2 | AAGGAGCCTAAGCAGCGGA | Deletion of *ssp8* in *A. fumigatus* Δ*ku80* |
| 059960-P3 | CGGCGGATTTTAGGCTCAAGAGTTACAAGCGACAGCACTTGAG |  |
| 059960-P4 | GTTGCCTAGTGAATGCTCCGTATGTTTTCAGGGTGAGGACTGC |  |
| 059960-P5 | GAACGCTGAGGACGTTAGAATG |  |
| 059960hph-F | CTTGAGCCTAAAATCCGCCG |  |
| 059960hph-R | CGGAGCATTCACTAGGCAAC |  |
| 059960-F2 | TCATGTACAGTCAGAAGGCAAGAAC | Verification of Δ*ssp8* in *A. fumigatus* Δ*ku80* |
| 059960-R2 | TCACATTCTCCTTCGCTTACTGC |  |
| 059960-F3 | CGAGATCAAGCAGATCAACGG |  |
| 059960-R3 | TGGAAAAGCAATATGGGCGT |  |
| 059960-F4 | ATGAGTGTATGGACGGGGATG |  |
| 059960-R4 | CTACTGCTCGAAGCGTACCCA |  |
| 000670-P2 | ccgatgaccattgtgagcag | Deletion of *ssp9* in *A. fumigatus* Δ*ku80* |
| 000670-P3 | CGGCGGATTTTAGGCTCAAGcagcgatgttttgattaagcca |  |
| 000670-P4 | GTTGCCTAGTGAATGCTCCGccgtcggagtcaattcagga |  |
| 000670-P5 | gtgtcgctgttggtatgtcc |  |
| 000670hph-F | CTTGAGCCTAAAATCCGCCG |  |
| 000670hph-R | CGGAGCATTCACTAGGCAAC |  |
| 000670-F2 | gccaaggagcattaagagcc | Verification of Δ*ssp9* in *A. fumigatus* Δ*ku80* |
| 000670-R2 | TCACATTCTCCTTCGCTTACTGC |  |
| 000670-F3 | CGAGATCAAGCAGATCAACGG |  |
| 000670-R3 | tctccagtccagcgagattc |  |
| 000670-F4 | ATGGCGACTCAACGGAAGG |  |
| 000670-R4 | AGAGCTGGCTGCGGTGTCT |  |
| 020640-P2 | gattgtcacagtggccctct | Deletion of *ssp10* in *A. fumigatus* Δ*ku80* |
| 020640-P3 | CGGCGGATTTTAGGCTCAAGgggttatgagcggctattcg |  |
| 020640-P4 | GTTGCCTAGTGAATGCTCCGggagccccaagtcataagga |  |
| 020640-P5 | ggcgaaattcaccaccactc |  |
| 020640hph-F | CTTGAGCCTAAAATCCGCCG |  |
| 020640hph-R | CGGAGCATTCACTAGGCAAC |  |
| 020640-F2 | tggcgtctattcgttcctga | Verification of Δ*ssp10* in *A. fumigatus* Δ*ku80* |
| 020640-R2 | TCACATTCTCCTTCGCTTACTGC |  |
| 020640-F3 | CGAGATCAAGCAGATCAACGG |  |
| 020640-R3 | atcgtggattcagtggacac |  |
| 020640-F4 | CGCAGTCCACTCCCGCTAT |  |
| 020640-R4 | AAGTCCGACGCCGGAACA |  |
| 022660-P2 | tcttgcgcagagacttggg | Deletion of *ssp11* in *A. fumigatus* Δ*ku80* |
| 022660-P3 | CGGCGGATTTTAGGCTCAAGatgggtatgcaggacaggaa |  |
| 022660-P4 | GTTGCCTAGTGAATGCTCCGcctggccagtcttcgagatt |  |
| 022660-P5 | acccatactccatccaccac |  |
| 022660hph-F | CTTGAGCCTAAAATCCGCCG |  |
| 022660hph-R | CGGAGCATTCACTAGGCAAC |  |
| 022660-F2 | actgctatctcccggaatcg | Verification of Δ*ssp11* in *A. fumigatus* Δ*ku80* |
| 022660-R2 | TCACATTCTCCTTCGCTTACTGC |  |
| 022660-F3 | CGAGATCAAGCAGATCAACGG |  |
| 022660-R3 | gtatgttgttgcccgttcgt |  |
| 022660-F4 | GTCCCGAACCCAGTCCTCA |  |
| 022660-R4 | TGTCCCGTCCTCGTATGCTC |  |
| 022960-P2 | ccgtcttcaccaagtaccct | Deletion of *ssp12* in *A. fumigatus* Δ*ku80* |
| 022960-P3 | CGGCGGATTTTAGGCTCAAGgagagtaattgcttgcgtcgt |  |
| 022960-P4 | GTTGCCTAGTGAATGCTCCGggttcatatcgttggctcgg |  |
| 022960-P5 | tcgcaaaggaggtggtatca |  |
| 022960hph-F | CTTGAGCCTAAAATCCGCCG |  |
| 022960hph-R | CGGAGCATTCACTAGGCAAC |  |
| 022960-F2 | atcaaggtcgctgctctgat | Verification of Δ*ssp12* in *A. fumigatus* Δ*ku80* |
| 022960-R2 | TCACATTCTCCTTCGCTTACTGC |  |
| 022960-F3 | CGAGATCAAGCAGATCAACGG |  |
| 022960-R3 | agttgaacaagaaacggaccg |  |
| 022960-F4 | GAGACGACAACAGCACCAACC |  |
| 022960-R4 | GCGTGACGAGGGCCTTATT |  |
| 027390-P2 | gacgtgccttgggtctagta | Deletion of *ssp13* in *A. fumigatus* Δ*ku80* |
| 027390-P3 | CGGCGGATTTTAGGCTCAAGtaggtcagttgagtcgcagg |  |
| 027390-P4 | GTTGCCTAGTGAATGCTCCGgcaattgagactgcgggatc |  |
| 027390-P5 | taccatcagcagttctcccg |  |
| 027390hph-F | CTTGAGCCTAAAATCCGCCG |  |
| 027390hph-R | CGGAGCATTCACTAGGCAAC |  |
| 027390-F2 | tcacttattctggccaggca | Verification of Δ*ssp13* in *A. fumigatus* Δ*ku80* |
| 027390-R2 | TCACATTCTCCTTCGCTTACTGC |  |
| 027390-F3 | CGAGATCAAGCAGATCAACGG |  |
| 027390-R3 | ctggtgtgtgttggtggatg |  |
| 027390-F4 | AGCGGCTCGGTCCTTACTG |  |
| 027390-R4 | GGAGATTGCTGAGCGTGGG |  |
| 028430-P2 | atgagcccaggagacaagtc | Deletion of *ssp14* in *A. fumigatus* Δ*ku80* |
| 028430-P3 | CGGCGGATTTTAGGCTCAAGtttctgtacgcaaagtcggc |  |
| 028430-P4 | GTTGCCTAGTGAATGCTCCGgaagcagctgtacaaccgtg |  |
| 028430-P5 | gaggtgtttgttggcgttct |  |
| 028430hph-F | CTTGAGCCTAAAATCCGCCG |  |
| 028430hph-R | CGGAGCATTCACTAGGCAAC |  |
| 028430-F2 | ctcaaacgaaaagaccgcga | Verification of Δ*ssp14* in *A. fumigatus* Δ*ku80* |
| 028430-R2 | TCACATTCTCCTTCGCTTACTGC |  |
| 028430-F3 | CGAGATCAAGCAGATCAACGG |  |
| 028430-R3 | tcgcgttgtcagatgttgtc |  |
| 028430-F4 | GCAGGTCACTGCCCTACGAG |  |
| 028430-R4 | AAGTCCTCTGCGATTGTTTGGT |  |
| 030170-P2 | acggcaccactcaaagtaga | Deletion of *ssp15* in *A. fumigatus* Δ*ku80* |
| 030170-P3 | CGGCGGATTTTAGGCTCAAGgtcggtacagcttatggtcg |  |
| 030170-P4 | GTTGCCTAGTGAATGCTCCGaggtttcgtactcatttaaggga |  |
| 030170-P5 | ctgccccaacaagtacaacc |  |
| 030170hph-F | CTTGAGCCTAAAATCCGCCG |  |
| 030170hph-R | CGGAGCATTCACTAGGCAAC |  |
| 030170-F2 | tccggaaagaaggtcccaaa | Verification of Δ*ssp15* in *A. fumigatus* Δ*ku80* |
| 030170-R2 | TCACATTCTCCTTCGCTTACTGC |  |
| 030170-F3 | CGAGATCAAGCAGATCAACGG |  |
| 030170-R3 | tgccttggtgtacatgtcca |  |
| 030170-F4 | TCGCTGCAACAACATCAACCT |  |
| 030170-R4 | GACTCAAGCCACTGGAGGGA |  |
| 030420-P2 | tcccagttacaacgcatcaa | Deletion of *ssp16* in *A. fumigatus* Δ*ku80* |
| 030420-P3 | CGGCGGATTTTAGGCTCAAGaagttgggttgaagagcacg |  |
| 030420-P4 | GTTGCCTAGTGAATGCTCCGgcggatgttgttaactctaatcg |  |
| 030420-P5 | ccacttcaagccacaagacc |  |
| 030420hph-F | CTTGAGCCTAAAATCCGCCG |  |
| 030420hph-R | CGGAGCATTCACTAGGCAAC |  |
| 030420-F2 | agccagccgtcaaattgtaa | Verification of Δ*ssp16* in *A. fumigatus* Δ*ku80* |
| 030420-R2 | TCACATTCTCCTTCGCTTACTGC |  |
| 030420-F3 | CGAGATCAAGCAGATCAACGG |  |
| 030420-R3 | cacctgatgctacgtctcct |  |
| 030420-F4 | GAAAGCTCTAAACCCACCACGT |  |
| 030420-R4 | CAAACGAGAATCATCAAGCCAAT |  |
| 037960-P2 | ctgcgaactactccgtacct | Deletion of *ssp17* in *A. fumigatus* Δ*ku80* |
| 037960-P3 | CGGCGGATTTTAGGCTCAAGgcagatacagagcagcaagg |  |
| 037960-P4 | GTTGCCTAGTGAATGCTCCGagagcaactggtacgactgg |  |
| 037960-P5 | cttgtcagacgcatcccaag |  |
| 037960hph-F | CTTGAGCCTAAAATCCGCCG |  |
| 037960hph-R | CGGAGCATTCACTAGGCAAC |  |
| 037960-F2 | tgtgttccagattgtgcacg | Verification of Δ*ssp17* in *A. fumigatus* Δ*ku80* |
| 037960-R2 | TCACATTCTCCTTCGCTTACTGC |  |
| 037960-F3 | CGAGATCAAGCAGATCAACGG |  |
| 037960-R3 | ctggacgctctttcagatga |  |
| 037960-F4 | TGGCGTAGGATTGATAGAAGGA |  |
| 037960-R4 | AGCAGTGGGCATTGTTAGGG |  |
| 038990-P2 | gcagtgccattacctcttttc | Deletion of *ssp18* in *A. fumigatus* Δ*ku80* |
| 038990-P3 | CGGCGGATTTTAGGCTCAAGggactgcttggaccttttga |  |
| 038990-P4 | GTTGCCTAGTGAATGCTCCGctgtggaggaagtcagcga |  |
| 038990-P5 | ctctttcggcatgctgactg |  |
| 038990hph-F | CTTGAGCCTAAAATCCGCCG |  |
| 038990hph-R | CGGAGCATTCACTAGGCAAC |  |
| 038990-F2 | tccagcgcatctactcaact | Verification of Δ*ssp18* in *A. fumigatus* Δ*ku80* |
| 038990-R2 | TCACATTCTCCTTCGCTTACTGC |  |
| 038990-F3 | CGAGATCAAGCAGATCAACGG |  |
| 038990-R3 | gctgatcgttcgctctccta |  |
| 038990-F4 | CCATTCAGCCTTCTGCGACA |  |
| 038990-R4 | TGGAAGAGCGCCAAGGAAA |  |
| 063670-P2 | ttgagacttggttccgggg | Deletion of *ssp19* in *A. fumigatus* Δ*ku80* |
| 063670-P3 | CGGCGGATTTTAGGCTCAAGagttgccagccagtctatgt |  |
| 063670-P4 | GTTGCCTAGTGAATGCTCCGgggaggttgtggtggttttc |  |
| 063670-P5 | aggtgctacattccttggtga |  |
| 063670hph-F | CTTGAGCCTAAAATCCGCCG |  |
| 063670hph-R | CGGAGCATTCACTAGGCAAC |  |
| 063670-F2 | gacccgactgattgcaagtc | Verification of Δ*ssp19* in *A. fumigatus* Δ*ku80* |
| 063670-R2 | TCACATTCTCCTTCGCTTACTGC |  |
| 063670-F3 | CGAGATCAAGCAGATCAACGG |  |
| 063670-R3 | aattgattggcacggcatca |  |
| 063670-F4 | GCCTACCTATCACTCCTCCTCCT |  |
| 063670-R4 | GGCTTTCTGCTGCTGCGTC |  |
| 065260-P2 | ccactggcaagcgtgtac | Deletion of *ssp20* in *A. fumigatus* Δ*ku80* |
| 065260-P3 | CGGCGGATTTTAGGCTCAAGactcaagtcagcgcatttgg |  |
| 065260-P4 | GTTGCCTAGTGAATGCTCCGgtccagtggttcatcaggga |  |
| 065260-P5 | gagatgtcgcacagtggaac |  |
| 065260hph-F | CTTGAGCCTAAAATCCGCCG |  |
| 065260hph-R | CGGAGCATTCACTAGGCAAC |  |
| 065260-F2 | agccatcagactcgtcatca | Verification of Δ*ssp20* in *A. fumigatus* Δ*ku80* |
| 065260-R2 | TCACATTCTCCTTCGCTTACTGC |  |
| 065260-F3 | CGAGATCAAGCAGATCAACGG |  |
| 065260-R3 | atacctccctacttgtgcgc |  |
| 065260-F4 | GAGACTCCCGTTTCTGGTATCCT |  |
| 065260-R4 | TCGTTGTCATCTGCTGCTATGC |  |
| 078190-P2 | gaattcgtgatccttccggc | Deletion of *ssp21* in *A. fumigatus* Δ*ku80* |
| 078190-P3 | CGGCGGATTTTAGGCTCAAGgcgaggagaaagaaaacgaga |  |
| 078190-P4 | GTTGCCTAGTGAATGCTCCGatttatcgtgctgccctcct |  |
| 078190-P5 | ggaagagtgctgggtcgtat |  |
| 078190hph-F | CTTGAGCCTAAAATCCGCCG |  |
| 078190hph-R | CGGAGCATTCACTAGGCAAC |  |
| 078190-F2 | agtatagccgccgaagacat | Verification of Δ*ssp21* in *A. fumigatus* Δ*ku80* |
| 078190-R2 | TCACATTCTCCTTCGCTTACTGC |  |
| 078190-F3 | CGAGATCAAGCAGATCAACGG |  |
| 078190-R3 | cgtcaaggcgaatctcagga |  |
| 078190-F4 | GCCCTCACGGCAACTTCA |  |
| 078190-R4 | CCTTGGCAGGGTGGGAAAT |  |
| 084250-P2 | tgtgtgaatgctgcatctct | Deletion of *ssp22* in *A. fumigatus* Δ*ku80* |
| 084250-P3 | CGGCGGATTTTAGGCTCAAGgcgttcttctgcgttcttct |  |
| 084250-P4 | GTTGCCTAGTGAATGCTCCGtgtattgagcaggagggtga |  |
| 084250-P5 | tggttgggtcggaaggattt |  |
| 084250hph-F | CTTGAGCCTAAAATCCGCCG |  |
| 084250hph-R | CGGAGCATTCACTAGGCAAC |  |
| 084250-F2 | cctcgggtttctgccttaga | Verification of Δ*ssp22* in *A. fumigatus* Δ*ku80* |
| 084250-R2 | TCACATTCTCCTTCGCTTACTGC |  |
| 084250-F3 | CGAGATCAAGCAGATCAACGG |  |
| 084250-R3 | tcgaggttccactggtcatc |  |
| 084250-F4 | CGAAACTTGTCGAACCACCTT |  |
| 084250-R4 | AGCGTACTTGGCGTTATAGCAG |  |
| 086630-P2 | cttcgggtatatggccatgg | Deletion of *ssp23* in *A. fumigatus* Δ*ku80* |
| 086630-P3 | CGGCGGATTTTAGGCTCAAGatgcagggaaagtagcctct |  |
| 086630-P4 | GTTGCCTAGTGAATGCTCCGtgttggaaccgctatttggg |  |
| 086630-P5 | tcccaaagatagtccgatagct |  |
| 086630hph-F | CTTGAGCCTAAAATCCGCCG |  |
| 086630hph-R | CGGAGCATTCACTAGGCAAC |  |
| 086630-F2 | cctaggcccattgtttcagg | Verification of Δ*ssp23* in *A. fumigatus* Δ*ku80* |
| 086630-R2 | TCACATTCTCCTTCGCTTACTGC |  |
| 086630-F3 | CGAGATCAAGCAGATCAACGG |  |
| 086630-R3 | ggggcatcaaccataaagca |  |
| 086630-F4 | TCTGAACCTGCCTGGATGAAA |  |
| 086630-R4 | CCGACGGAATCGGCTATGT |  |
| 087700-P2 | ctcctgtgcagccctagaat | Deletion of *ssp24* in *A. fumigatus* Δ*ku80* |
| 087700-P3 | CGGCGGATTTTAGGCTCAAGtgaggcgaggcgtttatatct |  |
| 087700-P4 | GTTGCCTAGTGAATGCTCCGgtgtggttggctgatgactg |  |
| 087700-P5 | tcttaccgacgacatgcagt |  |
| 087700hph-F | CTTGAGCCTAAAATCCGCCG |  |
| 087700hph-R | CGGAGCATTCACTAGGCAAC |  |
| 087700-F2 | atccaactccacagccacaa | Verification of Δ*ssp24* in *A. fumigatus* Δ*ku80* |
| 087700-R2 | TCACATTCTCCTTCGCTTACTGC |  |
| 087700-F3 | CGAGATCAAGCAGATCAACGG |  |
| 087700-R3 | ccatcacagggtcgtctttt |  |
| 087700-F4 | CCGAATCTAATTCCAAACCCA |  |
| 087700-R4 | CGCCGTTGAATCCATCTTG |  |
| 090750-P2 | cattgctttaccatgcgacg | Deletion of *ssp25* in *A. fumigatus* Δ*ku80* |
| 090750-P3 | CGGCGGATTTTAGGCTCAAGatggagtggttgtagtgcgt |  |
| 090750-P4 | GTTGCCTAGTGAATGCTCCGagctctacagatgcaggact |  |
| 090750-P5 | cctcctctctcggaccattg |  |
| 090750hph-F | CTTGAGCCTAAAATCCGCCG |  |
| 090750hph-R | CGGAGCATTCACTAGGCAAC |  |
| 090750-F2 | acccctagtatggcctcaaa | Verification of Δ*ssp25* in *A. fumigatus* Δ*ku80* |
| 090750-R2 | TCACATTCTCCTTCGCTTACTGC |  |
| 090750-F3 | CGAGATCAAGCAGATCAACGG |  |
| 090750-R3 | tcatagaagtggcctagggg |  |
| 090750-F4 | CAACGCTATCCTCCTCTTCTCC |  |
| 090750-R4 | TGTCAAGGTCGGTGTTGTCG |  |
| 055330-P2 | GGTCGCTGTAACCAAAGCTCTC | Deletion of *AFUB_055330* in *A. fumigatus* Δ*ku80* |
| 055330-P3 | CGGCGGATTTTAGGCTCAAGGGCGACAATTTTCAGTGAGG |  |
| 055330-P4 | GTTGCCTAGTGAATGCTCCGATCTGTTCAGTGGTGTCGTGG |  |
| 055330-P5 | CGCTAAGTTGGCTTCTAACAATAAT |  |
| 055330hph-F | CTTGAGCCTAAAATCCGCCG |  |
| 055330hph-R | CGGAGCATTCACTAGGCAAC |  |
| 055330-F2 | AGATGGTCGAGGTGTAGTCGC | Verification of Δ*AFUB_055330* in *A. fumigatus* Δ*ku80* |
| 055330-R2 | TCACATTCTCCTTCGCTTACTGC |  |
| 055330-F3 | CGAGATCAAGCAGATCAACGG |  |
| 055330-R3 | TTGTGACGTTGAGGGCAATTAC |  |
| 055330-F4 | ATGATTGCTTACGGTGGTGTCA |  |
| 055330-R4 | CGTCGCCGAAGTTGTTGG |  |
| 003120-P2 | ACGCTGTCACGGTAGCAAACA | Deletion of *AFUB_003120* in *A. fumigatus* Δ*ku80* |
| 003120-P3 | CGGCGGATTTTAGGCTCAAGTGATATATGCACGCAACACGAAG |  |
| 003120-P4 | GTTGCCTAGTGAATGCTCCGCGGGAGGGTAACTTGTTTTGG |  |
| 003120-P5 | TCTGCAATCAATATACTCCAGCACT |  |
| 003120hph-F | CTTGAGCCTAAAATCCGCCG |  |
| 003120hph-R | CGGAGCATTCACTAGGCAAC |  |
| 003120-F2 | AGATGTCGGCAGAAACAATCG | Verification of Δ*AFUB_003120* in *A. fumigatus* Δ*ku80* |
| 003120-R2 | TCACATTCTCCTTCGCTTACTGC |  |
| 003120-F3 | CGAGATCAAGCAGATCAACGG |  |
| 003120-R3 | GTTGTCGCCATGCCAATGT |  |
| 003120-F4 | TTTGCTGCTGCCATCATCTC |  |
| 003120-R4 | CCAAGCTCACCCGCAATCT |  |
| 014240-P2 | ACCTGGGATTAAGAAGTCTCGC | Deletion of *AFUB_014240* in *A. fumigatus* Δ*ku80* |
| 014240-P3 | CGGCGGATTTTAGGCTCAAGAGCATTACATTCAACTCTGCATTGT |  |
| 014240-P4 | GTTGCCTAGTGAATGCTCCGCGACTCAACAATGGATTAAATGATG |  |
| 014240-P5 | CGAAAGCTTGTCTATGAATCACAA |  |
| 014240hph-F | CTTGAGCCTAAAATCCGCCG |  |
| 014240hph-R | CGGAGCATTCACTAGGCAAC |  |
| 014240-F2 | AGCGTGATGGGGTCCAGTCT | Verification of Δ*AFUB_014240* in *A. fumigatus* Δ*ku80* |
| 014240-R2 | TCACATTCTCCTTCGCTTACTGC |  |
| 014240-F3 | CGAGATCAAGCAGATCAACGG |  |
| 014240-R3 | CCTAGGCTGAAAAGAACCTTGG |  |
| 014240-F4 | GCCTCGCCAGTTCCAAGTT |  |
| 014240-R4 | TGTAGGGCTCTTTCTTCCTTCTG |  |
| 024920-P2 | GGCAGAGATGTATCGCAAGCA | Deletion of *AFUB_024920* in *A. fumigatus* Δ*ku80* |
| 024920-P3 | CGGCGGATTTTAGGCTCAAGGACTCGGATCTCAATCAAACAAAG |  |
| 024920-P4 | GTTGCCTAGTGAATGCTCCGAGGAATGCTACCTTAACCTAGTGGA |  |
| 024920-P5 | GTAGACATCAGGAAGCCCGC |  |
| 024920hph-F | CTTGAGCCTAAAATCCGCCG |  |
| 024920hph-R | CGGAGCATTCACTAGGCAAC |  |
| 024920-F2 | GGCGACACAAATGATCTGGAA | Verification of Δ*AFUB_024920* in *A. fumigatus* Δ*ku80* |
| 024920-R2 | TCACATTCTCCTTCGCTTACTGC |  |
| 024920-F3 | CGAGATCAAGCAGATCAACGG |  |
| 024920-R3 | CAATATCACCGAATTGAAGCTGAT |  |
| 024920-F4 | TCTTACACCTGAGTGAGTGGGATT |  |
| 024920-R4 | ATGGGACGGGCTGTTTCTG |  |
| 031460-P2 | TTCTCTACCTATGCTTCCATTGTTG | Deletion of *AFUB_031460* in *A. fumigatus* Δ*ku80* |
| 031460-P3 | CGGCGGATTTTAGGCTCAAGCTACAGCATCAAAGGCTAACACTCA |  |
| 031460-P4 | GTTGCCTAGTGAATGCTCCGCCTCTTATTTGGTAATTCGGCTG |  |
| 031460-P5 | AAATGCCGAACCCATCTCAGT |  |
| 031460hph-F | CTTGAGCCTAAAATCCGCCG |  |
| 031460hph-R | CGGAGCATTCACTAGGCAAC |  |
| 031460-F2 | CTCAACCGAAGTTTCTTTTACGAG | Verification of Δ*AFUB_031460* in *A. fumigatus* Δ*ku80* |
| 031460-R2 | TCACATTCTCCTTCGCTTACTGC |  |
| 031460-F3 | CGAGATCAAGCAGATCAACGG |  |
| 031460-R3 | GCACACATAAGGTGGTCGTCA |  |
| 031460-F4 | CTAACAGACCTCCCAAGCAGAGT |  |
| 031460-R4 | GTTGTGGAAGGCTGAGACGG |  |
| 032690-P2 | CAGAGATTTCTGATCCTCGGATT | Deletion of *AFUB_032690* in *A. fumigatus* Δ*ku80* |
| 032690-P3 | CGGCGGATTTTAGGCTCAAGGGTCATAAAGGTCGTAACAGTCGTA |  |
| 032690-P4 | GTTGCCTAGTGAATGCTCCGTCTCTAACTCTCGCAAGTCTCTCAC |  |
| 032690-P5 | ACTGGATGCTGATGCCTGCT |  |
| 032690hph-F | CTTGAGCCTAAAATCCGCCG |  |
| 032690hph-R | CGGAGCATTCACTAGGCAAC |  |
| 032690-F2 | CATTCTTCGTTTAGCCCGCA | Verification of Δ*AFUB_032690* in *A. fumigatus* Δ*ku80* |
| 032690-R2 | TCACATTCTCCTTCGCTTACTGC |  |
| 032690-F3 | CGAGATCAAGCAGATCAACGG |  |
| 032690-R3 | GGAGAAGAGCCAGTCAGTAGCAG |  |
| 032690-F4 | GTCTCCGAGCCATCCAAGC |  |
| 032690-R4 | CTCCGTAACAGCATCATCAGGT |  |
| 033500-P2 | ATGTTCCCCTTGCATCTTATGAG | Deletion of *AFUB_033500* in *A. fumigatus* Δ*ku80* |
| 033500-P3 | CGGCGGATTTTAGGCTCAAGCTACAAGCCAGGAAGGAAAGGA |  |
| 033500-P4 | GTTGCCTAGTGAATGCTCCGTATGCATTACTTGCCTAGTACCAAT |  |
| 033500-P5 | ATCCTCTTCATCCTCGTCCACT |  |
| 033500hph-F | CTTGAGCCTAAAATCCGCCG |  |
| 033500hph-R | CGGAGCATTCACTAGGCAAC |  |
| 033500-F2 | GTAGCGAGTGTAACAGCGATTGAC | Verification of Δ*AFUB_033500* in *A. fumigatus* Δ*ku80* |
| 033500-R2 | TCACATTCTCCTTCGCTTACTGC |  |
| 033500-F3 | CGAGATCAAGCAGATCAACGG |  |
| 033500-R3 | TGATGGGAGAAGCATCAAATTTC |  |
| 033500-F4 | TCTATCCTGCTGCCGAGAAACT |  |
| 033500-R4 | GGGTGACGCTGTCTGAAACG |  |
| 034540-P2 | GAGGGCAGCATCACCGTC | Deletion of *AFUB_034540* in *A. fumigatus* Δ*ku80* |
| 034540-P3 | CGGCGGATTTTAGGCTCAAGCTTGCCAAATGTGCCGATAG |  |
| 034540-P4 | GTTGCCTAGTGAATGCTCCGATGGCAGTAGACTTTGTTTGATTGA |  |
| 034540-P5 | CTGGACGCCTTTCGAGAGG |  |
| 034540hph-F | CTTGAGCCTAAAATCCGCCG |  |
| 034540hph-R | CGGAGCATTCACTAGGCAAC |  |
| 034540-F2 | CCGAAAGGTCGATCTGCATC | Verification of Δ*AFUB_034540* in *A. fumigatus* Δ*ku80* |
| 034540-R2 | TCACATTCTCCTTCGCTTACTGC |  |
| 034540-F3 | CGAGATCAAGCAGATCAACGG |  |
| 034540-R3 | ACGCCGATAGGAGATGGGAT |  |
| 034540-F4 | AGTCGGGACATCTCGGTGG |  |
| 034540-R4 | ATGGTCGTGAAGTTGTTCAGATACA |  |
| 037450-P2 | GGCACGCTTAGCTGCATTTG | Deletion of *AFUB_037450* in *A. fumigatus* Δ*ku80* |
| 037450-P3 | CGGCGGATTTTAGGCTCAAGGTTGTTGATGTATCTCCAAAACGG |  |
| 037450-P4 | GTTGCCTAGTGAATGCTCCGGGTCAAGGCGTTCTCATCATTT |  |
| 037450-P5 | CAAATGCCAAGCCTCCCTATC |  |
| 037450hph-F | CTTGAGCCTAAAATCCGCCG |  |
| 037450hph-R | CGGAGCATTCACTAGGCAAC |  |
| 037450-F2 | CGAATGGCGGTAATAGCATAGAG | Verification of Δ*AFUB_037450* in *A. fumigatus* Δ*ku80* |
| 037450-R2 | TCACATTCTCCTTCGCTTACTGC |  |
| 037450-F3 | CGAGATCAAGCAGATCAACGG |  |
| 037450-R3 | CAACGAGAAGTACGGTATTGAGGTA |  |
| 037450-F4 | TGAGCCAGCAGCAGGACAG |  |
| 037450-R4 | GGAATCGGTACAGGAACAGGGA |  |
| 040480-P2 | AAATGGCTCAAGTTTGTCTAGGC | Deletion of *AFUB_040480* in *A. fumigatus* Δ*ku80* |
| 040480-P3 | CGGCGGATTTTAGGCTCAAGAAATGATAAAGGGGATTACAGCG |  |
| 040480-P4 | GTTGCCTAGTGAATGCTCCGCGTTGATCCCTCGAGTCTTTCT |  |
| 040480-P5 | ATATGGAGAGGGGAGTGAATGC |  |
| 040480hph-F | CTTGAGCCTAAAATCCGCCG |  |
| 040480hph-R | CGGAGCATTCACTAGGCAAC |  |
| 040480-F2 | CTTGTCTACTAGTTGTGCGCCCT | Verification of Δ*AFUB_040480* in *A. fumigatus* Δ*ku80* |
| 040480-R2 | TCACATTCTCCTTCGCTTACTGC |  |
| 040480-F3 | CGAGATCAAGCAGATCAACGG |  |
| 040480-R3 | TTGCCCCAAGATTCGAACAC |  |
| 040480-F4 | TCGCAGTTCGCTAATACCTCCT |  |
| 040480-R4 | AGTACGGGCGCAGCTTGA |  |
| 044330-P2 | TGACAGATAAGCGGATTCAACAC | Deletion of *AFUB_044330* in *A. fumigatus* Δ*ku80* |
| 044330-P3 | CGGCGGATTTTAGGCTCAAGGGAGGTGGAAAGCCGGTATA |  |
| 044330-P4 | GTTGCCTAGTGAATGCTCCGAATCAGCAAAGCATCTCCTTATCA |  |
| 044330-P5 | GGACACAGCATCAGCATCTCG |  |
| 044330hph-F | CTTGAGCCTAAAATCCGCCG |  |
| 044330hph-R | CGGAGCATTCACTAGGCAAC |  |
| 044330-F2 | CCAACAACGACTGGGACACATA | Verification of Δ*AFUB_044330* in *A. fumigatus* Δ*ku80* |
| 044330-R2 | TCACATTCTCCTTCGCTTACTGC |  |
| 044330-F3 | CGAGATCAAGCAGATCAACGG |  |
| 044330-R3 | GACATCCCAGCAATCTATGAGTGA |  |
| 044330-F4 | ACTGGTGATGCAGGCAAGATG |  |
| 044330-R4 | TTGACCTGGGCAAGTGGAAT |  |
| 046060-P2 | CATTAGGAAACTCTTGGCTCTGAT | Deletion of *AFUB_046060* in *A. fumigatus* Δ*ku80* |
| 046060-P3 | CGGCGGATTTTAGGCTCAAGTCTCCGTTATGCGGTGACAG |  |
| 046060-P4 | GTTGCCTAGTGAATGCTCCGGGGTAGGAGAATGGTGAAAATGT |  |
| 046060-P5 | CAGGTTCTGTCAATCTGGTAAGCT |  |
| 046060hph-F | CTTGAGCCTAAAATCCGCCG |  |
| 046060hph-R | CGGAGCATTCACTAGGCAAC |  |
| 046060-F2 | GACTATGCGATTCGGTCCCT | Verification of Δ*AFUB_046060* in *A. fumigatus* Δ*ku80* |
| 046060-R2 | TCACATTCTCCTTCGCTTACTGC |  |
| 046060-F3 | CGAGATCAAGCAGATCAACGG |  |
| 046060-R3 | CAGTCAAATTCGTGTAGACATAGGC |  |
| 046060-F4 | ACTGCTCTGGGCTATGTCTGG |  |
| 046060-R4 | CGGATGGCGTCAATAACGA |  |
| 046830-P2 | TGTCGGCGTACAAGTTGATTG | Deletion of *AFUB_046830* in *A. fumigatus* Δ*ku80* |
| 046830-P3 | CGGCGGATTTTAGGCTCAAGCGAAGAGTCGCTCTTATTATGGG |  |
| 046830-P4 | GTTGCCTAGTGAATGCTCCGCGACCAGTATATATTGACAGGCATC |  |
| 046830-P5 | CCGTTCCACTGCTGATAAACAGA |  |
| 046830hph-F | CTTGAGCCTAAAATCCGCCG |  |
| 046830hph-R | CGGAGCATTCACTAGGCAAC |  |
| 046830-F2 | GGGTTCGGGGAATCTCACTT | Verification of Δ*AFUB_046830* in *A. fumigatus* Δ*ku80* |
| 046830-R2 | TCACATTCTCCTTCGCTTACTGC |  |
| 046830-F3 | CGAGATCAAGCAGATCAACGG |  |
| 046830-R3 | AGGATATTAAAGAAAAGCCCGTAGT |  |
| 046830-F4 | TCCAGACAGACACCACCGTTG |  |
| 046830-R4 | GCCCGAGACGATGATTTCCT |  |
| 047340-P2 | TGCCATCACAGATAATACCAACACT | Deletion of *AFUB_047340* in *A. fumigatus* Δ*ku80* |
| 047340-P3 | CGGCGGATTTTAGGCTCAAGCGACAGGGACAATCTGAAAAAGTAG |  |
| 047340-P4 | GTTGCCTAGTGAATGCTCCGTTGGGGTGAGAATTAGAGGTGAG |  |
| 047340-P5 | CCGACAGTGTAAAAATAACCACATC |  |
| 047340hph-F | CTTGAGCCTAAAATCCGCCG |  |
| 047340hph-R | CGGAGCATTCACTAGGCAAC |  |
| 047340-F2 | AGGAACCAAATGGCACCCC | Verification of Δ*AFUB_047340* in *A. fumigatus* Δ*ku80* |
| 047340-R2 | TCACATTCTCCTTCGCTTACTGC |  |
| 047340-F3 | CGAGATCAAGCAGATCAACGG |  |
| 047340-R3 | GGTTGTGTTGGAAGCGATGA |  |
| 047340-F4 | TCCGCCGAGAATGGTAAATC |  |
| 047340-R4 | CGTGGTGGAGGTAGAAAGCAG |  |
| 047780-P2 | CTAATCTTCCCTCGTCCACAGTT | Deletion of *AFUB_047780* in *A. fumigatus* Δ*ku80* |
| 047780-P3 | CGGCGGATTTTAGGCTCAAGAAATGCGACCGCTGGAGAG |  |
| 047780-P4 | GTTGCCTAGTGAATGCTCCGCGTGTGAAGTAGTCTGTGTGAAGC |  |
| 047780-P5 | CGCTGAAGATCACAGAGATCACA |  |
| 047780hph-F | CTTGAGCCTAAAATCCGCCG |  |
| 047780hph-R | CGGAGCATTCACTAGGCAAC |  |
| 047780-F2 | CATCCCGCACAGGAACGAG | Verification of Δ*AFUB_047780* in *A. fumigatus* Δ*ku80* |
| 047780-R2 | TCACATTCTCCTTCGCTTACTGC |  |
| 047780-F3 | CGAGATCAAGCAGATCAACGG |  |
| 047780-R3 | TGTAAGTCACCGTCACAACAAAGTC |  |
| 047780-F4 | TTCGATGGCGGGAACTCTG |  |
| 047780-R4 | TCATCTGGATCAGCGCCTGT |  |
| 049190-P2 | ATGCTGGAGACTCTGACATCGTT | Deletion of *AFUB_049190* in *A. fumigatus* Δ*ku80* |
| 049190-P3 | CGGCGGATTTTAGGCTCAAGGGGTCGCTCTGGATATACAGTCA |  |
| 049190-P4 | GTTGCCTAGTGAATGCTCCGCGAGGCAAGCAAATTGGAAT |  |
| 049190-P5 | AGTTTGAGGGACTTATTCTTGTGGT |  |
| 049190hph-F | CTTGAGCCTAAAATCCGCCG |  |
| 049190hph-R | CGGAGCATTCACTAGGCAAC |  |
| 049190-F2 | GGCAGTGCGGTATTGTTACTG | Verification of Δ*AFUB_049190* in *A. fumigatus* Δ*ku80* |
| 049190-R2 | TCACATTCTCCTTCGCTTACTGC |  |
| 049190-F3 | CGAGATCAAGCAGATCAACGG |  |
| 049190-R3 | GGTTTTGAAGGTGATTTGATCTGA |  |
| 049190-F4 | CAGGGCATCGTCCGCTTAT |  |
| 049190-R4 | GGCTGCTCTTTGTGGTACTCTTC |  |
| 052710-P2 | TGAAGCCCCCCAAGAAGAC | Deletion of *AFUB_052710* in *A. fumigatus* Δ*ku80* |
| 052710-P3 | CGGCGGATTTTAGGCTCAAGCAGAAGAATCGATCGTGATCTAATT |  |
| 052710-P4 | GTTGCCTAGTGAATGCTCCGGATCGCCGAAAAAACTTTACATT |  |
| 052710-P5 | TGGACAGTAGAGTTCCGTCATCTG |  |
| 052710hph-F | CTTGAGCCTAAAATCCGCCG |  |
| 052710hph-R | CGGAGCATTCACTAGGCAAC |  |
| 052710-F2 | CTAACCTACAAGAAATGGGGAAGG | Verification of Δ*AFUB_052710* in *A. fumigatus* Δ*ku80* |
| 052710-R2 | TCACATTCTCCTTCGCTTACTGC |  |
| 052710-F3 | CGAGATCAAGCAGATCAACGG |  |
| 052710-R3 | GAAGGAACGCTCGGTTTGCT |  |
| 052710-F4 | CCTCAACAACCGCCACAGAA |  |
| 052710-R4 | CGCATTACCCTGAAGCTCAAT |  |
| 058210-P2 | GCGAGTACAACAGGCGGACT | Deletion of *AFUB_058210* in *A. fumigatus* Δ*ku80* |
| 058210-P3 | CGGCGGATTTTAGGCTCAAGTTCGAAGCGGGAAATAGGAGT |  |
| 058210-P4 | GTTGCCTAGTGAATGCTCCGTATTGTCTGAACGGTGGACCG |  |
| 058210-P5 | GCGTAGGCATTTTTCGCTTCT |  |
| 058210hph-F | CTTGAGCCTAAAATCCGCCG |  |
| 058210hph-R | CGGAGCATTCACTAGGCAAC |  |
| 058210-F2 | CAAAGAGTATTGCCAATGATGAATG | Verification of Δ*AFUB_058210* in *A. fumigatus* Δ*ku80* |
| 058210-R2 | TCACATTCTCCTTCGCTTACTGC |  |
| 058210-F3 | CGAGATCAAGCAGATCAACGG |  |
| 058210-R3 | CATTGAGGGTGTGTCGCCAT |  |
| 058210-F4 | CCATCCAGCATCCATCCCA |  |
| 058210-R4 | CTTCCTCCCTGTCCTTCCTCA |  |
| 065800-P2 | CCAATAACGAATGCACAGCATAG | Deletion of *AFUB_065800* in *A. fumigatus* Δ*ku80* |
| 065800-P3 | CGGCGGATTTTAGGCTCAAGATCAGAGTACAAGGAAGTTGAGTCG |  |
| 065800-P4 | GTTGCCTAGTGAATGCTCCGGTCACACTTGCCTGTCAAACTCC |  |
| 065800-P5 | GCCTCCTCCTCTGGCTGATG |  |
| 065800hph-F | CTTGAGCCTAAAATCCGCCG |  |
| 065800hph-R | CGGAGCATTCACTAGGCAAC |  |
| 065800-F2 | TCAGTATGTCTGTTCGATCAGGG | Verification of Δ*AFUB_065800* in *A. fumigatus* Δ*ku80* |
| 065800-R2 | TCACATTCTCCTTCGCTTACTGC |  |
| 065800-F3 | CGAGATCAAGCAGATCAACGG |  |
| 065800-R3 | CCATCAAACCCGTCAGAACTC |  |
| 065800-F4 | TCCGCCGAGTTCTTCGAGA |  |
| 065800-R4 | TGCTGGTCAACAGTTCCGTGT |  |
| 066690-P2 | CGTCGATACAGAATACATCGCTG | Deletion of *AFUB_066690* in *A. fumigatus* Δ*ku80* |
| 066690-P3 | CGGCGGATTTTAGGCTCAAGATTTTCTTTTTTCAAGGTTGGATG |  |
| 066690-P4 | GTTGCCTAGTGAATGCTCCGGACGTTGGTAGAACTGCCATACC |  |
| 066690-P5 | CTCACCCATTCCACAAGACATTC |  |
| 066690hph-F | CTTGAGCCTAAAATCCGCCG |  |
| 066690hph-R | CGGAGCATTCACTAGGCAAC |  |
| 066690-F2 | CGGTAGTTCTTTGCTTGTGGATG | Verification of Δ*AFUB_066690* in *A. fumigatus* Δ*ku80* |
| 066690-R2 | TCACATTCTCCTTCGCTTACTGC |  |
| 066690-F3 | CGAGATCAAGCAGATCAACGG |  |
| 066690-R3 | GTGTCTGTTGACGGTGTTGAGG |  |
| 066690-F4 | AACACCTTCTGGGCTTCGG |  |
| 066690-R4 | GGGCAATCACCTCGTCGTAG |  |
| 071110-P2 | ACTAAGACGTTGCCATCGTGC | Deletion of *AFUB_071110* in *A. fumigatus* Δ*ku80* |
| 071110-P3 | CGGCGGATTTTAGGCTCAAGGCAGCGTAGGTTTGAACAGTAGATA |  |
| 071110-P4 | GTTGCCTAGTGAATGCTCCGTTGGAATGGAAGTGGTGATGG |  |
| 071110-P5 | TGGCAGGGGAGCGAGAGT |  |
| 071110hph-F | CTTGAGCCTAAAATCCGCCG |  |
| 071110hph-R | CGGAGCATTCACTAGGCAAC |  |
| 071110-F2 | GTTGTATCTTCTACGGCGGTCAT | Verification of Δ*AFUB_071110* in *A. fumigatus* Δ*ku80* |
| 071110-R2 | TCACATTCTCCTTCGCTTACTGC |  |
| 071110-F3 | CGAGATCAAGCAGATCAACGG |  |
| 071110-R3 | TGATGGGATTTTCCGACGC |  |
| 071110-F4 | GGAAGATCCGAATGGTTGGC |  |
| 071110-R4 | ACCGGCATGAGTAGATTTCACC |  |
| 073350-P2 | AAATAAACTAACAGTGCGGAGAATC | Deletion of *AFUB_073350* in *A. fumigatus* Δ*ku80* |
| 073350-P3 | CGGCGGATTTTAGGCTCAAGGGAGACCCGATATCAGAGAGACAT |  |
| 073350-P4 | GTTGCCTAGTGAATGCTCCGTCTCAGCCTAATCTTGTCATTCTCT |  |
| 073350-P5 | TTTTCCTCCCAATCGTCCG |  |
| 073350hph-F | CTTGAGCCTAAAATCCGCCG |  |
| 073350hph-R | CGGAGCATTCACTAGGCAAC |  |
| 073350-F2 | TTTATCAGGCTGAAGACCTAAAGTT | Verification of Δ*AFUB_073350* in *A. fumigatus* Δ*ku80* |
| 073350-R2 | TCACATTCTCCTTCGCTTACTGC |  |
| 073350-F3 | CGAGATCAAGCAGATCAACGG |  |
| 073350-R3 | TGATTGTATTCTCCAGTCAAAGCG |  |
| 073350-F4 | ACAGCGTCGGCACCACATAC |  |
| 073350-R4 | GTCTTGCCAACACTGACATTCG |  |
| 075110-P2 | GCTTTATGTTCTCTGGGTCGG | Deletion of *AFUB_075110* in *A. fumigatus* Δ*ku80* |
| 075110-P3 | CGGCGGATTTTAGGCTCAAGATTGATAGATGAGTGGGTGGTGTG |  |
| 075110-P4 | GTTGCCTAGTGAATGCTCCGTATCTATGATGTGGCAATCTATCGC |  |
| 075110-P5 | GCGTCTTGGAACGAGGAGTC |  |
| 075110hph-F | CTTGAGCCTAAAATCCGCCG |  |
| 075110hph-R | CGGAGCATTCACTAGGCAAC |  |
| 075110-F2 | TGAGAAGTGTCAAAAACAAGAAACC | Verification of Δ*AFUB_075110* in *A. fumigatus* Δ*ku80* |
| 075110-R2 | TCACATTCTCCTTCGCTTACTGC |  |
| 075110-F3 | CGAGATCAAGCAGATCAACGG |  |
| 075110-R3 | TGCTCACATCAGAAGAATTTTTCAT |  |
| 075110-F4 | TGTCATAGTGCCGTACTTCCTTCA |  |
| 075110-R4 | TCGTCTTGGTCGCATTGCTTA |  |
| 076500-P2 | GTGGAGCACAAGCACTGAGATAG | Deletion of *AFUB_076500* in *A. fumigatus* Δ*ku80* |
| 076500-P3 | CGGCGGATTTTAGGCTCAAGAAGCGTTATCTATTGGTGATGATGT |  |
| 076500-P4 | GTTGCCTAGTGAATGCTCCGTGTTGATGGGAAGCAACTCAGTAT |  |
| 076500-P5 | TGTCGGTCATCACATCATCACTT |  |
| 076500hph-F | CTTGAGCCTAAAATCCGCCG |  |
| 076500hph-R | CGGAGCATTCACTAGGCAAC |  |
| 076500-F2 | CAGTTATGTGTGATTGAAAAGATGG | Verification of Δ*AFUB_076500* in *A. fumigatus* Δ*ku80* |
| 076500-R2 | TCACATTCTCCTTCGCTTACTGC |  |
| 076500-F3 | CGAGATCAAGCAGATCAACGG |  |
| 076500-R3 | GCGATATTCCAGGCGATACC |  |
| 076500-F4 | CCCATTCCTTGCTTGGAGG |  |
| 076500-R4 | GCTGATTGAAGATTTGTGGTGC |  |
| 077400-P2 | TTACTGGCGAGGGTGGCAG | Deletion of *AFUB_077400* in *A. fumigatus* Δ*ku80* |
| 077400-P3 | CGGCGGATTTTAGGCTCAAGAATGTGGTAGAATAATAGTGACCCG |  |
| 077400-P4 | GTTGCCTAGTGAATGCTCCGTCTTGTCTCCTGGTTTGTATTCTTG |  |
| 077400-P5 | TCAATACAAGAGTAGGAAGCGTCG |  |
| 077400hph-F | CTTGAGCCTAAAATCCGCCG |  |
| 077400hph-R | CGGAGCATTCACTAGGCAAC |  |
| 077400-F2 | TCACCAAAACTTCCAGTCGTCA | Verification of Δ*AFUB_077400* in *A. fumigatus* Δ*ku80* |
| 077400-R2 | TCACATTCTCCTTCGCTTACTGC |  |
| 077400-F3 | CGAGATCAAGCAGATCAACGG |  |
| 077400-R3 | CTAGAACAATGTTGAAGTCCGATCT |  |
| 077400-F4 | CAGCGTTTGGGCGTAAGATT |  |
| 077400-R4 | GGAGTTGGTGATAAGGGAGGATT |  |
| 077440-P2 | ACAGAGTCGCTATTGGAAGATGG | Deletion of *AFUB_077440* in *A. fumigatus* Δ*ku80* |
| 077440-P3 | CGGCGGATTTTAGGCTCAAGGCGGATACGTAATAGGATGCAAG |  |
| 077440-P4 | GTTGCCTAGTGAATGCTCCGCGAGGGAAAGTACTCTGATTTAACG |  |
| 077440-P5 | TATCTGAGGTTCTTTCATCCATCAA |  |
| 077440hph-F | CTTGAGCCTAAAATCCGCCG |  |
| 077440hph-R | CGGAGCATTCACTAGGCAAC |  |
| 077440-F2 | TATGTGCAGTGTACGGCACATCT | Verification of Δ*AFUB_077440* in *A. fumigatus* Δ*ku80* |
| 077440-R2 | TCACATTCTCCTTCGCTTACTGC |  |
| 077440-F3 | CGAGATCAAGCAGATCAACGG |  |
| 077440-R3 | GATATGTTCTTGTCCTTTGTGGTGA |  |
| 077440-F4 | CGTCAACCCTGAGACACTCACC |  |
| 077440-R4 | ACCGATCTTCCACGACCACAT |  |
| 077850-P2 | GTAGCATTGCATCTGGACGAAG | Deletion of *AFUB_077850* in *A. fumigatus* Δ*ku80* |
| 077850-P3 | CGGCGGATTTTAGGCTCAAGGATAGTGGCTGGATGGTGATTG |  |
| 077850-P4 | GTTGCCTAGTGAATGCTCCGGTGGTGGAAGTATGCTTGTTACCA |  |
| 077850-P5 | AAACTGAGTGACATGGTGGATTAAC |  |
| 077850hph-F | CTTGAGCCTAAAATCCGCCG |  |
| 077850hph-R | CGGAGCATTCACTAGGCAAC |  |
| 077850-F2 | CGGCAGACAGACCCTTGAAC | Verification of Δ*AFUB_077850* in *A. fumigatus* Δ*ku80* |
| 077850-R2 | TCACATTCTCCTTCGCTTACTGC |  |
| 077850-F3 | CGAGATCAAGCAGATCAACGG |  |
| 077850-R3 | TATCTCAAGCTGCATGGGGTG |  |
| 077850-F4 | CGAGGGCGTCATCAGCAA |  |
| 077850-R4 | CAGAATATGTAGCCCAAAGTAGCG |  |
| 086810-P2 | CGTGCTGGAGATTCAGGAGAC | Deletion of *AFUB_086810* in *A. fumigatus* Δ*ku80* |
| 086810-P3 | CGGCGGATTTTAGGCTCAAGCTCGGAGTATCAAATCTGAGAATGA |  |
| 086810-P4 | GTTGCCTAGTGAATGCTCCGTTGTCATTCTTTCTATCTCTCTGGG |  |
| 086810-P5 | GAATTGTGGCATTCACATAGCG |  |
| 086810hph-F | CTTGAGCCTAAAATCCGCCG |  |
| 086810hph-R | CGGAGCATTCACTAGGCAAC |  |
| 086810-F2 | GCCATTCTCCTAACCCAGAGC | Verification of Δ*AFUB_086810* in *A. fumigatus* Δ*ku80* |
| 086810-R2 | TCACATTCTCCTTCGCTTACTGC |  |
| 086810-F3 | CGAGATCAAGCAGATCAACGG |  |
| 086810-R3 | GCGAGCTGGGTCTGGTCC |  |
| 086810-F4 | GCATGGGCACGCTTCTCAT |  |
| 086810-R4 | GCATTGTACGGCTCCACCAC |  |
| 087580-P2 | TAGATATCGACCTTCAACCACTACG | Deletion of *AFUB_087580* in *A. fumigatus* Δ*ku80* |
| 087580-P3 | CGGCGGATTTTAGGCTCAAGGAAGCGTGAGACTGACAAGGAG |  |
| 087580-P4 | GTTGCCTAGTGAATGCTCCGTGTCATTTTGCTTTGTTTAGTCTTG |  |
| 087580-P5 | ATAGTGCAACCGAAGGCGAA |  |
| 087580hph-F | CTTGAGCCTAAAATCCGCCG |  |
| 087580hph-R | CGGAGCATTCACTAGGCAAC |  |
| 087580-F2 | GTTATTGCGATTGATGGTGGG | Verification of Δ*AFUB_087580* in *A. fumigatus* Δ*ku80* |
| 087580-R2 | TCACATTCTCCTTCGCTTACTGC |  |
| 087580-F3 | CGAGATCAAGCAGATCAACGG |  |
| 087580-R3 | CATCCTTATCCATCCACCATCTT |  |
| 087580-F4 | CCACCGAGAAAGACGTTGACA |  |
| 087580-R4 | GGTCCGCAAGGCGAGTGA |  |
| 087590-P2 | AGTAGAGATTCGGGATTATTGCCT | Deletion of *AFUB_087590* in *A. fumigatus* Δ*ku80* |
| 087590-P3 | CGGCGGATTTTAGGCTCAAGGCAGTTAGCATCGAACACGTCC |  |
| 087590-P4 | GTTGCCTAGTGAATGCTCCGGTATGTTTCTTCCGTCACCTTCAGT |  |
| 087590-P5 | CCACAGTCTTCACTATTCCTCGC |  |
| 087590hph-F | CTTGAGCCTAAAATCCGCCG |  |
| 087590hph-R | CGGAGCATTCACTAGGCAAC |  |
| 087590-F2 | CTCTAGTTCGAATTGCGCGTC | Verification of Δ*AFUB_087590* in *A. fumigatus* Δ*ku80* |
| 087590-R2 | TCACATTCTCCTTCGCTTACTGC |  |
| 087590-F3 | CGAGATCAAGCAGATCAACGG |  |
| 087590-R3 | CCACGTACAAAGCGGAAAGC |  |
| 087590-F4 | GGCTGAACGGGTCCTGTCT |  |
| 087590-R4 | CAGCGATCTTGCCTTGTTCTATT |  |
| 089830-P2 | TGGAGCAACTTAGCAAGTCGG | Deletion of *AFUB_089830* in *A. fumigatus* Δ*ku80* |
| 089830-P3 | CGGCGGATTTTAGGCTCAAGTAAAATGAAGATAAATGGGACATGG |  |
| 089830-P4 | GTTGCCTAGTGAATGCTCCGCGATGTGTTAGTTCATAGAAGCCC |  |
| 089830-P5 | CCTGTTTTCAGTGACACCCTGT |  |
| 089830hph-F | CTTGAGCCTAAAATCCGCCG |  |
| 089830hph-R | CGGAGCATTCACTAGGCAAC |  |
| 089830-F2 | GTTATCAGCCTTCTTCCCTCGA | Verification of Δ*AFUB_089830* in *A. fumigatus* Δ*ku80* |
| 089830-R2 | TCACATTCTCCTTCGCTTACTGC |  |
| 089830-F3 | CGAGATCAAGCAGATCAACGG |  |
| 089830-R3 | CAGAAATCCATTATCAACAAGGTCC |  |
| 089830-F4 | TGCGGCTGAGACTGCGAA |  |
| 089830-R4 | CAACCAGAGTGAGCGAGACGA |  |
| 093370-P2 | GTTATTGAGGGCATCCCGTATC | Deletion of *AFUB_093370* in *A. fumigatus* Δ*ku80* |
| 093370-P3 | CGGCGGATTTTAGGCTCAAGGAGTTGGATAGGAAGACAAAGGATG |  |
| 093370-P4 | GTTGCCTAGTGAATGCTCCGGTCACAATCACTTTATGAAGCGAA |  |
| 093370-P5 | CGATAGCGATGATGACGAGGA |  |
| 093370hph-F | CTTGAGCCTAAAATCCGCCG |  |
| 093370hph-R | CGGAGCATTCACTAGGCAAC |  |
| 093370-F2 | ATAGTAGATTCATCTCACAAACGCC | Verification of Δ*AFUB_093370* in *A. fumigatus* Δ*ku80* |
| 093370-R2 | TCACATTCTCCTTCGCTTACTGC |  |
| 093370-F3 | CGAGATCAAGCAGATCAACGG |  |
| 093370-R3 | CGTTTAGGGGTGTGAGAGGC |  |
| 093370-F4 | TTAATGCCGCCAACAAGACC |  |
| 093370-R4 | GCGAACCAGTGCCAGAATG |  |
| 094100-P2 | TCTTTAGATGTAGGATTTACCAGCG | Deletion of *AFUB_094100* in *A. fumigatus* Δ*ku80* |
| 094100-P3 | CGGCGGATTTTAGGCTCAAGATCGTCAACAGATGGAGACTTAGTG |  |
| 094100-P4 | GTTGCCTAGTGAATGCTCCGAATGACTTTTTGCACATGGAGTTG |  |
| 094100-P5 | TACAGTATCCTCATTACGCCAACC |  |
| 094100hph-F | CTTGAGCCTAAAATCCGCCG |  |
| 094100hph-R | CGGAGCATTCACTAGGCAAC |  |
| 094100-F2 | TGGGGAAGACCTTTGGGC | Verification of Δ*AFUB_094100* in *A. fumigatus* Δ*ku80* |
| 094100-R2 | TCACATTCTCCTTCGCTTACTGC |  |
| 094100-F3 | CGAGATCAAGCAGATCAACGG |  |
| 094100-R3 | CCGAAGTCAATGAGATGATACAGAG |  |
| 094100-F4 | CGCTGGCAACAAGGTCGT |  |
| 094100-R4 | TTGACCCGTTGTAGCTCCTCA |  |
| 097740-P2 | GCCAATAAGAGAAACCAAAAAAGC | Deletion of *AFUB_097740* in *A. fumigatus* Δ*ku80* |
| 097740-P3 | CGGCGGATTTTAGGCTCAAGCAACTGGGATAATAAAGACGAGATG |  |
| 097740-P4 | GTTGCCTAGTGAATGCTCCGGCAATCGAAACAGTTATACCGC |  |
| 097740-P5 | GTCAGAAAAGAAAGGTTGCAGGA |  |
| 097740hph-F | CTTGAGCCTAAAATCCGCCG |  |
| 097740hph-R | CGGAGCATTCACTAGGCAAC |  |
| 097740-F2 | GCTCTCATAAGCAACCATCTCCT | Verification of Δ*AFUB_097740* in *A. fumigatus* Δ*ku80* |
| 097740-R2 | TCACATTCTCCTTCGCTTACTGC |  |
| 097740-F3 | CGAGATCAAGCAGATCAACGG |  |
| 097740-R3 | CGAAGAGTTGCCGTCTGTCATT |  |
| 097740-F4 | AAGCGTTACACCATCCCAGAG |  |
| 097740-R4 | CACAACCCTTCCATAAGTCCCT |  |
| 099750-P2 | TTAGTACCATGAGTGATTCCATTTG | Deletion of *AFUB_099750* in *A. fumigatus* Δ*ku80* |
| 099750-P3 | CGGCGGATTTTAGGCTCAAGGAGTGATTTGTATACTGTCAGTGCG |  |
| 099750-P4 | GTTGCCTAGTGAATGCTCCGAGTGGTAGTGGTAGGAGAGAGGATT |  |
| 099750-P5 | CAGCACATCGCAGACCCAG |  |
| 099750hph-F | CTTGAGCCTAAAATCCGCCG |  |
| 099750hph-R | CGGAGCATTCACTAGGCAAC |  |
| 099750-F2 | TCTCTTCCATCTCACATGAATCGT | Verification of Δ*AFUB_099750* in *A. fumigatus* Δ*ku80* |
| 099750-R2 | TCACATTCTCCTTCGCTTACTGC |  |
| 099750-F3 | CGAGATCAAGCAGATCAACGG |  |
| 099750-R3 | ACACCATGTACCTACTCACCAGTGT |  |
| 099750-F4 | CCTCGGCTGGGTCCAAGA |  |
| 099750-R4 | GAGTCCTGCATGGGCGTCT |  |
| OE7FlaggpdA-F | GCATGCGGAGAGACGGAC | Construction of SSP7-FLAG in *A. fumigatus* Δ*ku80* |
| OE7FlaggpdA-R | TTGTGAGACCACGGGCTCATTGGGAAAAGAAAGAGAAAAGAAAAG |  |
| OE7Flagosp7-F | ATGAGCCCGTGGTCTCACAA |  |
| OE7Flagosp7-R | AAGACCCCTTTGTAAGGTCCAA |  |
| OE7Flag-F | GGACCTTACAAAGGGGTCTTCTCGAGGGATCCCCGGGAATG |  |
| OE7Flag-R | TCAGAGTAAAGAAGAGGAGC |  |
| SSP7Flag-F2 | GCATGCGGAGAGACGGAC | Verification of SSP7-FLAG in *A. fumigatus* Δ*ku80* |
| SSP7Flag-R2 | TCAGAGTAAAGAAGAGGAGC |  |
| SSP8Flag-5F | CCAACACTGAACGACTTATACCCAG | Construction of SSP8-FLAG in *A. fumigatus* Δ*ku80* |
| SSP8Flag-5R | ATTCCCGGGGATCCCTCGAGCTGCTCGAAGCGTACCCAG |  |
| Flag-F | CTCGAGGGATCCCCGGGAATG |  |
| Flag-R | TCAGAGTAAAGAAGAGGAGC |  |
| SSP8Flag-3F | GCTCCTCTTCTTTACTCTGATGCCAGAGTTGAGTTCTCCTCTTT |  |
| SSP8Flag-3R | CGCCATAGCATTTCCTCTTCA |  |
| SSP8Flag-F2 | ATGAGTGTATGGACGGGGATG | Verification of SSP8-FLAG in *A. fumigatus* Δ*ku80* |
| SSP8Flag-R2 | CGCCATAGCATTTCCTCTTCA |  |
| r026560-F | atctcttgtcaacatgccgc | *ssp7* complementation in Δ*ssp7* strain of *A. fumigatus* Δ*ku80* |
| r026560-R | AATCAATTGCCCGTCTGTCAttcgtttgccttccttgacg |  |
| r026560bleo-F | TGACAGACGGGCAATTGATTA |  |
| r026560bleo-R | AGGAGGGCGTGAATGTAAGC |  |
| ORF1ssp7^2A^-F | TAACAGTAAGCAAGAGCCCGTGGTC | Point mutation of *ssp7*^T2A^ in *A. fumigatus* Δ*ku80* |
| ORF1ssp7^2A^-R | GACCACGGGCTCTTGCTTACTGTTA |  |
| ORF2ssp7^108A^-F | GGCTTTGAAGCAAGACAATCCGACC | Point mutation of *ssp7*^T108A^ in *A. fumigatus* Δ*ku80* |
| ORF2ssp7^108A^-R | GGTCGGATTGTCTTGCTTCAAAGCC |  |
| ORF3ssp7^240A^-F | GCGTGGTTTGTAAGATTTCTTCCGG | Point mutation of *ssp7*^T240A^ in *A. fumigatus* Δ*ku80* |
| ORF3ssp7^240A^-R | CCGGAAGAAATCTTACAAACCACGC |  |
| ORF4ssp7^184A^-F | ATTGCCTAGTTAAGATACCACTGAT | Point mutation of *ssp7*^T184A^ in *A. fumigatus* Δ*ku80* |
| ORF4ssp7^184A^-R | ATCAGTGGTATCTTAACTAGGCAAT |  |
| r059960-F | GATCTCTCCAAAGATCTGCACGA | *ssp8* complementation in Δ*ssp8* strain of *A. fumigatus* Δ*ku80* |
| r059960-R | CCTCTTCAATCGGGAAGGAGT |  |
| r059960bleo-F | CTCCTTCCCGATTGAAGAGGTGACAGACGGGCAATTGATTA |  |
| r059960bleo-R | AGGAGGGCGTGAATGTAAGC |  |
| ORF1ssp8^2A^-F | TTCGGCATATAAAGAGTGTATGGAC | Point mutation of *ssp8*^T2A^ in *A. fumigatus* Δ*ku80* |
| ORF1ssp8^2A^-R | GTCCATACACTCTTTATATGCCGAA |  |
| ORF2ssp8^729A^-F | ACTTCAGGCAGAAGCCTGCGGTTGG | Point mutation of *ssp8*^T729A^ in *A. fumigatus* Δ*ku80* |
| ORF2ssp8^729A^-R | CCAACCGCAGGCTTCTGCCTGAAGT |  |
| ORF3ssp8^10A^-F | ATAATGAGTGTAAGGACGGGGATGC | Point mutation of *ssp8*^T10A^ in *A. fumigatus* Δ*ku80* |
| ORF3ssp8^10A^-R | GCATCCCCGTCCTTACACTCATTAT |  |
| ORF4ssp8^145A^-F | GTCTGCTGATAAAGAATGATTCACA | Point mutation of *ssp8*^T145A^ in *A. fumigatus* Δ*ku80* |
| ORF4ssp8^145A^-R | TGTGAATCATTCTTTATCAGCAGAC |  |
| LncSSP7-F | TAATACGACTCACTATAGGGATGAGCCCGTGGTCTCACAA | *SSP7* sense strand *in vitro* transcription |
| LncSSP7-R | CTAAAGACCCCTTTGTAAGGTCCA |  |
| RLncSSP7-F | TAATACGACTCACTATAGGGCTAAAGACCCCTTTGTAAGGTCCA | *SSP7* antisense strand *in vitro* transcription |
| RLncSSP7-R | ATGAGCCCGTGGTCTCACAA |  |
| LncSSP8-F | TAATACGACTCACTATAGGGATGAGTGTATGGACGGGGATG | *SSP8* sense strand *in vitro* transcription |
| LncSSP8-R | CTACTGCTCGAAGCGTACCCA |  |
| RLncSSP8-F | TAATACGACTCACTATAGGGCTACTGCTCGAAGCGTACCCA | *SSP8* antisense strand *in vitro* transcription |
| RLncSSP8-R | ATGAGTGTATGGACGGGGATG |  |
| k293ssp7-P2 | atctcttgtcaacatgccgc | Deletion of *ssp7* in *A. fumigatus* Af293 |
| k293ssp7-P3 | CGGCGGATTTTAGGCTCAAGagtcccgtttcttagtcccg |  |
| k293ssp7-P4 | GTTGCCTAGTGAATGCTCCGatgcttcgctgtaccaatgg |  |
| k293ssp7-P5 | ttcgtttgccttccttgacg |  |
| k293ssp7hph-F | CTTGAGCCTAAAATCCGCCG |  |
| k293ssp7hph-R | CGGAGCATTCACTAGGCAAC |  |
| k293ssp7-F2 | aagcgccattggtttcgatt | Verification of Δ*ssp7* in *A. fumigatus* Af293 |
| k293ssp7-R2 | TCACATTCTCCTTCGCTTACTGC |  |
| k293ssp7-F3 | CGAGATCAAGCAGATCAACGG |  |
| k293ssp7-R3 | aagatacttgtcaagcgccc |  |
| k293ssp7-F4 | GTCTCACAAGGCTGAACCAACA |  |
| k293ssp7-R4 | AAATTACCGAGGATGAGAAGATAGG |  |
| 293R7-F | atctcttgtcaacatgccgc | *ssp7* complementation in Δ*ssp7* strain of *A. fumigatus* Af293 |
| 293R7-R | AATCAATTGCCCGTCTGTCAttcgtttgccttccttgacg |  |
| 293R7bleo-F | TGACAGACGGGCAATTGATTA |  |
| 293R7bleo-R | AGGAGGGCGTGAATGTAAGC |  |
| K26570(p1)-P2 | AACTTGTGCATTATGTCCTTTGCT | Δ*pprA*-1 in *A. fumigatus* Δ*ku80* |
| K26570(p1)-P3 | CGGCGGATTTTAGGCTCAAGCTTCAGGTCTTGACCCTACCGTA |  |
| K26570(p1)-P4 | GTTGCCTAGTGAATGCTCCGGAGTGGTGGTGTTTTTTTTGTACC |  |
| K26570(p1)-P5 | TTGAGAATGTTGATATTCCTGCG |  |
| 026570hph-F | CTTGAGCCTAAAATCCGCCG |  |
| 026570hph-R | CGGAGCATTCACTAGGCAAC |  |
| 026570(p1)-F2 | ATGGTGTATCAGGGTAATGGTCAG | Verification of Δ*pprA*-1 in *A. fumigatus* Δ*ku80* |
| 026570(p1)-R2 | TCACATTCTCCTTCGCTTACTGC |  |
| 026570(p1)-F3 | CGAGATCAAGCAGATCAACGG |  |
| 026570(p1)-R3 | TTTACAGGCACGGGTCTTCAA |  |
| 026570(p1)-F4 | TCGAGGTTGGCTGATGCG |  |
| 026570(p1)-R4 | GCTTATGGTCCTGGTAGCGTTT |  |
| K26570(p2)-P2 | TGTAGCATGTGGCTTGACTGTG | Δ*pprA*-2 in *A. fumigatus* Δ*ku80* |
| K26570(p2)-P3 | CGGCGGATTTTAGGCTCAAGAGTCGACAGTCAGCAACCGTG |  |
| K26570(p2)-P4 | GTTGCCTAGTGAATGCTCCGGTCCAAAAACCCGTTTGATTTG |  |
| K26570(p2)-P5 | ATTCCACCCTGCAAGACCAC |  |
| 026570hph-F | CTTGAGCCTAAAATCCGCCG |  |
| 026570hph-R | CGGAGCATTCACTAGGCAAC |  |
| 026570(p2)-F2 | TATTGAGCCCCTCTCATCGATAC | Verification ofΔ*pprA*-2 in *A. fumigatus* Δ*ku80* |
| 026570(p2)-R2 | TCACATTCTCCTTCGCTTACTGC |  |
| 026570(p2)-F3 | CGAGATCAAGCAGATCAACGG |  |
| 026570(p2)-R3 | CTAAGAGAACAATGCATGGGAATT |  |
| 026570(p2)-F4 | CACATTGCGATGAGCTGTTGTC |  |
| 026570(p2)-R4 | TGTAGTGTTTGCACGAGGTTAAGATA |  |
| Δssp7ΔpprA(1)-P2 | atctcttgtcaacatgccgc | Δ*pprA*-1 in Δ*ssp7* strain of *A. fumigatus* Δ*ku80* |
| Δssp7ΔpprA(1)-P3 | AATCAATTGCCCGTCTGTCAagtcccgtttcttagtcccg |  |
| Δssp7ΔpprA(1)-P4 | GCTTACATTCACGCCCTCCTGAGTGGTGGTGTTTTTTTTGTACC |  |
| Δssp7ΔpprA(1)-P5 | TTGAGAATGTTGATATTCCTGCG |  |
| Δssp7ΔpprA(1)bleo-F | TGACAGACGGGCAATTGATTA |  |
| Δssp7ΔpprA(1)bleo-R | AGGAGGGCGTGAATGTAAGC |  |
| Δssp7ΔpprA(1)-F2 | aagcgccattggtttcgatt | Verification of Δ*pprA*-1 in Δ*ssp7* strain of *A. fumigatus* Δ*ku80* |
| Δssp7ΔpprA(1)-R2 | AGGAGGGCGTGAATGTAAGC |  |
| Δssp7ΔpprA(1)-F3 | TGACAGACGGGCAATTGATTA |  |
| Δssp7ΔpprA(1)-R3 | TTTACAGGCACGGGTCTTCAA |  |
| Δssp7ΔpprA(1)-F4 | TCGAGGTTGGCTGATGCG |  |
| Δssp7ΔpprA(1)-R4 | GCTTATGGTCCTGGTAGCGTTT |  |
| Δssp7ΔpprA(2)-P2 | TGGCTTCTTGCAGAAAACCAAT | Δ*pprA*-2 in Δ*ssp7* strain of *A. fumigatus* Δ*ku80* |
| Δssp7ΔpprA(2)-P3 | AATCAATTGCCCGTCTGTCAagtcccgtttcttagtcccg |  |
| Δssp7ΔpprA(2)-P4 | GCTTACATTCACGCCCTCCTGACACCCAACTCGTGCGTG |  |
| Δssp7ΔpprA(2)-P5 | TCCTGGAATGCCATGACCTG |  |
| Δssp7ΔpprA(2)bleo-F | TGACAGACGGGCAATTGATTA |  |
| Δssp7ΔpprA(2)bleo-R | AGGAGGGCGTGAATGTAAGC |  |
| Δssp7ΔpprA(2)-F2 | aagcgccattggtttcgatt | Verification of Δ*pprA*-2 in Δ*ssp7* strain of *A. fumigatus* Δ*ku80* |
| Δssp7ΔpprA(2)-R2 | AGGAGGGCGTGAATGTAAGC |  |
| Δssp7ΔpprA(2)-F3 | TGACAGACGGGCAATTGATTA |  |
| Δssp7ΔpprA(2)-R3 | CAAATCAAACGGGTTTTTGGAC |  |
| Δssp7ΔpprA(2)-F4 | ATGCTCGCCCGATGCTGT |  |
| Δssp7ΔpprA(2)-R4 | TTGCCTTCCTTGACGCTCT |  |
| 26570Flag-5F | TCTTCGCACTCTTGAATCAAACTAC | Construction of PprA-FLAG in *A. fumigatus* Δ*ku80* |
| 26570Flag-5R | ATTCCCGGGGATCCCTCGAGGTCGGCCTCGTCAGTGTTCT |  |
| 26570Flag-F | CTCGAGGGATCCCCGGGAATG |  |
| 26570Flag-R | TCAGAGTAAAGAAGAGGAGC |  |
| 26570Flag-3F | GCTCCTCTTCTTTACTCTGATCCAAAAACCCGTTTGATTTG |  |
| 26570Flag-3R | GTCCTACAAGGCCTCTGAGCC |  |
| 26570EGFP-5F | TCTTCGCACTCTTGAATCAAACTAC | Construction of PprA-GFP in *A. fumigatus* Δ*ku80* |
| 26570EGFP-5R | ATTCCCGGGGATCCCTCGAGGTCGGCCTCGTCAGTGTTCT |  |
| 26570EGFP-F | CTCGAGGGATCCCCGGGAATGATGGTGAGCAAGGGCGAG |  |
| 26570EGFP-R | TTACTTGTACAGCTCGTCCATGC |  |
| 26570EGFPhph-F | TGGACGAGCTGTACAAGTAATAGCCCGGGTAAAAGGAATGT |  |
| 26570EGFPhph-R | TCAGAGTAAAGAAGAGGAGCATGTC |  |
| 26570EGFP-3F | GCTCCTCTTCTTTACTCTGATCCAAAAACCCGTTTGATTTG |  |
| 26570EGFP-3R | GTCCTACAAGGCCTCTGAGCC |  |
| k7(570Flag/GFP)-P2 | atctcttgtcaacatgccgc | Deletion of *ssp7* in the PprA-tagged background |
| k7(570Flag/GFP)-P3 | AATCAATTGCCCGTCTGTCAagtcccgtttcttagtcccg |  |
| k7(570Flag/GFP)-P4 | GCTTACATTCACGCCCTCCTatgcttcgctgtaccaatgg |  |
| k7(570Flag/GFP)-P5 | ttcgtttgccttccttgacg |  |
| k7(570Flag/GFP)bleo-F | TGACAGACGGGCAATTGATTA |  |
| k7(570Flag/GFP)bleo-R | AGGAGGGCGTGAATGTAAGC |  |
| k7(570Flag/GFP)-F2 | aagcgccattggtttcgatt | Verification of Δ*ssp7* in the PprA-tagged background |
| k7(570Flag/GFP)-R2 | AGGAGGGCGTGAATGTAAGC |  |
| k7(570Flag/GFP)-F3 | TGACAGACGGGCAATTGATTA |  |
| k7(570Flag/GFP)-R3 | aagatacttgtcaagcgccc |  |
| k7(570Flag/GFP)-F4 | GTCTCACAAGGCTGAACCAACA |  |
| k7(570Flag/GFP)-R4 | AAATTACCGAGGATGAGAAGATAGG |  |
| SSP7_235-278_scramble-F | TGCACTGCTACTTGGCATCTTGAGTGCCATCTACGTCCTATCTTCTCATCCTCGG | Scramble mutation of *ssp7*^235-278^ in *A. fumigatus* Δ*ku80* |
| SSP7_235-278_scramble-R | AGATGCCAAGTAGCAGTGCAACGAGTATCCGAACCACGCCCAAAAAAAAAGT |  |
| SSP7_333-378_scramble-F | GTTGTATGTTCCAATCTAGTGTTTATACATTCCAACATTCGAAATCGACGATTGC | Scramble mutation of *ssp7*^333-378^ in *A. fumigatus* Δ*ku80* |
| SSP7_333-378_scramble-R | ACTAGATTGGAACATACAACCGATTCCAAAAGGCTTTCGCAGAAACAGTAATCAAAAA |  |
| R26570-F | AACTTGTGCATTATGTCCTTTGCT | *pprA* complementation in Δ*pprA*-1 andΔ*pprA*-2 strains of *A. fumigatus* Δ*ku80* |
| R26570-R | AATCAATTGCCCGTCTGTCAGCATACCTCCCATCAGGTACTTG |  |
| R26570phel-F | TGACAGACGGGCAATTGATTA |  |
| R26570phel-R | AGGAGGGCGTGAATGTAAGC |  |
| r026560(ptrA)-F | atctcttgtcaacatgccgc | *ssp7* complementation in the Δ*ssp7*_PprA-FLAG background |
| r026560(ptrA)-R | ATTCCCGGGGATCCCTCGAGttcgtttgccttccttgacg |  |
| ptrA-F | CTCGAGGGATCCCCGGGAATG |  |
| ptrA-R | GACATTCCTTTTACCCGGGC |  |
